# Empirical Bayes Estimation of Semi-parametric Hierarchical Mixture Models for Unbiased Characterization of Polygenic Disease Architectures

**DOI:** 10.3389/fgene.2018.00115

**Published:** 2018-04-24

**Authors:** Jo Nishino, Yuta Kochi, Daichi Shigemizu, Mamoru Kato, Katsunori Ikari, Hidenori Ochi, Hisashi Noma, Kota Matsui, Takashi Morizono, Keith A. Boroevich, Tatsuhiko Tsunoda, Shigeyuki Matsui

**Affiliations:** ^1^Department of Medical Science Mathematics, Medical Research Institute, Tokyo Medical and Dental University (TMDU), Tokyo, Japan; ^2^Core Research for Evolutionary Science and Technology (CREST), Japan Science and Technology Agency (JST), Tokyo, Japan; ^3^Laboratory for Autoimmune Diseases, RIKEN Center for Integrative Medical Sciences, Yokohama, Japan; ^4^Division of Genomic Medicine, Medical Genome Center, National Center for Geriatrics and Gerontology, Obu, Japan; ^5^Laboratory for Medical Science Mathematics, RIKEN Center for Integrative Medical Sciences, Yokohama, Japan; ^6^Department of Bioinformatics, National Cancer Center Research Institute, Tokyo, Japan; ^7^Institute of Rheumatology, Tokyo Women's Medical University, Tokyo, Japan; ^8^Division of Frontier Medical Science, Department of Gastroenterology and Metabolism, Programs for Biomedical Research Graduate School of Biomedical Science, Hiroshima University, Hiroshima, Japan; ^9^Laboratory for Digestive Diseases, RIKEN Center for Integrative Medical Sciences, Hiroshima, Japan; ^10^Department of Data Science, The Institute of Statistical Mathematics, Tokyo, Japan; ^11^Department of Biostatistics, Nagoya University Graduate School of Medicine, Nagoya, Japan; ^12^Risk Analysis Research Center, The Institute of Statistical Mathematics, Tokyo, Japan

**Keywords:** genome-wide association study (GWAS), polygenic disease architecture, polygenicity, effect-size distribution, semi-parametric hierarchical mixture model

## Abstract

Genome-wide association studies (GWAS) suggest that the genetic architecture of complex diseases consists of unexpectedly numerous variants with small effect sizes. However, the polygenic architectures of many diseases have not been well characterized due to lack of simple and fast methods for unbiased estimation of the underlying proportion of disease-associated variants and their effect-size distribution. Applying empirical Bayes estimation of semi-parametric hierarchical mixture models to GWAS summary statistics, we confirmed that schizophrenia was extremely polygenic [~40% of independent genome-wide SNPs are risk variants, most within odds ratio (OR = 1.03)], whereas rheumatoid arthritis was less polygenic (~4 to 8% risk variants, significant portion reaching OR = 1.05 to 1.1). For rheumatoid arthritis, stratified estimations revealed that expression quantitative loci in blood explained large genetic variance, and low- and high-frequency derived alleles were prone to be risk and protective, respectively, suggesting a predominance of deleterious-risk and advantageous-protective mutations. Despite genetic correlation, effect-size distributions for schizophrenia and bipolar disorder differed across allele frequency. These analyses distinguished disease polygenic architectures and provided clues for etiological differences in complex diseases.

## Introduction

Genome-wide association studies (GWAS) have identified numerous susceptibility variants for complex diseases (Welter et al., 2014). The sets of variants identified from GWAS, however, can generally explain only a small proportion of the heritability estimated from family studies, the so called “missing heritability” problem (Manolio et al., 2009). Much research has suggested that the variance explained by all SNPs in dense genotyping arrays, i.e., SNP heritability, often accounts for a large proportion of the family-based heritability (Lee et al., 2011, 2012, 2013; So et al., 2011b; Stahl et al., 2012; Ripke et al., 2013; Golan et al., 2014; Bulik-Sullivan et al., 2015; Palla and Dudbridge, 2015).

Quantitative evaluation of the polygenic architecture, in particular, the estimation of the proportion of disease-associated SNPs and their effect-size distribution, is essential to further determine the source of observed heritability (Wray et al., 2007; Park et al., 2010, 2011; Stahl et al., 2012; Agarwala et al., 2013; Chatterjee et al., 2013; Ripke et al., 2013). The estimation of these components also contributes to accurate power and sample size calculations of GWAS (Wray et al., 2007, 2012; Park et al., 2010; Yang et al., 2010; Ripke et al., 2013; Levinson et al., 2014) and estimation of the predictive capability of disease risks (Wray et al., 2007; Agarwala et al., 2013; Chatterjee et al., 2013).

However, we are still far from understanding the polygenic architecture of most complex diseases, because so far, there have been no feasible or fast methods that unbiasedly evaluate various polygenic architectures using the entire set of SNPs across the genome. Stahl et al. proposed estimating the proportion of disease-associated SNPs and the effect-size distribution using an approximate Bayesian polygenic analysis (Stahl et al., 2012). Its application, however, has been limited to few studies (Stahl et al., 2012; Ripke et al., 2013) because of the technical complexity and the excess computational burden of many simulations. On the other hand, some authors estimated the effect-size distribution based on a power evaluation for SNPs reaching genome-wide significance (Park et al., 2010, 2011; Chatterjee et al., 2013). This method, however, is to evaluate effect sizes only for those SNPs with relatively large effects, not all the disease-associated SNPs, requiring adjustment for the winner's curse (selection bias in using top significant SNPs) in the effect-size estimation.

To address the aforementioned limitations of existing methods, we propose an empirical Bayes estimation of semi-parametric hierarchical mixture models (SP-HMMs) (Matsui and Noma, 2011a,b) of GWAS summary statistics on effect sizes, such as estimated log-odds ratios to associate genotypes with disease susceptibility (see section Materials and Methods). This model decomposes GWAS summary data into signal and noise components and derives the proportion of disease-associated SNPs (non-null SNPs) and the distribution of their effect sizes (genotype log-odds ratios) as the signal component. To be more specific, mixture modeling refers to decomposing the underlying distribution of SNP-specific summary statistics into a non-null distribution for SNPs associated with disease occurrence, which corresponds to a signal component, and a null distribution for the remaining SNPs without association, which corresponds to a noise component, with a mixing probability or proportion of disease-associated SNPs, π. For the non-null distribution, semi-parametric hierarchical modeling incorporates standard asymptotic normality for summary statistics, while the true effect sizes follow a non-parametric prior distribution, *g*. With an expectation-maximization (EM) algorithm (Shen and Louis, 1999), we can estimate the prior probability π and distribution *g* using the data, i.e., empirical Bayes estimation. The empirical Bayes estimation of hierarchical mixture models is also applicable for SNP heritability estimation (So et al., 2011b) and adjustment for the winner's curse (Ferguson et al., 2013).

The features of our approach are summarized as follows: (1) the polygenic architecture for the entire set of SNPs, represented by π and *g*, can be flexibly and unbiasedly estimated, (2) it requires only summary data from GWAS (e.g., estimated log-odds ratios and standard error for individual SNPs are used), and (3) the estimation algorithm is easily implemented and fast. As such, the objective of the present study is to ascertain these features in evaluating the underlying the polygenic architecture of complex diseases, through its application to GWAS data from various diseases presumed to have distinct polygenic architecture in terms of several aspects.

Throughout this paper, we fit the SP-HMM to summary data from meta-/mega- GWAS analyses of rheumatoid arthritis (Okada et al., 2014), schizophrenia (Ripke et al., 2014), bipolar disorder (Sklar et al., 2011), and coronary artery disease [The (Coronary Artery Disease (C4D) Genetics Consortium., 2011; Schunkert et al., 2011)], to estimate the respective polygenic architectures and compare them across diseases. We also assess the liability-scale variance explained by SNPs, i.e., SNP heritability, based on this estimation. In order to obtain further insight into the underlying polygenic architectures, our approach can be applied to SNPs belonging to important functional categories, such as expression quantitative trait loci (eQTL), coding, non-synonymous, promoter, 5′ or 3′ UTR, enhancer, and DNase I hypersensitivity sites (Hindorff et al., 2009; Nicolae et al., 2010; Finucane et al., 2015; Gamazon et al., 2015). We focus on eQTLs, as gene expression levels have been increasingly recognized as notable endophenotypes or important mediators between genetic variations and disease phenotypes (Nicolae et al., 2010; Gusev et al., 2014; Gamazon et al., 2015; Zhu et al., 2016). Lastly, we also applied our method to GWAS data stratified by derived allele frequency (DAF), rather than minor allele frequency (MAF) (Park et al., 2011; Chan et al., 2014; Gorlov et al., 2015). A minor allele with low MAF can represent an allele with high DAF possibly under positive selection, as well as an allele that is maintained at low DAF through negative selection. Thus, our DAF-based analysis facilitates interpretation from the perspective of population genetics (Lachance, 2010), possibly contributing to further understanding of the genetic etiology for complex diseases.

## Results

**Figure 1:**
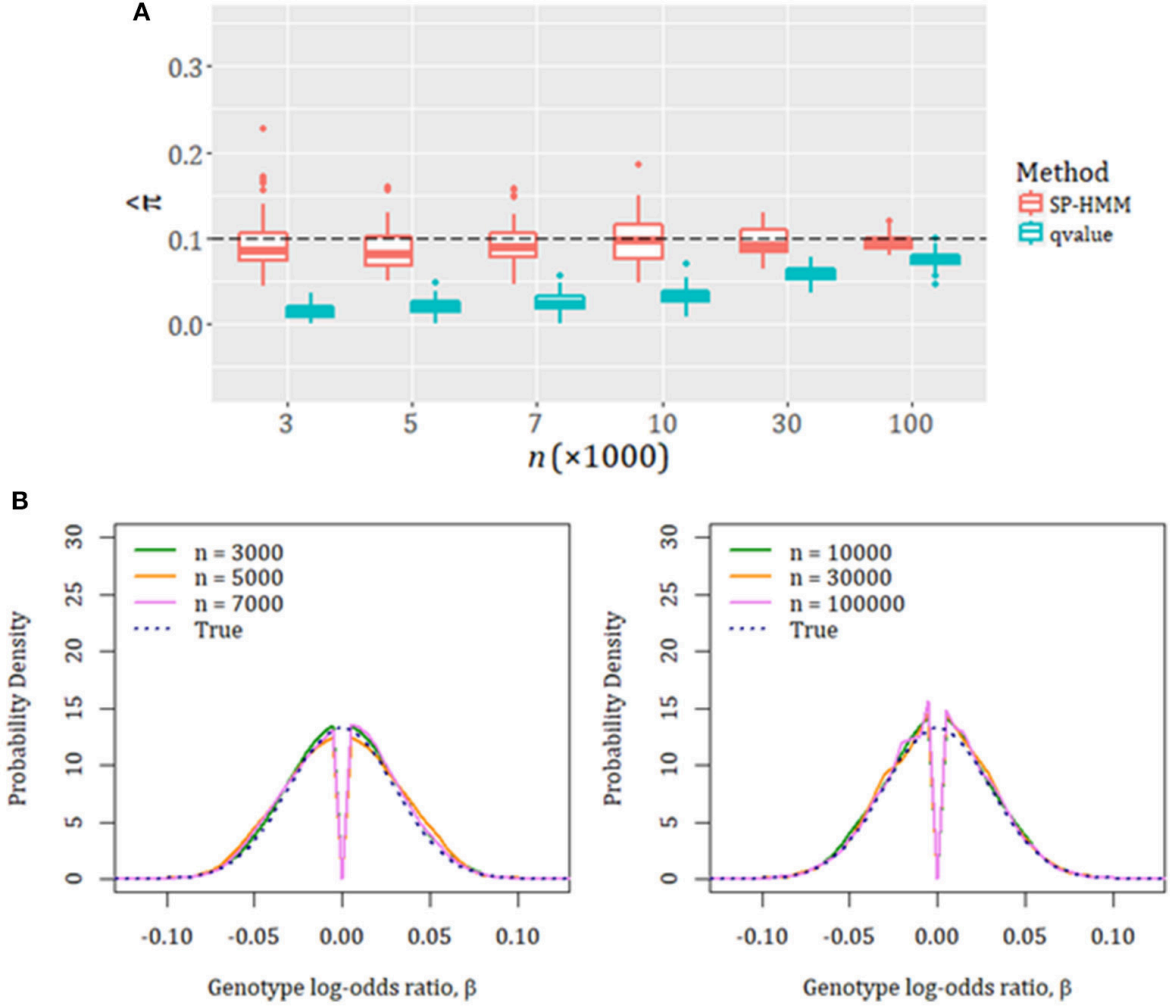
Evaluation of SP-HMM estimation by simulations. The true proportion of associated SNPs is set to be π = 0.1. Effect-size distribution of associated SNPs is the normal distribution with a variance of 0.03^2^. Various sample sizes (*n* = 3,000–100,000 cases and controls) were used. The total number of SNPs is 100,000. **(A)** Estimated proportion of disease-associated variants, $\hat{\pi }$. “qvalue”: results obtained by qvalue R package (Bioconductor v.3.4). **(B)** Estimated effect-size distributions, *ĝ*. Average curves over 100 simulations for each sample size are shown. The specified (true) effect-size distributions are given in dotted lines.

We first confirmed the adequacy of our estimation method through unbiased estimation of the proportion of disease-associated SNPs, π, and their effect-size distribution, *g*, in simulation experiments (Figure 1; For all simulation settings, see Tables S1, S2, and Figures S1–S17 in Supplementary Material). The non-parametric estimation for *g* could flexibly capture various forms of underlying effect-size distributions. The execution time for the estimation using real data with one-hundred thousand SNPs was adequately fast; it completed in less than 5 min (Table S7).

For application to real GWAS datasets, we used publicly available summary statistics from large meta-/mega-GWAS for the four complex diseases (see Tables S3, S4 in Supplementary Material for details of the GWAS data). In associating each genotype with disease susceptibility, we defined the effect size as a log-odds ratio of the derived allele relative to the ancestral allele, denoted by β. We obtained an estimate of β and its variance estimate from the summary data. The ancestral/derived alleles for each SNP were determined from dbSNP.

### Estimated proportion of disease-associated SNPs and effect-size distribution

To estimate the proportion of disease-associated SNPs, π, and the effect-size distribution, *g*, based on independent SNPs, we used two linkage disequilibrium (LD) pruned SNP sets: *P*-value-based and random-pruned sets. Note that we evaluated π and *g* with respect to the marginal effects of the nearly independent SNPs, as done by Stahl et al. (2012), rather than with respect to the effects of underlying causal variants themselves. The *P*-value-based method preferentially selected SNPs with stronger associations (hence more closely linked to causal variants) while using other GWAS data to correct for selection bias (see section Materials and Methods for details). The random-pruned method sampled SNPs randomly. In both methods, SNPs in LD (*r*^2^ > 0.1) were removed. In a case where causal variants are in LD, only one would be retained in the final prune set, and thus, the estimates $\hat{\pi }$ × (the number of SNPs in the SNP sets) using the pruned sets would give conservative estimates of the number of causal variants.

**Figure 2:**
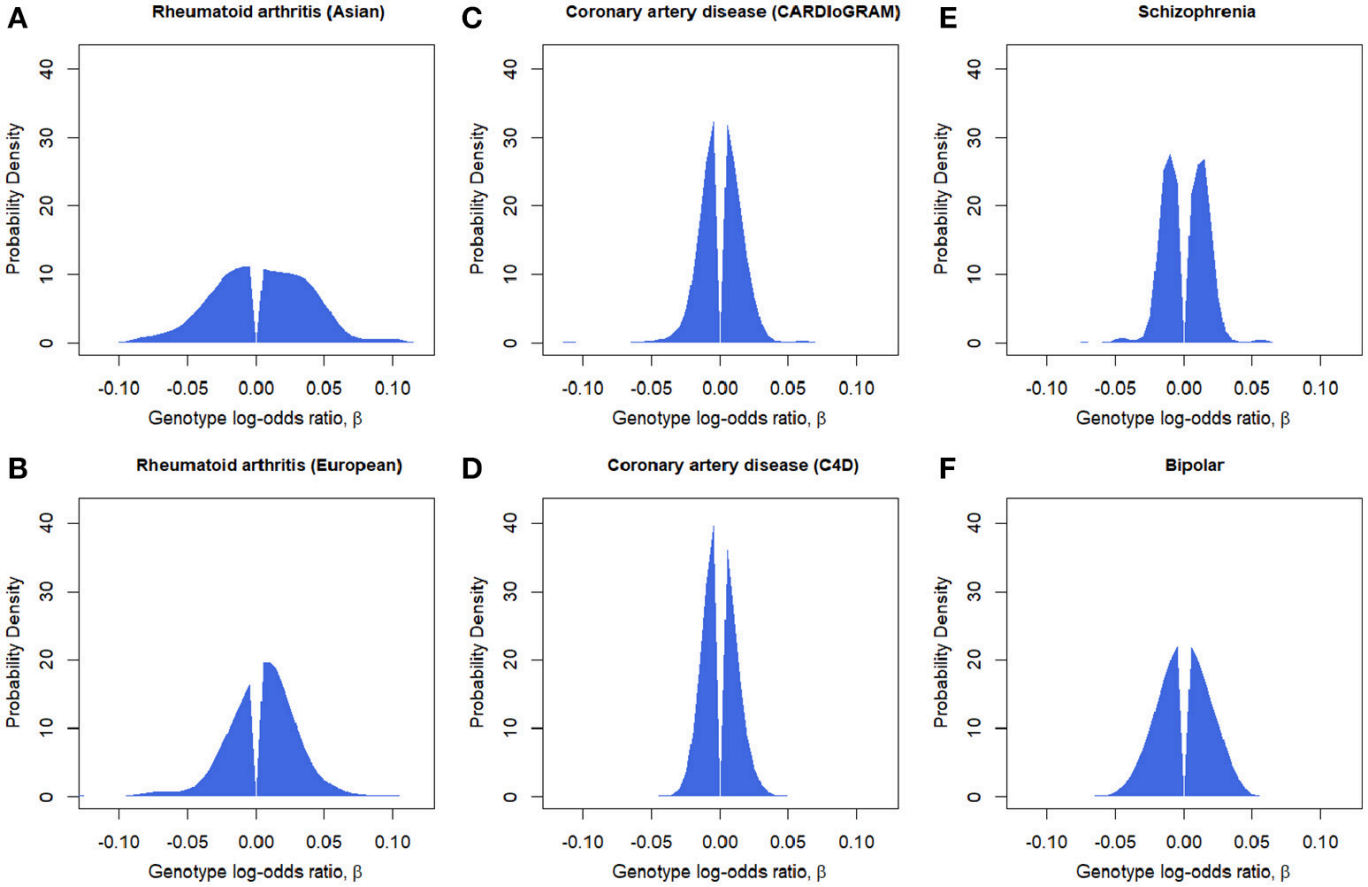
Estimated effect-size distributions for disease-associated SNPs, $\hat{g}$. *P*-value-based pruned SNP sets are used. **(A)** Rheumatoid arthritis (Asian). **(B)** Rheumatoid arthritis (European). **(C)** Coronary artery disease (CARDIoGRAM). **(D)** Coronary artery disease (C4D). **(E)** Schizophrenia. **(F)** Bipolar disorder.

We fit the SP-HMM to the *P*-value-based pruned SNP sets in each GWAS (Table 1; Figure 2). For rheumatoid arthritis, π was estimated as 3.6% for Asian and 8.1% for European populations, which were lower than the other diseases. The estimates of π were larger for two psychiatric diseases: 43.0% for schizophrenia and 39.6% for bipolar disorder. For coronary artery disease, using CARDIoGRAM and C4D data, π was estimated to be 15.9 and 26.1%, respectively.

**Table 1:** Estimated proportions of disease-associated SNPs, $\hat{\pi }$, and liability-scale variance explained by SNPs, $\hat{V}$.

	**$\hat{\pi }$ (SE^a^) (%)**	**$\hat{V}$ (SE^a^) (%)**
Rheumatoid arthritis (Asian)	3.6 (1.8)	14.0 (1.8)
Rheumatoid arthritis (European)	8.1 (2.4)	20.2 (1.5)
Coronary artery disease (CARDIoGRAM)	15.9 (3.7)	20.9 (1.3)
Coronary artery disease (C4D)	26.1 (3.5)	22.2 (1.4)
Schizophrenia	43.0 (1.1)	40.2 (0.7)
Bipolar disorder	39.6 (2.2)	50.0 (1.9)

*Estimates for the P-value-based SNP sets are shown*.

*For $\hat{V}$, disease prevalences are assumed to be 1% for rheumatoid arthritis and schizophrenia, 6% for coronary artery disease and 0.5% for bipolar disorder*.

a *Estimated based on 100 parametric bootstrap samples based on the estimated SP-HMM*.

With regard to the estimation of *g*, rheumatoid arthritis was shown to have a significant portion with larger effects, spanning to |β| = 0.05 (odds ratio of 0.95 or 1.05) or larger (Figure 2). It is noteworthy that, for rheumatoid arthritis, the proportion of positive effects was clearly larger than that of negative effects, indicating that the derived alleles are more likely to be risk alleles for the disease. Bipolar disorder was also estimated to have a distribution with relatively large effects. In contrast, schizophrenia and coronary artery disease was shown to have narrower distribution with very small effects. Schizophrenia was shown to have peaks around |β| = 0.05.

The estimates of π for the random-pruned SNP sets were similar to those for the *P*-value-based SNP sets for each GWAS (Table S5 in Supplementary Material). For the estimation of effect-size distribution, *ĝ*, the absolute effect size, |β|, tended to be slightly greater when using the *P*-value-based SNP sets than when using the random-pruned SNP set (Figure S19 in Supplementary Material).

### Liability-scale variance explained by the pruned SNP set

Using the estimates of the polygenic architecture (π and *g*), together with disease prevalence and allele frequencies, we could immediately evaluate the liability-scale variance, *V*, i.e., SNP heritability, explained by the pruned SNP set. Note that we evaluated *V* on the pruned SNP sets rather than on all SNPs on the GWAS chips. For evaluating *V*, the SP-HMM could directly model binary traits (i.e., disease occurrence) via log-odds ratios obtained from GWAS summary data.

Using the *P*-value-based pruned SNP sets, for rheumatoid arthritis, the estimates of *V* were 14.0% for Asian and 20.2% for European data (Table 1). Based on the estimated variance of 12% explained by the major histocompatibility complex (MHC) region (removed from the SNP set) and family based heritability of 55% (Supplementary Table 1 of Stahl et al., 2012), SNPs explained 47.3% (= (0.14 + 0.12)/0.55) and 58.2% (= (0.20 + 0.12)/0.55) of the family based heritability for the Asian and European populations, respectively, which were generally consistent with the previous estimate of 65% (Stahl et al., 2012). The estimates of *V* in schizophrenia and bipolar disorder were 40.2% and 50.0%, respectively, which were higher but almost within the range of previously reported estimates of 23-43% and 25-47%, respectively, for these diseases (Lee et al., 2012; Stahl et al., 2012; Ripke et al., 2013; Golan et al., 2014; Loh et al., 2015; Palla and Dudbridge, 2015). For cardiovascular disease, the estimates of *V* from the CARDIoGRAM and C4D data were 20.9 and 22.2%, respectively.

The estimates of *V* for the *P*-value-based pruned SNP sets (Table 1) were greater than those for the random-pruned SNP sets, but the differences were not substantial except for bipolar disorder (Table S5 in Supplementary Material).

### Stratified estimation for eQTL/non-eQTL-SNPs

**Figure 3:**
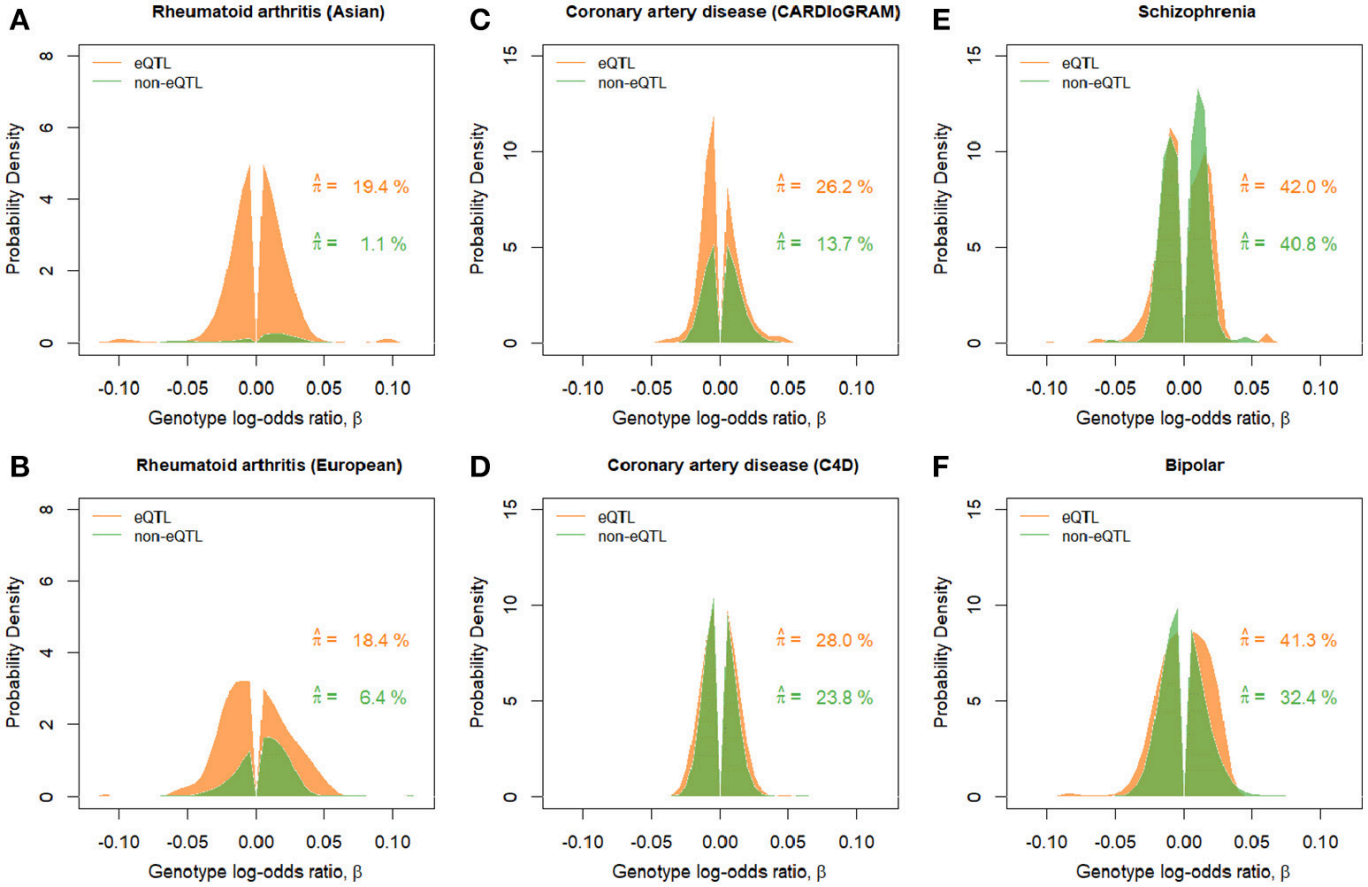
Estimated effect size distributions for eQTL-SNPs and non-eQTL-SNPs, $\hat{\pi }\times \hat{g}$. Green and orange graphs show the results for the eQTL-SNP and non-eQTL-SNP sets, respectively. Estimated proportion of disease-associated SNPs, $\hat{\pi }$, correspond to the areas under the curves. **(A)** Rheumatoid arthritis (Asian). **(B)** Rheumatoid arthritis (European). **(C)** Coronary artery disease (CARDIoGRAM). **(D)** Coronary artery disease (C4D). **(E)** Schizophrenia. **(F)** Bipolar disorder.

In order to gain insights into mediator effects of gene expression level, we fit the SP-HMM to “eQTL” SNPs, detected as cis-eQTLs using peripheral blood samples (Westra et al., 2013), and the remaining “non-eQTL”-SNPs, separately (Figure 3). All the SNPs in this analysis were selected to be nearly independent using a LD-pruning method based on LD (*r*^2^ > 0.1) (see section Materials and Methods).

For rheumatoid arthritis in Asian and European populations, the proportions of disease-associated SNPs in the eQTL-SNPs were estimated to be larger than that in the non-eQTL-SNPs (Figure 3). In addition, the estimated effect-size distributions in terms of π × *g* (frequencies in the entire set including both null and non-null SNPs) in Figure 3 indicated that there was a significant portion of SNPs with large effects, |β| > 0.05, for the eQTL-SNPs, but a small portion for the non-eQTL-SNPs, suggesting that the set of eQTL-SNPs included more components with distinctive large effects for rheumatoid arthritis. For the other diseases, there was a tendency for the frequencies of disease-associated SNPs in the set of eQTL-SNPs to be larger than those of the non-eQTL-SNPs.

We also estimated *V* for the eQTL-SNPs and non-eQTL-SNPs, separately (Table S6 in Supplementary Material). For rheumatoid arthritis, as expected from Figure 3, the per-SNP variance for the eQTL-SNPs was much larger than for the non-eQTL-SNPs. Interestingly, although eQTLs were defined using European samples (Westra et al., 2013), the enrichment of per-SNP variance (10.7-fold) in the eQTL-SNPs in the Asian population was larger than the 5.2-fold enrichment seen in the European population.

### Estimation across derived allele frequencies

**Figure 4:**
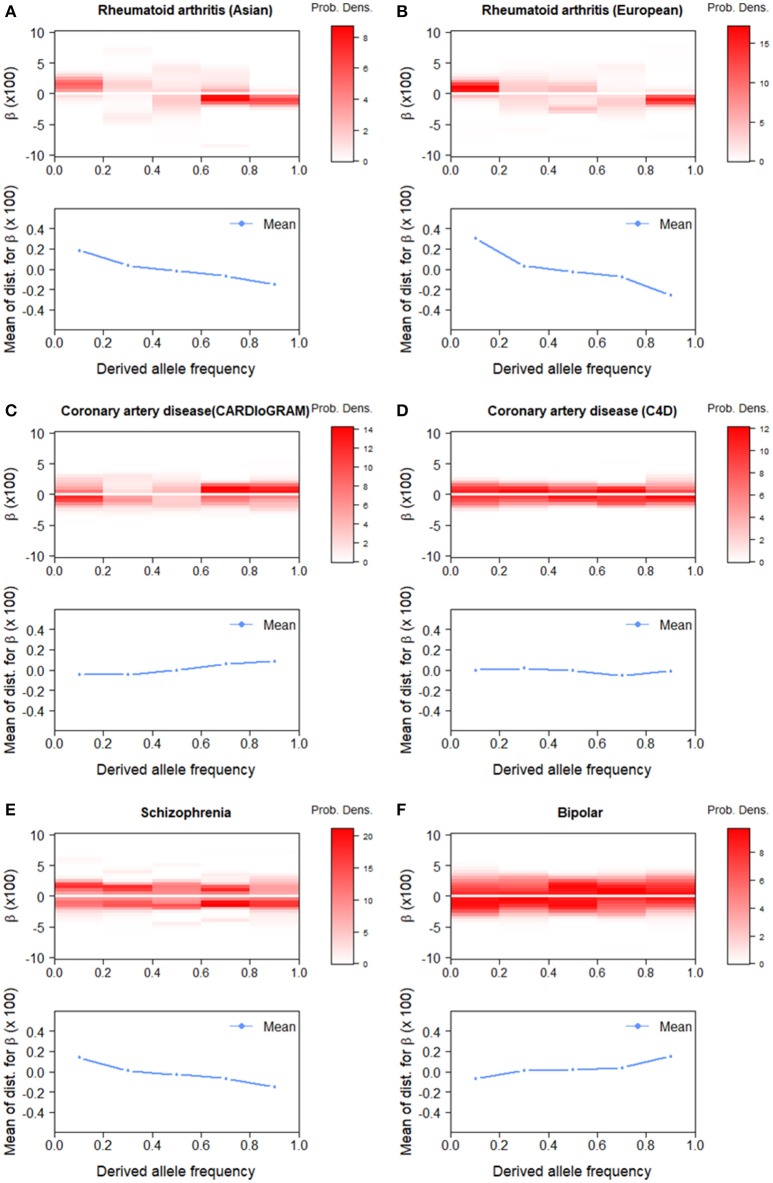
Estimated effect-size distributions, $\hat{\pi }\times \hat{g}$, by derived allele frequency (DAF) bins. The upper panels (heatmap colors) for each GWAS results show $\hat{\pi }\times ĝ$. The lower panels show means of $\hat{\pi }\times ĝ$. **(A)** Rheumatoid arthritis (Asian). **(B)** Rheumatoid arthritis (European). **(C)** Coronary artery disease (CARDIoGRAM). **(D)** Coronary artery disease (C4D). **(E)** Schizophrenia. **(F)** Bipolar disorder.

The effect size estimation of GWAS data stratified with the derived allele frequency (DAF) could provide another perspective on polygenic architecture, which facilitates assessment based on population genetics (see Discussion). We classified all SNPs into five equally-sized DAF bins and estimated the effect-size distribution for each bin. For rheumatoid arthritis, the estimated distributions across the DAF bins were similar between Asian and European data (Figure 4). We observed peaks at positive effects, i.e., β > 0, for lower DAF bins, especially for DAF ≤ 0.2, and at negative effects for higher DAF bins, especially for DAF > 0.8. This indicates that low-frequency-derived and high-frequency-derived alleles are prone to act as risk and protective variants for disease occurrence, respectively. For coronary artery diseases, there was no substantial difference in the estimated effect-size distribution among DAF bins, compared with rheumatoid arthritis. For schizophrenia and bipolar disorder, we observed opposite tendencies: for schizophrenia, positive and negative effects were over-represented, especially at DAF < 0.2 and DAF > 0.8, respectively, whereas, for bipolar disorder, negative and positive effects were over-represented at DAF ≤ 0.2 and DAF > 0.8, respectively.

## Discussion

We have developed a simple and fast method for unbiased estimation of the proportion of disease-associated variants and the effect-size distribution based on the empirical Bayes estimation of SP-HMM. As we hypothesized in the introduction, we observed that the SP-HMM provided new insights in evaluating polygenic models of complex diseases: The SP-HMM can effectively distinguish various polygenic architectures, including the degree of polygenicity and distributions of genotype log-odds ratio, across diseases, and can also provide various perspectives of the polygenic architecture based on important variant categories such as DAF and eQTL. To demonstrate the SP-HMM, we selected four diseases with relatively large GWAS (more than several thousand samples) to apply the model, as representatives of various types of complex diseases, i.e., autoimmune diseases, cardiovascular diseases, and psychiatric disorders. We summarized the findings obtained from the application of the SP-HMM to each disease together with the current understanding of their respective genetic architectures in the literature and discuss the similarities and differences in the results across the diseases, and generalize these results to other complex diseases and discuss limitation of the method, accounting for eQTL/no-eQTL and DAF category.

Schizophrenia has long been suspected to be polygenic (Gottesman and Shields, 1972; Purcell et al., 2009). The estimated SNP heritability of the disease, 23–43%, suggests that common and weak-effect SNPs not reaching significance can explain the moderate to high degree of family-based heritability (Lee et al., 2012, 2013; Ripke et al., 2013; Golan et al., 2014; Loh et al., 2015; Palla and Dudbridge, 2015). An approximate Bayesian polygenic analysis (ABPA; Stahl et al., 2012) estimated that 8,300 independent SNPs contributed to the genetic basis of schizophrenia and that genotypic relative risks for schizophrenia were relatively small compared with the other complex diseases (Figure 3 in Ripke et al., 2013). Through a simulation-based method, schizophrenia has been shown to be extremely highly polygenic compared with the other diseases examined and to have more than 20,000 causal variants (Loh et al., 2015). The extremely high polygenicity has been also confirmed by the observation that local SNP heritability estimates in independent LD blocks for schizophrenia were the most ubiquitously distributed among six complex diseases (Shi et al., 2016). Here, using the SP-HMM, in schizophrenia π was estimated to be ~40% of disease-associated variants of independent SNPs in the genome (Table 1 and Table S5 in Supplementary Material). This suggests at least ~40,000 causal variants exist in the genome. The effect-sizes of the variants were clearly estimated to be very small for the most part, i.e., within |β| = 0.03, but larger than |β| = 0.05 for a small number of variants (Figure 2 and Figure S19 in Supplementary Material). The clear-cut visualization of the effect-sizes for schizophrenia is new finding, as it is for the three other diseases examined.

Bipolar has been estimated to have 25–47% SNP-heritability (Lee et al., 2013; Golan et al., 2014). Despite limited significant disease-associated variants identified (Sklar et al., 2011), the estimates show that common and weak-effect SNPs can explain moderate to high degree of family-based heritability. The SP-HMM estimated ~40% of independent SNPs in the genome to be disease-associated variants and the effect-sizes of the variants were estimated to be small for the most part, but ranging to around or more than |β| = 0.05 (Table 1 and Figure 2, and, Table S5 and Figure S19 in Supplementary Material).

For rheumatoid arthritis, the SNP heritability has been estimated to be relatively small, 13–18% (Stahl et al., 2012; Palla and Dudbridge, 2015). Rheumatoid arthritis has been identified as a disease for which the majority of the SNP heritability can be explained a small percentage of the genome (Shi et al., 2016). Our estimates of π, 3.6% for Asians and 8.1% for Europeans, were generally consistent with the previous estimates of 2.7% by ABPA (Stahl et al., 2012) and 5.4% (Palla and Dudbridge, 2015) for Europeans, and a significant portion of the estimate for *g* ranged up to a |β| = 0.05 and extended so far as 0.1 (Table 1 and Figure 2). In the rheumatoid arthritis stratification analysis based on eQTLs, we observed a high enrichment of per-SNP variance due to eQTLs determined by peripheral blood samples (Table S6 in Supplementary Material), similar to the enrichment on per-SNP variance by blood-specific DNaseI hypersensitivity sites (DHS) (Gusev et al., 2014), which were also strongly associated with expression variation (Degner et al., 2012). As peripheral blood samples include multiple types of leukocytes, the eQTLs have the potential to control immune-related gene expressions that are associated with the occurrence of rheumatoid arthritis. Although eQTLs were defined using European samples (Westra et al., 2013), the enrichment of 10.7-fold in the Asian population was larger than the 5.7-fold enrichment observed in the European population. The same tendency has been observed for the validated 100 non-MHC SNPs (Extended Data Figure 5 in Okada et al., 2014). This might be explained by non-eQTL-SNPs with large effects, such as non-synonymous SNPs in genes PTPN22 (R620W) and TYK2 (P1104A), which exist in Europeans but are absent or exist to a lesser degree in Asian populations. Some eQTL-SNPs were estimated to have large effect size |β| > 0.05 (Figure 3) in rheumatoid arthritis.

For coronary artery disease, the SNP heritability has been estimated to be 33–48% (Stahl et al., 2012; Golan et al., 2014). The SP-HMM estimated that π for C4D was larger (estimates ~26% in the both of *P*-value-based and randomly-pruned sets) than that for CARDIoGRAM (estimates of 15.9 % and 23.5 % in the *P*-value-based and randomly-pruned sets, respectively). Since SNPs of C4D were pruned by using LD structure of European ancestry (see section Materials and Methods), LD remaining in Asian SNPs, possibly linked with one causal variant, might increase the estimated proportion of disease-associated SNPs.

Regarding the similarity and differences among the four diseases, we identified a common feature across the four complex diseases for which the genetic basis consists of enormous variants (more than several thousand independent risk variants; Table 1 and Table S5 in Supplementary Material) with very small effects (majority of genotypic OR for risk alleles are within 1.05; Figure 2 and Figure S19 in Supplementary Material). The recently proposed “omnigenic” model hypothesizes that “gene regulatory networks are sufficiently interconnected such that all genes expressed in disease-relevant cells are liable to affect the functions of core disease-related genes and that most heritability can be explained by effects on genes outside core pathways” (Boyle et al., 2017). This model explains the existence of such numerous risk-variants with small effects. Next, the SPHMM showed that polygenicity was estimated to widely vary among the four diseases, e.g., schizophrenia was extremely polygenic (~40% of independent genome-wide SNPs were risk variants, most within odds ratio OR = 1.03), whereas rheumatoid arthritis was less polygenic (~4 to 8% risk variants, with a significant portion reaching OR between 1.05 and 1.1). The fewer but relatively large-effect variants would be the reason why the number of GWAS hits for rheumatoid arthritis (~100 significant SNPs) is comparable to that for schizophrenia despite of small SNP heritability compared with schizophrenia. In fact, the effect sizes of validated variants for rheumatoid arthritis were generally larger than those for schizophrenia (Okada et al., 2014; Ripke et al., 2014). Our estimate of *g* means that the effect sizes of variants that will be detected in future should also be large for rheumatoid arthritis relative to other complex diseases.

Using DAF-stratified analysis for rheumatoid arthritis, we estimated more risk/protective derived alleles in low/high DAF (Figure 4). Simple models based on a theory of population genetics for DAF (Sawyer and Hartl, 1992) (see Figure S20 in Supplementary Material) could help interpret results from the DAF analysis, and thus provide another perspective on the differences among diseases (see Supplementary Note in Supplementary Material for details). Among such models, the “deleterious-risk and advantageous-protective mutation” model with weak selection was best fitted for rheumatoid arthritis (Figure S21 in Supplementary Material). Because most of the risk genes for rheumatoid arthritis are implicated in immune system regulation (Okada et al., 2014), these low- and high-derived alleles would tend to skew an individual's immune function toward either deleterious or beneficial directions. Meanwhile, this skewing may result in breaking the balance between immunity and tolerance, leading to rheumatoid arthritis.

Although some authors have reported that bipolar disorder and schizophrenia share a large amount of genetic factors (Purcell et al., 2009; Lee et al., 2013), we observed opposite tendencies in the genetic architecture for these diseases: risk (protective) and protective (risk) derived alleles were over-represented, especially at DAF ≤ 0.2 and DAF > 0.8 for schizophrenia (bipolar disorder) (Figure 4). This paradoxical result was consistent with a previous report that, among low minor allele frequency (1–5%) SNPs, the R/P ratio (ratio of the number of detected variants with risk in minor allele to those with protective effect) for schizophrenia was significantly larger than one, while for bipolar disorder it was less than one (see Table 1 in Chan et al., 2014). Again, applying the same population genetics models, it was found that both the “deleterious-risk and advantageous-protective mutation” and “deleterious-risk mutation” models were better fitted for schizophrenia, whereas the “advantageous-risk and deleterious-protective mutation” model was the best fitted for bipolar disorder (Figure S21 in Supplementary Material). Recently, genetic correlations between creativity and both schizophrenia and bipolar disorder were reported, but they were much stronger for bipolar disorder (Keller and Visscher, 2015; Power et al., 2015). There is possibly some relationship between creativity and over-represented (positively selected) risk mutations at high DAF in bipolar since creativity is an important aspect for humans. In this way, the SP-HMM might provide a clue for resolving the shared and specific genetic etiologies between the two genetically related diseases.

The SP-HMM can also provide posterior effect-size estimates of individual SNPs based on the estimated genetic architecture, $\hat{\pi }$ and *ĝ* (Stephens and Balding, 2009; Matsui and Noma, 2011b). To evaluate individual eQTL-SNPs, we used the estimated genetic architecture as the prior and listed the top SNPs with larger posterior means of effect size, |β| > 0.05 (Data Sheet 1 in Supplementary Material). As this list includes eQTLs such as RNASET2 and ADO, which have not been previously linked to rheumatoid arthritis (Okada et al., 2014), this approach might be effective for identifying disease associated eQTL-SNPs. For the other diseases, enrichments of per-SNP variance due to the eQTLs in peripheral blood cells were also observed. Since the eQTL-SNPs are associated with immune-related gene expression, these observations were consistent with the fact that coronary artery disease is a chronic inflammatory disorder and previous reports of the genetic overlap between immune diseases and schizophrenia (Stringer et al., 2014). However, it should be noted that precise estimation of the eQTL effects in these diseases requires additional eQTL data covering all the tissues and cells related to the diseases.

Although we only examined four complex diseases so far, the feature of enormous risk variants with very small effect could be generalized to almost all other complex diseases based on our experience analyzing several other diseases. It should be noted that polygenicity should generally differ among complex diseases even among those that belong to the same categories, i.e., psychiatric disorders. Specifically, whether the GWAS of a particular disease with a realizable sample size would successfully detect disease-associated variants largely depends on the existence of variants with relatively large effects, e.g., genotypic odds ratio >1.05, or >1.10. The number of such variants would vary largely between complex diseases.

The limitation of our method is that SP-HMM evaluates π and *g* with respect to the marginal effects of SNPs rather than with respect to the effects of underlying causal variants themselves. Nevertheless, the results of the SP-HMM estimation reflect the effects of the causal variants themselves through linkage disequilibrium.

Lastly, the SP-HMM and empirical Bayes method, which can provide fine characterization of genetic architecture, can also contribute to accurate power analysis of GWAS (Park et al., 2010; Ripke et al., 2013) and estimation of the predictive capability of disease risk (Chatterjee et al., 2013). The SP-HMM can also be extended to multi-dimensional settings, e.g., for quantification of sex in the genetic architecture of a disease, or the (antagonistic) pleiotropic genetic architecture in multiple diseases. This kind of multi-dimensional analysis would be novel and could provide new perspectives on multi-dimensional genetic effects, e.g., through a two-dimensional visualization of effect-size distributions for schizophrenia and bipolar diseases. Such analyses will be applied in future reports.

## Materials and methods

### Semi-parametric hierarchical mixture model (SP-HMM)

We defined the effect size, β_*j*_, for the *j*-th SNP of the total *m* SNPs as the genotype log-odds ratio under the additive allele dosage model. We considered the dosage of “derived mutant” alleles. Namely, the genotypes *AA*, *Aa*, and *aa* in each SNP had dosages *x*_*j*_ = 0, 1, and 2, respectively, where *a* was the derived and *A* was the ancestral allele. $Y_{j}={\hat{\beta }}_{j}$ was an estimate of log-odds ratio for the *j*-th SNP (e.g., the standard maximum likelihood estimate). For each *Y*_*j*_, we assumed a mixture structure with two components, null and non-null SNPs, in terms of association with disease susceptibility. To be specific,

(1) $$f_{j}\left(y_{j}\right)=(1-\pi )f_{0j}\left(y_{j}\right)\;+\pi f_{1j}\left(y_{j}\right),$$

where *f*_0*j*_ and *f*_1*j*_ are the probability densities for null and non-null SNPs, respectively, and π is the prior probability of being non-null. For null SNPs, we specified $y_{j}\;\sim {\;f}_{0j}\left(y_{j}\right)=N\left(0,{\hat{V}}_{{\hat{\beta }}_{j}}\right)$ based on the asymptotic distribution of ${\hat{\beta }}_{j}$, where ${\hat{V}}_{{\hat{\beta }}_{j}}$ is an empirical variance estimate of ${\hat{\beta }}_{j}$ (e.g., the standard Wald-type variance estimate for ${\hat{\beta }}_{j}$). For non-null SNPs, we assumed the hierarchical structure: $y_{j}|\beta_{j}\;\sim \;f_{1j}\left(y_{j}|\beta_{j}\right)=N\left(\beta_{j},{\hat{V}}_{{\hat{\beta }}_{j}}\right)$ and β_*j*_ ~ *g*, where the prior effect-size distribution *g* was unspecified. In this model, the standard asymptotic normality was assumed for ${\hat{\beta }}_{j}$ at the individual SNP level, while its true effect size β_*j*_ followed a non-parametric prior distribution *g*, forming a semi-parametric hierarchical mixture model (SP-HMM) (Matsui and Noma, 2011a,b). The assumption that each *y*_*j*_ is mutually independent would be reasonable for a set of LD-pruned SNPs.

### Empirical bayes estimation

We estimated the priors, π and *g*, in the SP-HMM based on the data by applying an expectation–maximization (EM) algorithm, called the smoothing-and-roughening algorithm (Shen and Louis, 1999), to incorporate the non-parametric prior distribution *g*(Matsui and Noma, 2011a,b). The non-parametric estimate of *g* was supported by fixed discrete mass points ***p*** = (*p*_1_*, p*_2_*, …, p*_*B*_) at a series of nonzero points ***b*** = (*b*_1_*, b*_2_*, …, b*_*B*_) (*b*_1_ < *b*_2_ < ··· < *b*_*B*)_. We specified a wide range for the mass points, such as *b*_1_ = −0.3 to *b*_*B*_ = 0.3 (0.74 to 1.35 in odds ratio), to support the effect-size distributions in many complex diseases. We set the number grid points as 120, such that ***b*** = (−0.300, −0.295, …, −0.005, 0.005, …, 0.295, 0.300). The initial value of π, π_*init*_, and the initial distribution of *g*, *g*_*init*_, were determined sequentially. Setting *g* to be uniformly distributed (i.e., *p*_*i*_ = 1/*B* for all *i*), the EM procedures for candidate initial values, π = 0.1, 0.2, …, or 0.9, were run 200 times and the value of estimated π with maximum likelihood was selected as π_*init*_. Then setting *g* to be uniformly distributed again, we got *g*_*init*_ by the EM procedure with fixed π = π_*init*_(the EM iterations were stopped when the relative change of π in one iteration was < 0.005% or after 200 iterations). The final EM procedure set *g* = *g*_*init*_ and π = π_*init*_, and was stopped when the relative changes in the estimate of π in one iteration was < 0.005 % or 2000 iterations was reached. We applied a parametric bootstrap method based on the estimated SP-HMM to estimate standard errors of the estimate for π.

### Liability-scale variance explained by SNPs

As shown by So et al. (2011a), the log odds ratio, β_*j*_, together with the allele frequency and the disease prevalence, can be transformed to the variance explained by the *j*-th SNP, denoted as *v*_*j*_, in the liability threshold model. In the liability threshold model, we assumed that an underlying liability to disease follows a normal distribution and individuals that exceeded a threshold of liability, *T*, were affected with the disease. Individuals with the genotypes of *AA, Aa*, and *aa* at the *j*-th locus had liability distributions with different means, but the same residual variance. We let *p*_*j*_ be the derived allele frequency and *h*_*j*,*x*_*j*__ be the frequency of genotype *x*_*j*_ (*x*_*j*_ = 0, 1, 2) in the general population. Assuming Hardy-Weinberg equilibrium in the population, the genotype frequencies are given by $h_{j,0}={\left(1-p_{j}\right)}^{2},\;h_{j,1}=2p_{j}\left(1-p_{j}\right),\;and\;h_{j,2}={p_{j}}^{2}$. Using the overall mean liability, μ_*all*_, and the mean liabilities of genotype *x*_*j*_, μ_*j*,*x*_*j*__, the variance explained by *j*-th SNP is given by

(2) $$v_{j}{}^{*}=\sum_{x_{j}=0}^{2}h_{j,x_{j}}\;{\left(\mu_{j,x_{j}}-\mu_{all}\right)}^{2}.$$

For evaluating μ_*j*,*x*_*j*__, we used the penetrance of genotype *x*_*j*_, denoted by $\varphi_{j,x_{j}}=1/\left(1+e^{-\alpha_{j}-\beta_{j}x_{j}}\right)$ under the additive allele dosage model, where α_*j*_ was determined under the constraint involving the disease prevalence *K*, $K=\overset{2}{\underset{x_{j}=0}{\sum }}h_{j,x_{j}}\;\varphi_{{j,x}_{j}}$. Assuming that the residual variance of each genotype was 1, the mean liability of each genotype was given by

(3) $$\Phi^{-1}\left(1-\varphi_{j,x_{j}}\right)=T-\mu_{j,x_{j}}\text{ for }{\text{x}}_{\text{j}}=\text{0},\text{1},\text{ and 2},$$

from which we obtained values of μ_*j*,*x*_*j*__, where Φ was the cumulative distribution function of the standard normal distribution. Of note, one of the mean liabilities of genotypes can be set as an arbitrary value, as it does not affect the variance estimate. Finally, *v*_*j*_ was obtained by $v_{j}={v_{j}}^{*}/\left(1+{v_{j}}^{*}\right)$. This corresponded to the variance under the standard liability threshold model with the unit total variance of liability, as is assumed in heritability estimation (Falconer, 1965; Lee et al., 2011).

We estimated the distribution of *v*_*j*_ for non-null effects using the estimated effect-size distribution *ĝ*, together with using allele frequencies and the prevalences. The allele frequencies were retrieved from the 1000 Genomes Project Phase 3 (The 1000 Genomes Project Consortium., 2015) and the prevalences previously assumed in estimating SNP heritability were used (Stahl et al., 2012; Golan et al., 2014). Then, the point estimate of *v*_*j*_, ${\hat{v}}_{j}$, was gained as the product of the estimate $\hat{\pi }$ and the mean of the estimated distribution of *v*_*j*_ for non-null effects. The total liability-scale variance, *V*, explained by the pruned SNP sets, was then estimated as a simple sum of ${\hat{v}}_{j}$ over all SNPs in the sets.

### GWAS data analysis

The six sets of GWAS summary statistics that we used were available online. The characteristics of individual GWASs are shown in Tables S1, S2 in Supplementary Material. For rheumatoid arthritis, the MHC region (chromosome 6, 25–35 Mb) was removed. The derived/ancestral states of alleles were determined by using dbSNP.

We used two kinds of pruned SNP sets, *P*-value-based and random-pruned sets, in the non-stratified SP-HMM analysis (Table 1 and Figure 2). To gain the *P*-value-based pruned set for a GWAS, we began by selecting the most strongly associated SNP, i.e., the SNP with the lowest P value, in a reference GWAS as a SNP of the pruned set, and all other SNPs in LD (*r*^2^ > 0.1) with the selected SNP were removed. The process was repeated until no SNPs remained. LD information was retrieved from the HapMap data base (HapMap phases I+II+III, release 27) (International HapMap 3 Consortium, 2010). In selecting SNPs with strong associations for Asian rheumatoid arthritis GWAS, European rheumatoid arthritis GWAS data were used as a reference for association, and vice versa. For coronary artery disease, the data of two GWAS, CARDIoGRAM and C4D, were used reciprocally. For the two genetically correlated diseases, schizophrenia and bipolar disease, the data of two GWAS for the two diseases were used reciprocally. For the random-pruned sets, we included SNPs randomly, irrespective of degrees of association, i.e., *P*-values in the reference GWAS data, such that no SNPs in the set were in *r*^2^ > 0.1.

For stratified analysis by eQTL/non-eQTL-SNPs, we defined an “eQTL SNP” as a cis-eQTL SNP detected with false discovery rate < 0.5 using peripheral blood samples (Westra et al., 2013). In the eQTL/non-eQTL-SNPs set analyzed, all the eQTL and non-eQTL SNPs were selected to be nearly independent of one another (*r*^2^ ≤ 0.1). In this data set, eQTL SNPs showing stronger associations (i.e., lower *P*-values) with gene expressions were preferentially included, and LD pruning was conducted as in the *P*-value-based pruned sets. Non-eQTL SNPs were randomly selected.

In the DAF-stratified analysis, the allele frequencies of SNPs were determined by the 1000 Genome phase III data (The 1000 Genomes Project Consortium, 2015). For each DAF bin, we used 100,000 SNPs randomly selected from GWAS SNPs regardless of LD. This was because estimates of SP-HMM were unstable due to the small number of SNPs (e.g., a few thousand SNPs) when LD pruned sets were used. Note that, in C4D GWAS, the number of SNPs used in 0.4 < DAF ≤ 0.6, 0.6 < DAF ≤ 0.8, and 0.8 < DAF were 94506, 70170, and 49116, respectively, since the SNPs of C4D GWAS was limited (Table S3 in Supplementary Material). The obtained results (i.e., estimates of π and *g*) using the pruned sets (data not shown) were close to those sampled regardless of LD, and both results had the same trends over DAF bins.

For selecting high quality SNPs and LD information in the above section, HapMap data of Japanese individuals in Tokyo (JPT) and European-ancestry individuals from Utah (CEU) were used for Asian rheumatoid arthritis GWAS data and the other GWAS data, respectively. Similarly, for information of allele frequencies, East Asian and European 1000 Genome Project data were used for Asian rheumatoid arthritis GWAS data and the other GWAS data, respectively.

## Source code availability

The R code implementing the SP-HMM analysis used in this study is freely available through GitHub (https://github.com/jonishino/SP-HMM).

## URLs

HapMap 3, ftp://ftp.ncbi.nlm.nih.gov/hapmap/frequencies/2010-05_phaseIII; 1000 Genome, ftp://ftp-trace.ncbi.nih.gov/1000genomes/ftp/release/20130502; dbSNP (Build 141), http://www.ncbi.nlm.nih.gov/SNP; eQTL in blood, http://genenetwork.nl/bloodeqtlbrowser/2012-12-21-CisAssociationsProbeLevelFDR0.5.zip; rheumatoid arthritis summary statistics, http://plaza.umin.ac.jp/~yokada/datasource/software.htm; schizophrenia and bipolar disorder summary statistics, www.med.unc.edu/pgc/downloads; coronary artery disease summary statistics, http://www.cardiogramplusc4d.org/.

## Author contributions

JN: developed the methods, performed the analyses, and wrote the manuscript. YK: provided essential ideas and interpretations for the study direction and results. DS and TM: contributed to the data acquisition and the analyses. HN: provided the initial version of script for SP-HMM analysis. YK, MK, HO, KB, and TT: improved the manuscript. TT: directed and supervised the study; SM: conceived the study idea, developed the methods, wrote the manuscript, and, directed the study. All authors contributed the final manuscript.

### Conflict of interest statement

The authors declare that the research was conducted in the absence of any commercial or financial relationships that could be construed as a potential conflict of interest.
