# Supplementary material for: Empirical Bayes Estimation of Semi-parametric Hierarchical Mixture Models for Unbiased Characterization of Polygenic Disease Architectures
Source: Front Genet. 2018 Apr 24;9:115. doi: 10.3389/fgene.2018.00115 (PMC5928254; doi:10.3389/fgene.2018.00115)
Supplement: Supplementary file 1 [file Presentation_1.pdf]

# *Supplementary Material*:

Empirical Bayes estimation of

semi-parametric hierarchical mixture models

for unbiased characterization of polygenic disease architectures

Jo Nishino, Yuta Kochi, Daichi Shigemizu, Mamoru Kato, Katsunori Ikari,

Hidenori Ochi, Hisashi Noma, Kota Matsui, Takashi Morizono, Keith A Boroevich,

Tatsuhiko Tsunoda & Shigeyuki Matsui

**Correspondence:**
Shigeyuki Matsui
smatsui@med.nagoya-u.ac.jp

# Supplemental Note

## 1. Evaluation of the SP-HMM estimation by simulation

### 1.1. Method

#### 1.1.1. Main Simulation

**General settings**

We evaluated the performance of our method using the SP-HMM to estimate the proportion of disease-associated SNPs, $\pi$, and the effect-size distribution, $g$, through Monte Carlo simulations under realistic situations in GWAS. We considered a case-control study where $n$ = 3000, 5000, 7000, 10000, 30000, or 100000 cases and controls, such that $2n$ = 6000, 10000, 14000, 20000, 60000, or 200000 in total, were sampled from a general population with disease prevalence $K$ = 0.01. We set the number of SNPs as $m$ = 20000, 50000, and 100000. We considered the 'less', 'moderate' and 'highly polygenic' settings to cover a wide range of polygenicity, in which the proportion of disease-associated SNPs, $\pi$, was set to 3%, 10%, and 30%, respectively.

In generating genotype counts for SNPs with the effect size $\beta$ in the case and control samples, the Hardy-Weinberg equilibrium for genotype frequencies in the general population and linkage equilibrium among SNPs (i.e., independence of alleles) were assumed. The probability of genotype $x_j$ for the $j$-th SNP in the case group is expressed as $P(x_j|case) = h_{j,x_j}\varphi_{j,x_j}/K$, where $\varphi_{j,x_j}$ is the penetrance of $x_j$ and $h_{j,x_j}$ is the frequency of $x_j$ in the general population, satisfying $h_{j,0} = (1-p_j)^2$, $h_{j,1} = 2p_j(1-p_j)$, and $h_{j,2} = {p_j}^2$ for the derived allele frequency $p_j$. Similarly, the probability of genotype $x_j$ in the control group is given by $P(x_j|control) = h_{j,x_j}(1-\varphi_{x_j})/(1-K)$. Here, in the penetrance, $\varphi_{j,x_j} = 1/(1+e^{-\alpha_j-\beta_j x_j})$, $\alpha_j$ was determined under the constraint, $K = \sum_{x_j=0}^{2} h_{j,x_j}\,\varphi_{j,x_j}$, for a given $\beta_j$ that follows the effect-size distribution $g$. The genotype counts in the case group were generated by the multinomial distribution with the sample size $n$ and the probabilities $P(x_j|case)$ for $j$ = 1,

2, and 3. The genotype counts in the control sample were similarly generated.

**Simulations using normal distributions**

For null SNPs without association to disease, we set the effect size $\beta_j$ to be 0. For the effect-size distribution $g$ for non-null SNPs, to generate $\beta'$s, we used normal distributions in two ways (Table S1a). First, we used simple normal distributions with expectation 0 and standard deviation of 0.03 for the less and moderate polygenic settings, and 0.015 for the highly polygenic settings, respectively. Second, as representative examples of asymmetric effect-size distribution, for the less and moderate polygenic settings, we generated $\beta'$s from mixture distributions of negative truncated normal distribution with standard deviation of 0.03 and positive truncated normal distribution with standard deviation of 0.04. For the highly polygenic settings, we used negative truncated normal distribution with standard deviation of 0.015 and positive truncated normal distribution with standard deviation of 0.02. The mixing proportions of positive and negative distributions was determined so that the whole (mixed) effect-size distributions, *g,* are continuous functions.

The derived allele frequencies, DAF's, were generated from a uniform distribution $U(0.01, 0.99)$. In the DAF-stratified analysis, DAF's were generated from $U(0.01, 0.2)$, $U(0.2, 0.4)$, and $U(0.4, 0.6)$. Note that the results for SNP sets with DAFs generated from $U(0.6, 0.8)$ and $U(0.8, 0.99)$ should be the same as for SNP sets with DAFs generated from $U(0.2, 0.4)$ and $U(0.01, 0.2)$, respectively, since each two corresponding DAF bins have the same ranges of minor allele frequency, which is determinant of an empirical variance estimate of $\hat{\beta}_j$, $\hat{V}_{\hat{\beta}_j}$.

**Simulations using gamma distributions**

Although it is natural to think that effects for no-null SNPs are centered around smaller size values, as modeled by normal distributions with expectation 0, the possibility that most effects

for no-null SNPs are apart from 0 cannot be completely excluded. Thus, we also conducted the simulations using gamma distributions (Table S1b). We generated positive effects, $\beta'$s, from gamma distributions with expected value and standard deviation of (0.03, 0.015), (0.03, 0.015), and (0.018, 0.005) for the less, moderate, and highly polygenic settings, respectively. We specified the same distributional forms for negative effects, but we set the ratio of positive to negative effects as 2:1. We also considered other forms of the effect-size distribution. For the less or moderate polygenic settings with large effects, we generated positive effects according to a mixture distribution with two components: a gamma distribution with expected value of 0.03 and standard deviation of 0.015, and a fixed very large effect, $\beta$ = 0.1, with mixing proportions of 95% and 5%, respectively. Similarly, in the highly polygenic with large effects setting, we generated positive effects according to a mixture model with two components: a gamma distribution with expected value of 0.018 and standard deviation of 0.005, and a fixed very large effect, $\beta$ = 0.08, with mixing proportions of 98% and 2%, respectively. The numbers of SNPs were set to $m$ = 100000, and, DAF's were generated from a uniform distribution $U$(0.01, 0.99).

**Evaluation of estimation**

From the simulated genotype data, the set of summary statistics $\hat{\beta}_j$ and $\hat{V}_{\hat{\beta}_j}$ for $m$ SNPs was obtained by a univariate logistic regression analysis. We conducted 100 simulation repetitions for each configuration. For each simulated dataset, we adopted the estimation method for $\pi$ and $g$ using the SP-HMM. Boxplots were used for assessing estimation accuracy and precision for $\pi$. Accuracy of estimating $g$ was confirmed by average of estimated effect-size distribution across simulations. With respect to estimation of $\pi$, for comparison with our method, we also showed the results obtained by 'qvalue' R package (Bioconductor v.3.4) with the default setting. For $g$, simple estimates of log-odds ratio, $\hat{\beta}$, with/without some qvalue threshold calculated by

the R package were shown. Boxplots of estimates of the liability-scale variance, $V$, were also shown.

### 1.1.2. Simulations using estimated genetic architecture from real data

In addition to the above artificial simulations, we conducted simulations using $\beta's$ based on the real data, i.e., the estimates of $\pi$ and $g$ by the SP-HMM from the P-value-based SNP sets (Table 1 and Figure 2), the eQTL/non-eQTL SNP sets and the SNP sets stratified by DAF. Specifically, the numbers of disease-associated SNPs were determined as $\hat{\pi} \times m$ (fixed). For the effect-size distribution of the associated SNPs, the numbers of SNPs belonging to $i$-th mass point, $b_i$, were determined as $\hat{p}_i \times m$ (the product of the estimated proportion of SNPs belonging to $b_i$ and the number of SNPs; fixed), where decimals were rounded to the nearest integer. DAF's were generated from $U(0.01, 0.99)$ for the P-value-based SNP sets and the eQTL/non-eQTL SNP sets, and from $U(0.01, 0.2)$, $U(0.2, 0.4)$, $U(0.4, 0.6)$, $U(0.6, 0.8)$, and $U(0.8, 0.99)$ for the SNPs belonging to each DAF bin for the SNP sets stratified by DAF. The numbers of SNPs, $m$, were set to be the same values as those of the real GWAS data. Boxplots were used for assessing estimation accuracy and precision for $\hat{\pi} \times m$. Accuracy and precision of estimating the effect-size distribution was confirmed by the average of estimated effect-size distribution across simulations and by showing individual simulation results.

### 1.1.3. Simulations for the case with weak LD

The SP-HMM estimates the proportion of disease-associated SNPs and effect-size distribution under the assumption of independence among SNPs. However, even stringent LD-pruned SNPs based on $r^2$ >0.1 could be in weak linkage disequilibrium (LD) with one another. We conducted simulations to investigate how much influence this weak LD would have on the SP-HMM estimation as follows. We sampled 1000 consecutive SNPs from the top of chromosome 1 in the

P-value pruned SNPs (chromosome 1: 0.8–22 Mb) for schizophrenia GWAS data. For the 1000 SNPs, we obtained linkage disequilibrium, $r_{(1000G)}$, between SNP pairs of all 20 consecutive SNPs, which was calculated as correlations of derived allele count (0, 1, and 2) of 379 CEU individuals from the 1000 Genomes project phase 1 integrated release version 3[1] using the -r --ld-window 21 option in PLINK[2]. LD between distant SNP pairs apart from each other more than 20 SNPs were neglected (set to $r_{(1000G)} = 0$). The covariance (equaling to correlation due to normalization) between the normalized log-odds ratios in a sample, $\hat{\beta}/\sqrt{\hat{V}_{\hat{\beta}}}$, for the $j$-th and $k$-th SNPs is approximately equal to the sample LD, $r_{jk}$, between the two SNPs[3]. The values of $r_{jk}$ could not be obtained from each GWAS data. Thus, substituting $r_{(1000G)jk}$ for $r_{jk}$, we generate $\hat{\beta}'$s for the 1000 SNPs using the multivariate normal distribution: $\hat{\boldsymbol{\beta}} \sim N(\boldsymbol{\beta}, \boldsymbol{\Sigma})$, where the ($j$, $k$) element of $\boldsymbol{\Sigma}$ is $\sqrt{\hat{V}_{\hat{\beta}_j}}\sqrt{\hat{V}_{\hat{\beta}_k}} r_{jk}$. We tested the case of more strong LD where $r_{jk}$ is replaced $1.5 \times r_{(1000G)jk}$ (If $1.5 \times r_{(1000G)jk}$>1 or < -1, $r_{jk}$ is replaced by 1 or -1, respectively). The true log-odds ratios, $\beta'$s, were generated using normal distributions in the same way as described above (see also Table S1a). The process generating $\hat{\beta}'$s using $\hat{\boldsymbol{\beta}} \sim N(\boldsymbol{\beta}, \boldsymbol{\Sigma})$ for the 1000 SNPs was repeated 1000 times and we got $\beta'$s for the hypothetical 100000 pruned SNPs. Estimation performances for $\pi$, $g$, and $V$ were assessed in the same manner as those for 'Main simulations' (see section 1.1.1).

With respect to the SNPs used in the DAF-stratified analysis, which are not pruned but randomly sampled from all SNPs, using the SNP set with $0.4 < \mathrm{DAF} \leq 0.6$ for schizophrenia GWAS, we conducted simulations to investigate how much influence the LD had on the SP-HMM in the same way as the P-value pruned SNPs. In addition, the simplified case where $r_{jk} = \sqrt{0.03}$ for all pairs of SNPs (uniform correlation structure for genotypes), meaning weak but long range LD, was investigated.

## 1.2. Results

### 1.2.1. Main simulations

**Simulations using normal distributions**

Figures S1 and S3 show the simulation results for the estimates of $\pi$ and $g$, respectively, in the settings using normal distributions for effect-size distributions, with DAFs ranging from 0.01 to 0.99. The results for the moderate polygenic setting with symmetric effect sizes using $m$=100,000 were also provided in Figure 1 in the main body. We also provided a summary of these results in Table S2. When the numbers of SNPs ($m$) were ≥50000 and the case and control sample sizes ($n$) were ≥7000 (i.e., ≥14000 in total), our estimation method generally yielded nearly unbiased estimates for $\pi$ in the less, moderate, and highly polygenic settings (Figure S1). In these cases, our method also captured well the range and the forms of the true effect size distributions (Figure S3). For the real P-value-based and random-pruned SNP sets, the estimates of $\pi$ and $g$ by the SP-HMM should be reliable since there are about 80000～100000 SNPs and sufficient sample sizes (>16000 total sample size in case and control; Table S3). For the real non-eQTL SNP sets with about 50000～75000 SNPs, estimates of $\pi$ and $g$ by the SP-HMM should be reliable, with the exception that the estimate of $\pi$ for Asian rheumatoid arthritis, 1.1%, is out of the range of simulated values.

Even under relatively small sample sizes, $n$ = 3000 or 5000, the estimates of $\pi$ and $g$ were still unbiased for the less or moderate polygenic settings. However, for the highly polygenic settings, $\pi$ was underestimated and the estimate of $g$ was biased with higher frequency for large effects under small sample size situations, $n$ = 3000 or 5000 (Figures S1 and S3).

In cases with small numbers of SNPs, $m$ = 20000 (this is approximately corresponding to that of eQTL SNP sets), $\pi$ was overestimated and the estimate of $g$ was biased with higher frequency for small effects under the less polygenic settings (the overestimation of $\pi$ should

relate to estimation bias of $g$ with higher frequency for small effects). On the other hand, under moderate or highly polygenic settings, even in the case of $m$ = 20000, the method provided good estimates for $\pi$ and $g$ under $n \geq 7000$. In the real eQTL SNP sets, the smallest estimate of $\pi$ wase 18.4% in Asian rheumatoid arthritis GWAS, suggesting that even with more than the 'moderate' degree of polygenicity, the SP-HMM should be reliable.

For the liability-scale variance, $V$, almost unbiased estimates could be obtained irrespective of the numbers of SNPs and the sample size in all the cases investigated (Figure S2)

Figures S4 and S5 show the simulation results for the estimates of $\pi$ and $g$, respectively, in the DAF stratified analysis. For DAF = 0.2-0.4 and 0.4-0.6, the results were similar to those for non-stratified analysis. For DAF = 0.01-0.2, $\pi$ tended to be underestimated and large effects in $g$ tended to be overestimated in the relatively small sample size. Particularly when $n$ = 3000 in the less and moderate polygenic settings, or, when $n \leq 7000$ in the highly polygenic settings, the SP-HMM underestimated $\pi$. In the real analysis, the estimation of $\pi$ using SNPs with DAF = 0.01-0.2 for bipolar GWAS might likely be underestimated due to its sample size and its high polygenicity. The other results for DAF stratified analysis should not suffer from serious bias.

The qvalue method almost consistently underestimated $\pi$ (Figures S1 and S4). Exceptionally, in DAF = 0.4-0.6 and $n \geq 30000$ (in the case $\hat{\beta}$ should have the smallest sampling variance), $\pi$ were unbiasedly estimated by the qvalue method. We also confirmed that the true effect-size distribution, $g$, cannot be estimated by simple estimates of log-odds ratio, $\hat{\beta}$, even with some qvalue threshold (Figure S6).

**Simulations using gamma distributions**

Figures S7 and S9 show the simulation results for the estimates of $\pi$ and $g$, respectively, in the settings using gamma distributions for effect-size distributions. Estimates of $\pi$ were almost unbiased in $n$ = 100000 (Figure S7). Whereas, in the smaller sample sizes, especially in the moderate sample size ($n$=5000-1000), the SP-HMM tended to overestimate $\pi$. In particular, in the highly polygenic setting $\pi$ was estimated as 0.4 on average for true $\pi = 0.3$ in $n$ = 10000. This overestimation for $\pi$ should be connected to the overestimation of effects near 0 in $g$ (Figure S9). In reality, it is more natural that there are assumed to be more SNPs around zero effects in $g$, as are described in the settings using normal distribution. Note that even in the settings using gamma distribution, the SP-HMM detected the effect-size range and approximate form of true $g$. It was also remarkable that our method could properly estimate asymmetric effect-size distributions, possibly with a small peak for large effects (Figure S9). In the same way as the settings using normal distributions, the SP-HMM estimated the liability-scale variance, $V$, almost unbiasedly, irrespective of the numbers of SNPs and the sample size in all the cases investigated (Figure S8).

### 1.2.2. Simulations using estimated genetic architecture from real data

In the results of simulations using estimated genetic architecture from real data, the assigned number of associated SNPs and distribution of effect sizes are generally well reconstructed by the SP-HMM (Figures S10-15). For the estimates of effect size distribution, the SP-HMM captured the assigned effect size distribution flexibly. These results are consistent with those of the polygenic architectures of complex diseases using the GWAS summary data, which are sufficiently well estimated by the SP-HMM. Note that the estimates of effect size distribution for rheumatoid arthritis GWAS, particularly for the non-eQTL SNP set, tended to vary from simulation to simulation compared with those of other GWAS because of relatively small

sample size.

### 1.2.3. Simulations for the case with weak LD

Figures S16 and S17 show the simulation results for the estimates of $\pi$ and $g$, respectively, taking account for weak LD among SNPs. For the cases using LD from the 1000 Genomes project ('Real', 'Real×1.5', 'Real (DAF3)' and 'Real (DAF3)×1.5' in the figures), the $\pi$ and $g$ of the SP-HMM yielded nearly unbiased estimates for $\pi$, with little underestimation in the highly polygenic settings. The SP-HMM also captured well the range and the forms of the true effect size distributions, with little overestimation in the less polygenic settings. For the case of weak but long range LD ('$r$ = $0.03^{1/2}$' in the figures), the variations of estimates of $\pi$ were large and $\pi$ was clearly overestimated in the less or moderate polygenic settings. In that case, the estimate of $g$ was not clearly biased with higher frequency for small effects. Based on the above observations, although LD indeed did affect the SP-HMM estimation, week LD existing in the used SNPs in the study should have little impact on the estimation.

## 2. Exploring natural selection

We explored the relationship between variants affecting complex diseases and natural selection using the random-pruned sets for each GWAS. Under the Wright-Fisher model with $N$ diploids and irreversible mutation, the fitness of the genotypes $AA$, $Aa$, and $aa$ in a locus were assumed to be 1, 1+$s$, and 1+2$s$, respectively. The stationary distributions of DAF, $p$, is given by

$$f(p; S) = \frac{2\mu}{p(1-p)} \frac{1 - e^{-S(1-p)}}{1 - e^{-S}},$$

where $S = 2Ns$ and $\mu$ is the mutation rate per site per generation (Sawyer and Hartl 1992[4]; see Figure S20 for the distributions with various $S$). The variants in the genome were assumed to be independent of one another and the derived alleles were classified into three types: null

alleles ($\beta = \beta_{(0)} = 0$) with $S = S_{(0)} = 0$ (i.e., selectively neutral), risk alleles ($\beta = \beta_{(1)} > 0$) with $S = S_{(1)}$, and protective alleles ($\beta = \beta_{(2)} < 0$) with $S = S_{(2)}$. We assumed that $|\beta_{(2)}| = |\beta_{(1)}|$. When we draw a variant with a particular DAF from the genome, the probability that the variant has the effect $\beta = \beta_{(t)}$ is expressed as

$$P(\beta_{(t)}|p) = \frac{\mu_{(t)} f(p; S_{(t)})}{\sum_{t=0}^{2} \mu_{(t)} f(p; S_{(t)})},$$

where $\mu_{(t)}$ is the mutation rates for null ($t$ = 0), risk ($t$ = 1) and protective ($t$ = 2) mutations standardized by $\mu_0$, i.e., $\mu_0 = 1$. We applied the present model to random-pruned sets for each GWAS. For the $j$-th SNP the likelihood of the $y_j$ (the estimate of log-odds ratio) is given by

$$\sum_{t=0}^{2} P(\beta_{(t)}|p) \varphi_{\beta_{(t)}, \hat{V}_{\widehat{\beta_j}}}(y_j),$$

assuming the standard asymptotic normality for $y_j$. The likelihood for the random-pruned set was calculated as the product of the likelihood over all variants in the set.

For each random-pruned set, the value of $\beta_{(1)}$ was determined by the result of SP-HMM analysis (Figure 2), i.e., $\beta_{(1)}$ was given by the expectation of $|\beta|$ under $\beta \sim \hat{g}$. We used the R function 'optim' to maximize the log-likelihood for several selection pressures (Figure S21). We started with the simplest model, 'neutral mutation model' (Model 1; $S_1 = S_2 = 0$), and subsequently considered the more complex models, 'deleterious-risk mutation model' (Model 2; $S_1 < 0$ and $S_2 = 0$) and 'deleterious-risk mutation and advantageous-protective mutation model' (Model 3; $S_1 < 0$ and $S_2 > 0$). In Model 3 we assumed that $|S_1| = |S_2|$. The 'advantageous-risk mutation model' (Model 2'; $S_1 > 0$ and $S_2 = 0$) and the 'advantageous-risk mutation and deleterious-protective mutation model' (Model 3; $S_1 > 0$ and $S_2 < 0$) were also fitted but shown only in bipolar disorder since only that disease showed a better fit than the 'neutral mutation model'. Twice the negative log-likelihood value, −2log(L), setting $-2\text{Log(L)}$ of Model 1 to 0 as reference, was used for assessing the model fitting.

URLs

qvalue R package, https://www.bioconductor.org/packages/release/bioc/html/qvalue.html.

REFERENCE


1. McVean, G. A. *et al.* An integrated map of genetic variation from 1,092 human genomes. *Nature* **491,** 56–65 (2012).
2. Purcell, S. *et al.* PLINK: A tool set for whole-genome association and population-based linkage analyses. *Am. J. Hum. Genet.* **81,** 559–575 (2007).
3. Han, B., Kang, H. M. & Eskin, E. Rapid and accurate multiple testing correction and power estimation for millions of correlated markers. *PLoS Genet.* **5,** (2009).
4. Sawyer, S. A. & Hartl, D. L. Population genetics of polymorphism and divergence. *Genetics* **132,** 1161–1176 (1992).

# Supplemental Data

Empirical Bayes estimation of semi-parametric hierarchical mixture models for characterization of polygenic disease architectures

# Contents

## Supplemental Tables


- Table S1a . Settings of the simulation studies using normal distributions.
- Table S1b. Settings of the simulation studies using gamma distributions.
- Table S2. Summary of the estimated proportions of disease-associated SNPs, $\hat{\pi}$, and effect-size distributions, $\hat{g}$, in the settings using normal distributions ($m$=100000, 50000, 20000)
- Table S3. GWAS data used in this study.
- Table S4. Study type used in this study.
- Table S5. Estimated proportions of disease-associated SNPs, $\hat{\pi}$, and liability-scale variance explained by SNPs, $\hat{V}$, using the random-pruned SNP sets.
- Table S6. Estimated liability-scale variance explained by eQTL-SNPs and non-eQTL-SNPs.
- Table S7. Execution time of the SP-HMM estimation.


## Supplemental Figures

### *Simulations using normal distributions*


- Figure S1. Estimated proportions of disease-associated SNPs, $\hat{\pi}$, in the settings using normal distributions ($m$=100000, 50000, 20000).
- Figure S2. Estimated liability-scale variance, $\hat{V}$, in the settings using normal distributions ($m$=100000, 50000, 20000).
- Figure S3. Estimated effect-size distributions, $\hat{g}$, in the settings using normal distributions ($m$=100000, 50000, 20000).
- Figure S4. Estimated proportions of disease-associated SNPs, $\hat{\pi}$, in the DAF-stratified analysis.
- Figure S5. Estimated effect-size distributions, $\hat{g}$, in the DAF-stratified analysis.
- Figure S6. Raw effect-size estimates.


### *Simulations using gamma distributions*


- Figure S7. Estimated proportions of disease-associated SNPs, $\hat{\pi}$, in the settings using gamma distributions ($m$=100000).
- Figure S8. Estimated liability-scale variance, $\hat{V}$, in the settings using gamma distributions ($m$=100000).
- Figure S9. Estimated effect-size distributions, $\hat{g}$, in the settings using gamma distributions ($m$=100000).

## Supplemental Figures (continue)

***Simulations using estimated genetic architecture from real data***

- Figure S10. Estimated proportions of disease-associated SNPs, $\hat{\pi}$, under the estimated genetic architectures from the P-value-based pruned SNPs.
- Figure S11. Estimated proportions of disease-associated SNPs, $\hat{\pi}$, under the estimated genetic architectures from the eQTL/not eQTL SNPs.
- Figure S12. Estimated proportions of disease-associated SNPs, $\hat{\pi}$, under the estimated genetic architectures from the DAF stratified SNPs.
- Figure S13. Estimated effect-size distributions, $\hat{g}$, under the estimated genetic architectures from the P-value-based pruned SNPs.
- Figure S14. Estimated effect-size distributions, $\hat{g}$, under the estimated genetic architectures from the eQTL/not eQTL SNPs.
- Figure S15. Estimated effect-size distributions, $\hat{g}$, under the estimated genetic architectures from the DAF stratified SNPs.

***Simulations for the case with weak linkage disequilibrium (LD)***

- Figure S16. Estimated proportions of disease-associated SNPs, $\hat{\pi}$, in the settings with SNPs in weak LD.
- Figure S17. Estimated effect-size distributions, $\hat{g}$, in the settings with SNPs in weak LD.

***Real data analyses***

- Figure S18. Raw effect size estimates, $\hat{\beta}$, for the P-value-based pruned SNPs in the GWAS summary data .
- Figure S19. Estimated effect-size distributions for disease-associated SNPs, $\hat{g}$, using the random-pruned SNPs compared with those using the P-value-based SNPs.
- Figure S20. The stationary distributions of DAF for various values of the selection parameter.
- Figure S21. Result of fitting the three selection models.

## Supplemental Note

1. Simulation
1.1. Settings
1.2. Results
2. Exploring natural selection

Table S1a. Settings of the simulation studies using normal distributions

| Setting | Proportion of associated SNP, $\pi$ (%) | Effect-size distribution for associated SNPs, $g$ [a] | |
|---|---|---|---|
| Less polygenic | 3.0 | $\beta \sim$ Normal distribution with $E(\beta) = 0$, $V(\beta) = 0.03^2$ | |
| Less polygenic (Asymmetric) | 3.0 | Negative effects $\beta \sim$ Negative truncated normal distribution with $E(\beta) = 0$, $V(\beta) = 0.03^2$ | Positive effects $\beta \sim$ Positive truncated normal distribution with $E(\beta) = 0$, $V(\beta) = 0.04^2$ |
| Moderate polygenic | 10.0 | $\beta \sim$ Normal distribution with $E(\beta) = 0$, $V(\beta) = 0.03^2$ | |
| Moderate polygenic (Asymmetric) | 10.0 | Negative effects $\beta \sim$ Negative truncated normal distribution with $E(\beta) = 0$, $V(\beta) = 0.03^2$ | Positive effects $\beta \sim$ Positive truncated normal distribution with $E(\beta) = 0$, $V(\beta) = 0.04^2$ |
| Highly polygenic | 30.0 | $\beta \sim$ Normal distribution with $E(\beta) = 0$, $V(\beta) = 0.015^2$ | |
| Highly polygenic (Asymmetric) | 30.0 | Negative effects $\beta \sim$ Negative truncated normal distribution with $E(\beta) = 0$, $V(\beta) = 0.015^2$ | Positive effects $\beta \sim$ Positive truncated normal distribution with $E(\beta) = 0$, $V(\beta) = 0.02^2$ |

[a] In the asymmetric cases, the mixing ratios of positive and negative distributions was determined so that the whole (mixed) effect-size distribution, $g$, are continuous functions.

Table S1b. Settings of the simulation studies using beta distributions

| Setting | Proportion of associated SNP, $\pi$ (%) | Effect-size distribution for associated SNPs, $g$ | | |
|---|---|---|---|---|
| | | Negative effects ($\beta < 0$) [a] | Positive effects ($\beta > 0$) [a] | |
| Less polygenic | 3.0 | $(-\beta) \sim$ Gamma distribution with $E(\beta) = 0.03$, $V(\beta) = 0.015^2$ | $\beta \sim$ Gamma distribution with $E(\beta) = 0.03$, $V(\beta) = 0.015^2$ | |
| Less polygenic with large effects | 3.0 | $(-\beta) \sim$ Gamma distribution with $E(\beta) = 0.03$, $V(\beta) = 0.015^2$ | 95 % of $\beta \sim$ Gamma distribution with $E(\beta) = 0.03$, $V(\beta) = 0.015^2$ | 5 % of $\beta = 0.1$ |
| Moderate polygenic | 10.0 | $(-\beta) \sim$ Gamma distribution with $E(\beta) = 0.03$, $V(\beta) = 0.015^2$ | $\beta \sim$ Gamma distribution with $E(\beta) = 0.03$, $V(\beta) = 0.015^2$ | |
| Moderate polygenic with large effects | 10.0 | $(-\beta) \sim$ Gamma distribution with $E(\beta) = 0.03$, $V(\beta) = 0.015^2$ | 95 % of $\beta \sim$ Gamma distribution with $E(\beta) = 0.03$, $V(\beta) = 0.015^2$ | 5 % of $\beta = 0.1$ |
| Highly polygenic | 30.0 | $(-\beta) \sim$ Gamma distribution with $E(\beta) = 0.018$, $V(\beta) = 0.005^2$ | $\beta \sim$ Gamma distribution with $E(\beta) = 0.018$, $V(\beta) = 0.005^2$ | |
| Highly polygenic with large effects | 30.0 | $(-\beta) \sim$ Gamma distribution with $E(\beta) = 0.018$, $V(\beta) = 0.005^2$ | 98% of $\beta \sim$ Gamma distribution with $E(\beta) = 0.018$, $V(\beta) = 0.005^2$ | 2 % of $\beta = 0.08$ |

[a] The ratios of positive to negative effects was 2:1 in all settings.

Table S2. Summary of simulation results for the estimated proportions of disease-associated SNPs, $\hat{\pi}$, and effect-size distributions, $\hat{g}$, in the settings using normal distributions

| | Sample size ($n$) | # of SNPs ($m$): 20000 | 50000 | 100000 |
|---|---|---|---|---|
| Less polygenic | 3000 ∼ 100000 | $\pi$ slightly overestimated; Estimated $g$ with higher frequency for small effects | NUB | |
| Moderate polygenic | 3000 ∼ 100000 | NUB | | |
| Highly polygenic | 3000 | $\pi$ underestimated; Estimated $g$ with higher frequency for large effects | | |
| | 5000 | $\pi$ slightly underestimated | | |
| | 7000 ∼ 100000 | NUB | | |

The table summarizes Figures S1 and S3 using the settings for normal distributions ($m$=100000, 50000, 20000). DAF's were generated from a uniform distribution U(0.01, 0.99).
NUB: Nearly Unbiased.

Table S3. GWAS data used in this study

| | Cohorts | Cases | Controls | # of SNP ( × 1,000) | | | |
|---|---|---|---|---|---|---|---|
| | | | | All | Pruned: P- values[b] | Pruned: random | Pruned: eQTL + not eQTL (# of eQTL + not eQTL) |
| Rheumatoid arthritis[a] (Asian) | 3 (East Asian) | 4,873 | 17,642 | 4,786 | 72 | 71 | 71 (22+ 49) |
| Rheumatoid arthritis[a] (European) | 18 (European) | 14,361 | 43,923 | 5,816 | 84 | 82 | 83 (21 + 62) |
| Coronary artery disease (CARDIoGRAM) | 14 (European) | 22,233 | 64,762 | 2,325 | 81 | 80 | 80 (21 + 58) |
| Coronary artery disease (C4D) | 4 (2 European + 2 Asian) | 15,420 | 15,062 | 520 | 81 | 80 | 80 (21 + 58) |
| Schizophrenia | 52 (49 European + 3 East Asian) | 35,476 | 46,389 | 7,688 | 100 | 100 | 100 (25 + 75) |
| Bipolar disorder | 11 (European) | 7,481 | 9,250 | 2,330 | 102 | 100 | 100 (25 + 75) |

[a] For rheumatoid arthritis, MHC region (chromosome 6, 25 – 35 Mb) was removed.
[b] In each "Pruned: P- values" set, to preferentially select SNPs with strong associations, P-values of SNPs in the other study of the same disease were used. For schizophrenia (bipolar disorder), P-values in the study for bipolar disorder (schizophrenia ) were used.

Table S4. Study type used in this study

| | Study type/ Correction for population stratification |
|---|---|
| Rheumatoid arthritis (Asian) | Meta-analysis/ corrected for genomic control in each cohort |
| Rheumatoid arthritis (European) | Meta-analysis/ corrected for genomic control in each cohort, including top principal components as covariates in each cohort |
| Coronary artery disease (CARDIoGRAM) | Meta-analysis/ corrected for genomic control in each cohort |
| Coronary artery disease (C4D) | Meta-analysis/ corrected for genomic control in each cohort |
| Schizophrenia | Meta-analysis/ including top principal components as covariates in each cohort |
| Bipolar disorder | Pooled analysis/ including as covariates multidimensional scaling components |

Table S5. Estimated proportions of disease-associated SNPs, $\hat{\pi}$, and liability-scale variance explained by SNPs, $\hat{V}$ using the random-pruned SNP sets

| | $\hat{\pi}$ (SE [a]) (%) | $\hat{V}$ (SE [a]) (%) |
|---|---|---|
| Rheumatoid arthritis (Asian) | 4.4 (2.5) | 10.5 (1.4) |
| Rheumatoid arthritis (European) | 6.9 (2.2) | 17.5 (1.2) |
| Coronary artery disease (CARDIoGRAM) | 23.5 (3.6) | 18.0 (1.0) |
| Coronary artery disease (C4D) | 26.2 (3.5) | 18.2 (1.3) |
| Schizophrenia | 42.2 (1.4) | 37.0 (0.6) |
| Bipolar disorder | 35.6 (4.0) | 35.6 (1.8) |

Estimates for the random-pruned SNP sets are shown. For $\hat{V}$ , disease prevalences were assumed to be 1% for rheumatoid arthritis and schizophrenia, 6% for coronary artery disease and 0.5% for bipolar disorder.
[a]Estimated based on 100 parametric bootstrap samples based on the estimated SP-HMM.

Table S6. Estimated liability-scale variance explained by eQTL-SNPs and non-eQTL-SNPs

| | non-eQTL | | eQTL | | | |
|---|---|---|---|---|---|---|
| | $\hat{V}$ (%) | Per-SNP variance (%) × 10K[a] | $\hat{V}$ (%) | Per-SNP variance (%) × 10K[a] | Total $\hat{V}$ (%)[b] | Enrichment in eQTL[c] |
| Rheumatoid arthritis (Asian) | 1.8 (0.9) | 0.4 (0.2) | 9.5 (1.1) | 4.3 (0.5) | 11.3 (1.4) | 10.7 |
| Rheumatoid arthritis (European) | 6.5 (1.0) | 1.1 (0.2) | 12.3 (0.9) | 5.7 (0.4) | 18.8 (1.3) | 5.2 |
| Coronary artery disease (CARDIoGRAM) | 8.2 (0.9) | 1.4 (0.2) | 8.5 (0.7) | 4.0 (0.3) | 16.7 (1.1) | 2.9 |
| Coronary artery disease (C4D) | 11.2 (1.0) | 1.9 (0.2) | 7.6 (0.7) | 3.6 (0.3) | 18.8 (1.2) | 1.9 |
| Schizophrenia | 21.8 (0.5) | 2.9 (0.1) | 14.7 (0.5) | 5.9 (0.2) | 36.5 (0.7) | 2.0 |
| Bipolar disorder | 19.6 (1.3) | 2.6 (0.2) | 14.8 (1.0) | 5.9 (0.4) | 34.4 (1.6) | 2.3 |

[a]Calculated as the estimated variance divided by the numbers of non-eQTL/eQTL-SNPs.
[b]Calculated as the estimated variance by eQTL-SNPs plus the estimated variance by non-eQTL-SNPs.
[c]Fold enrichment for the per-SNP variance in eQTL-SNPs relative to that in non-eQTL-SNPs.

Table S7. Execution time of the SP-HMM estimation

| | # of SNPs ( × 1,000) | Execution time[a] |
|---|---|---|
| Pruned:P-values | 100 | 4.7 minutes |
| Pruned:random | 100 | 4.7 minutes |
| eQTL | 25 | 1.5 minutes |
| not eQTL | 75 | 3.4 minutes |

[a] Execution times for the SP-HMM estimation without bootstrapping for the schizophrenia GWAS data on a ThinkPad X1 Carbon laptop with 2.6GHz CPU and 16GB RAM.

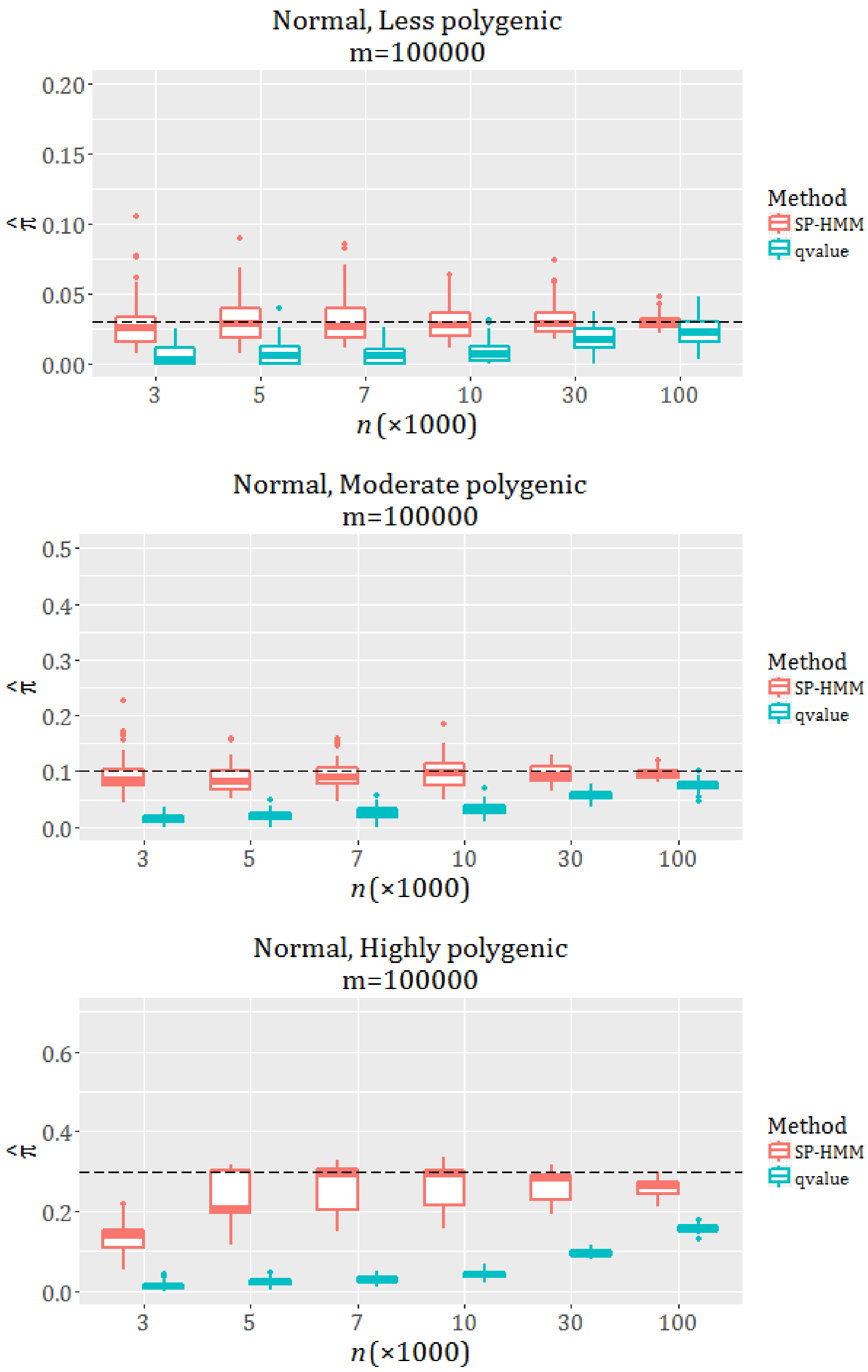


Figure S1. Estimated proportions of disease-associated SNPs, $\hat{\pi}$, in the settings using normal distributions ($m$=100000, 50000, 20000). DAF's, were generated from a uniform distribution U(0.01, 0.99). Each box plot shows 100 simulation replicates. The black dashed lines show the specified (true) $\pi$.

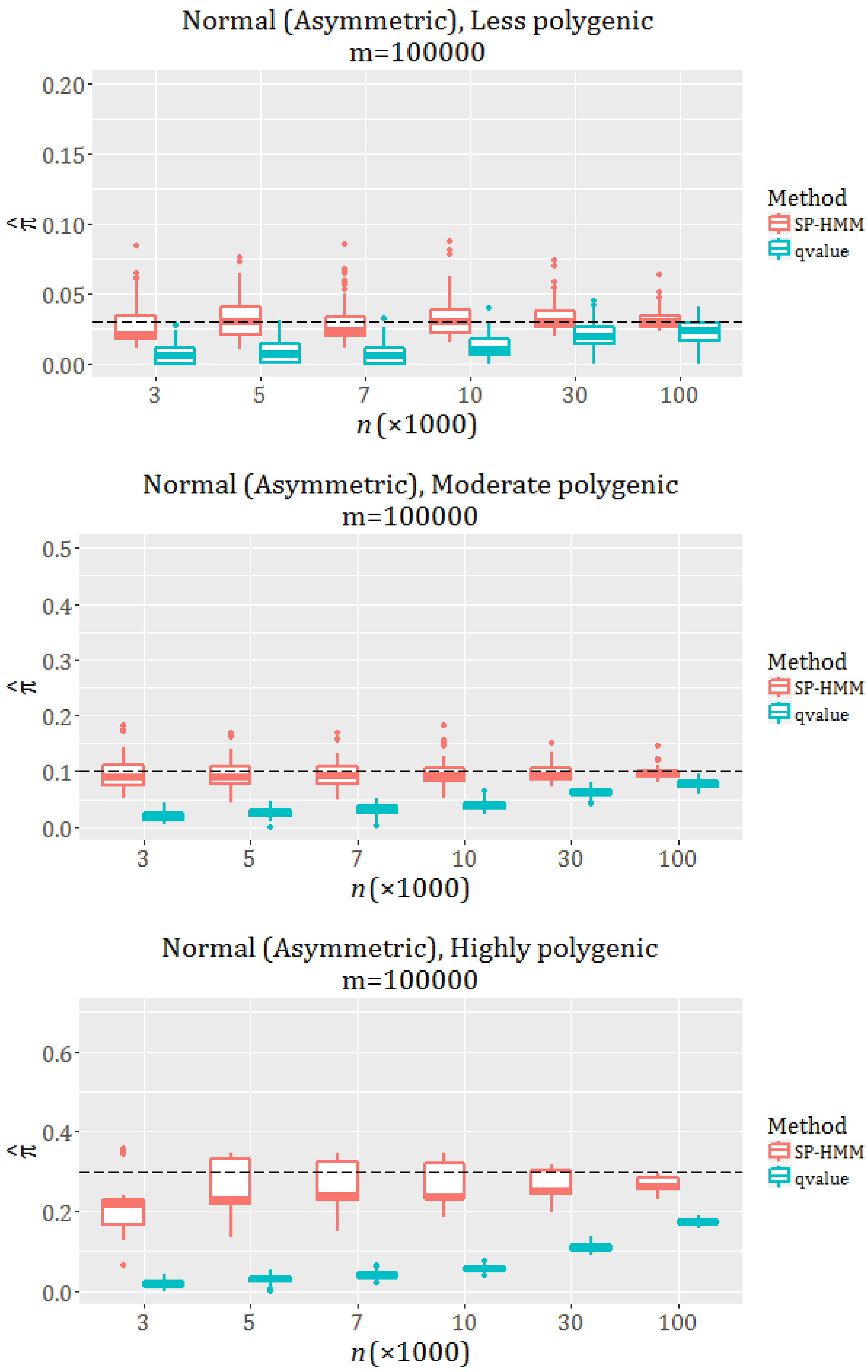


Figure S1. (Continued).

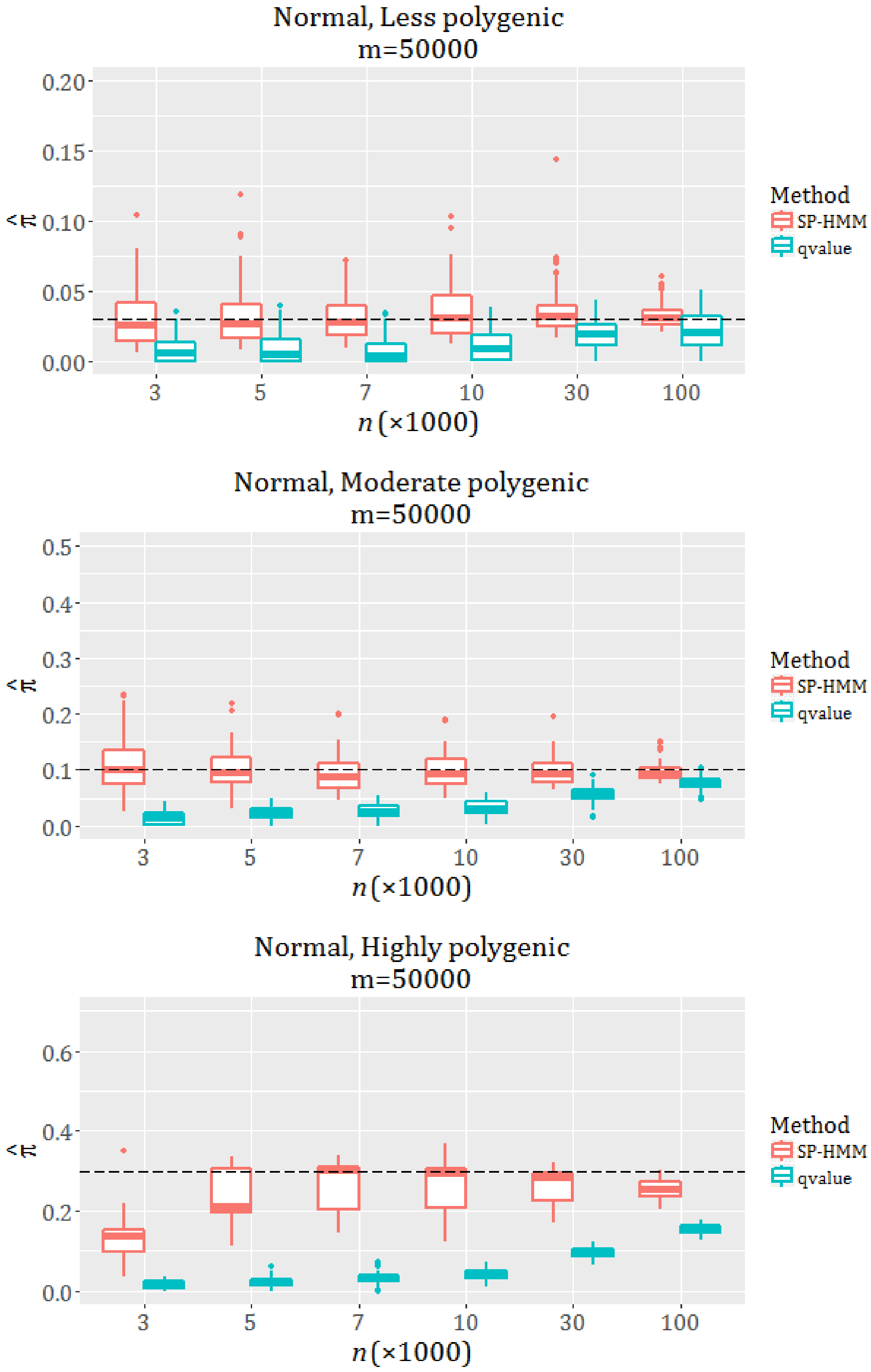


Figure S1. (Continued).

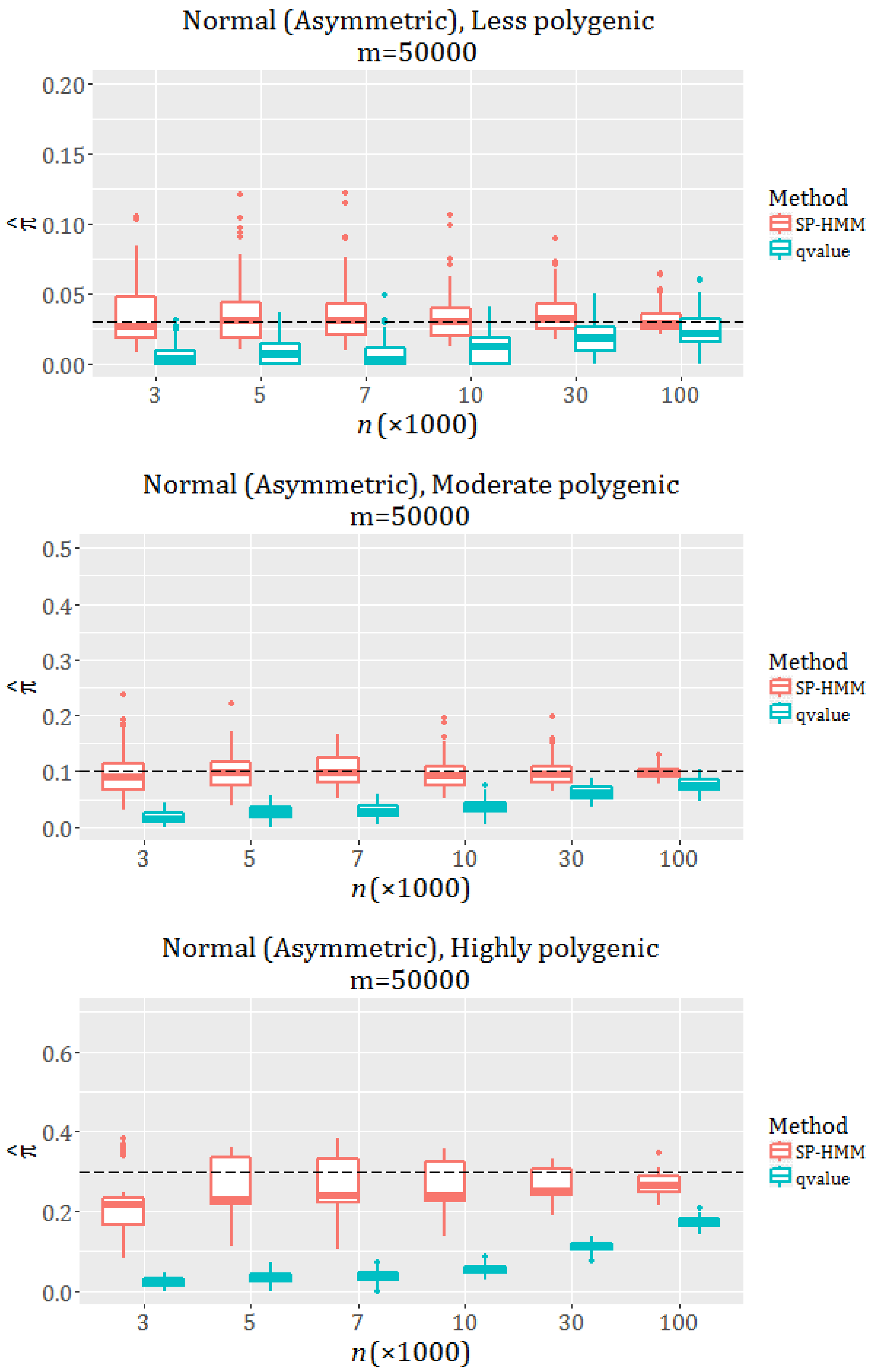


Figure S1. (Continued).

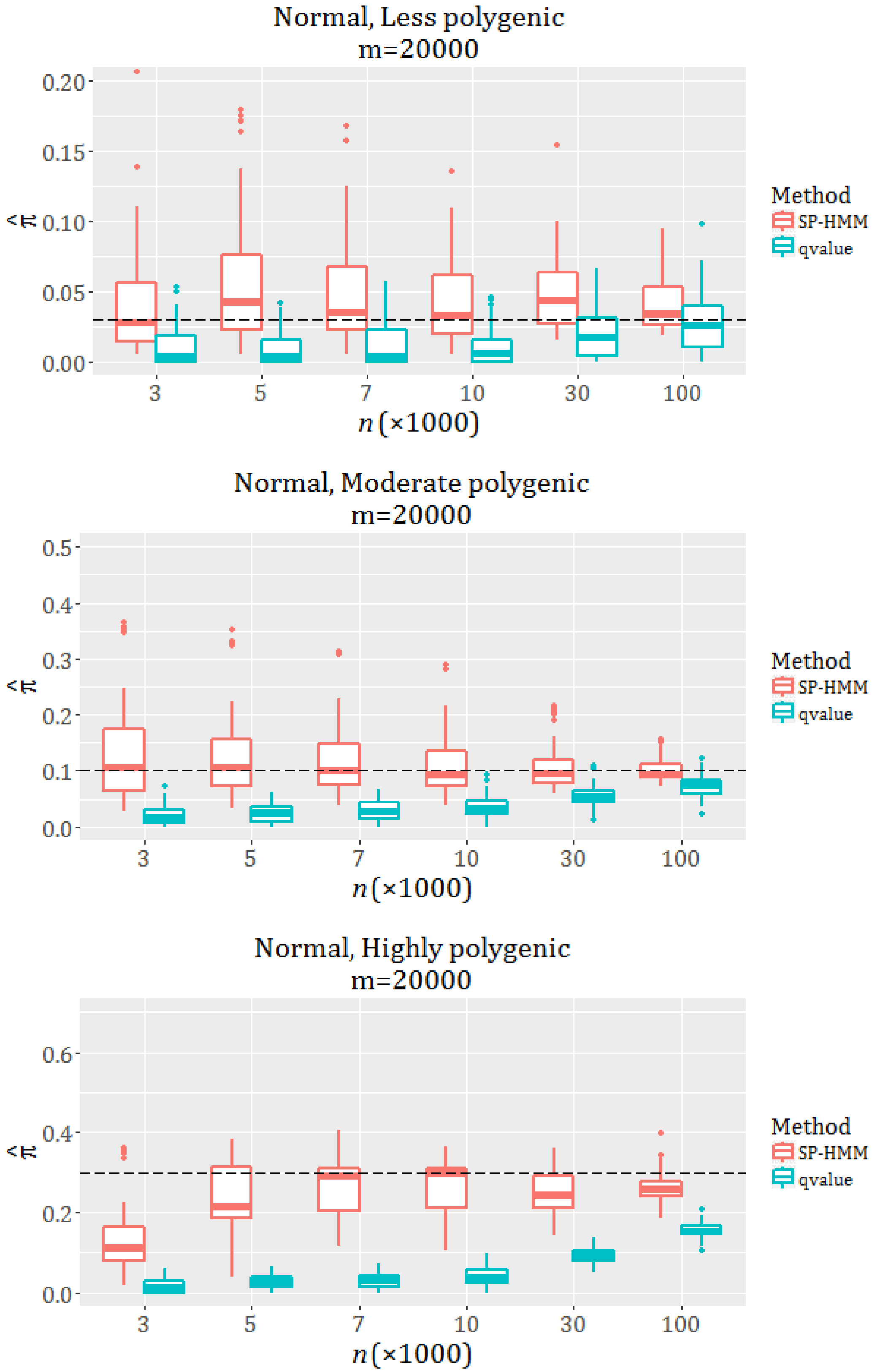


Figure S1. (Continued).

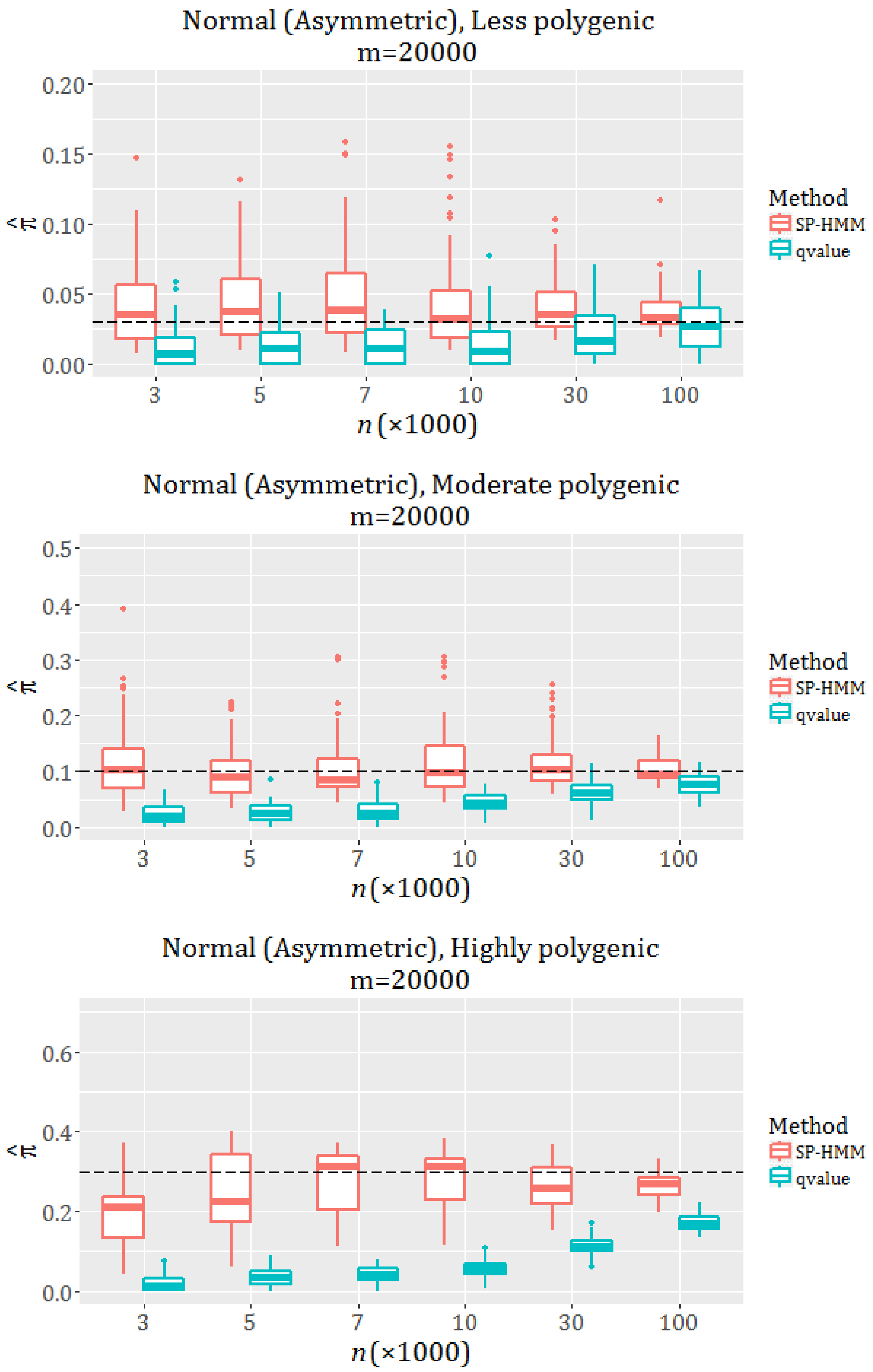


Figure S1. (Continued).

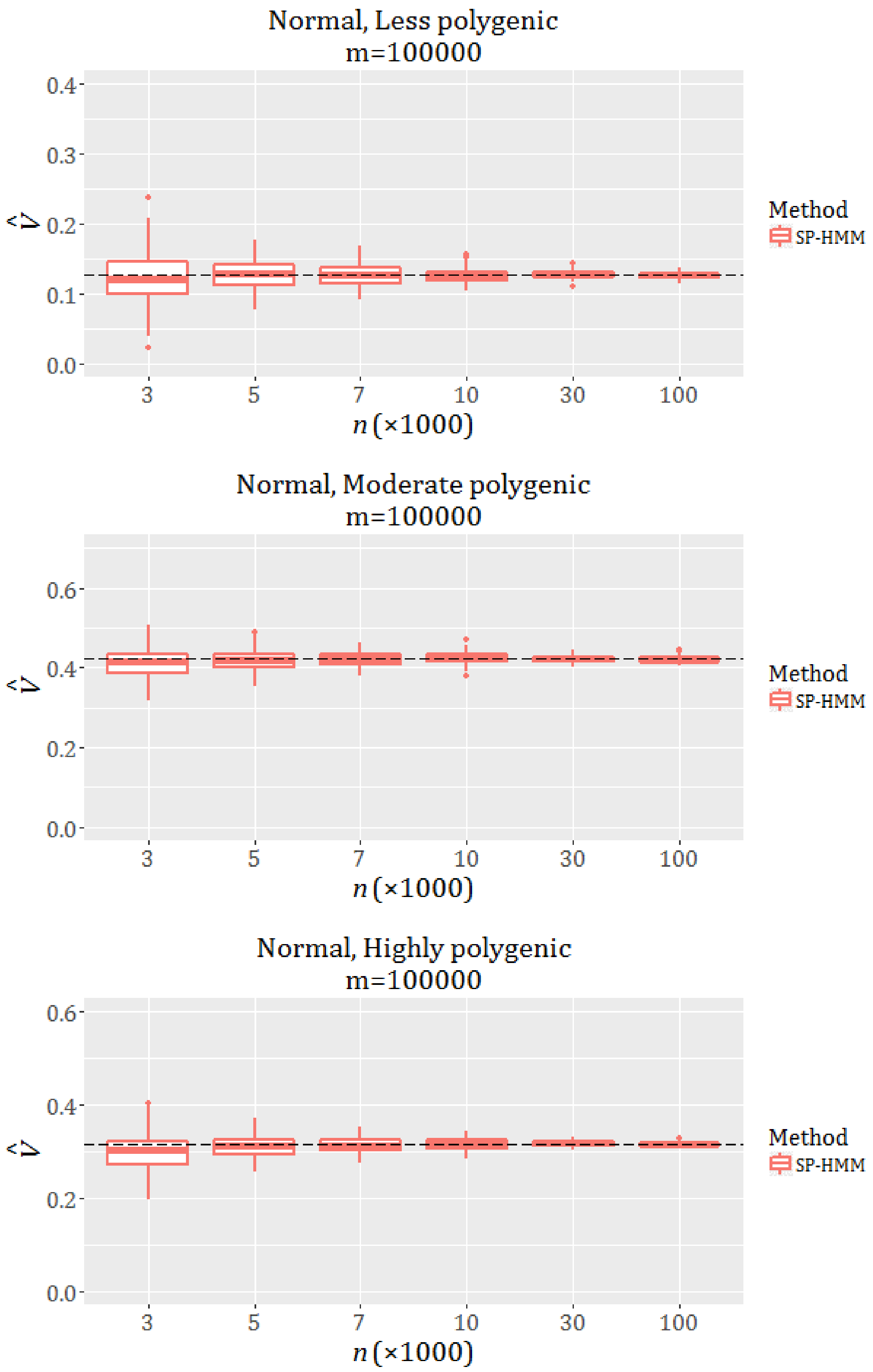


Figure S2. Estimated liability-scale variance, $\hat{V}$, in the settings using normal distributions ($m$=100000, 50000, 20000). Each box plot shows 100 simulation replicates. The black dashed lines show the specified (true) $V$.

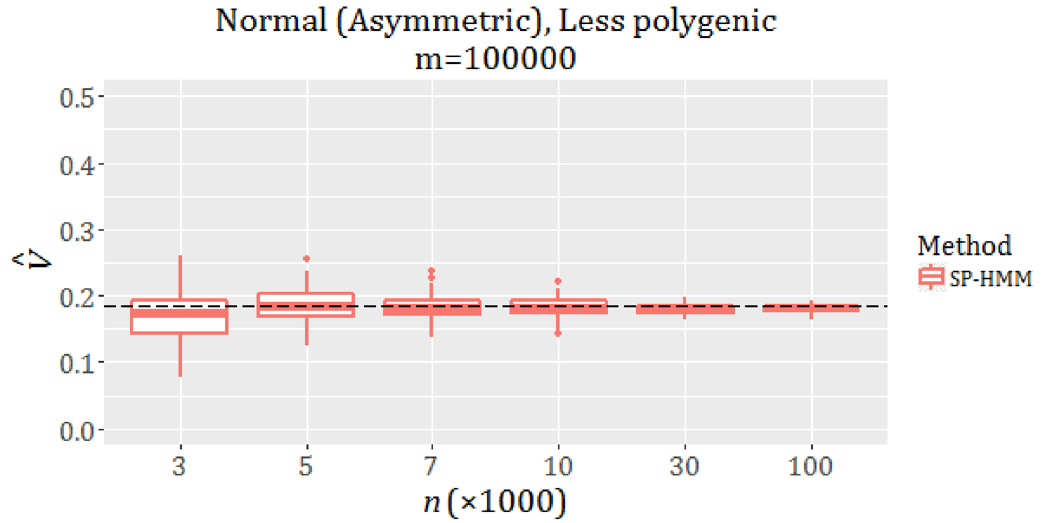


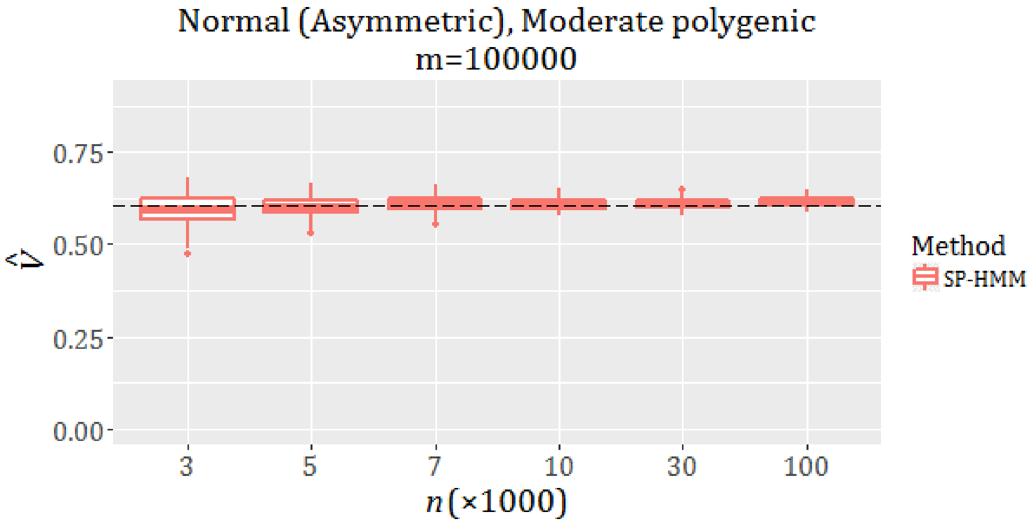


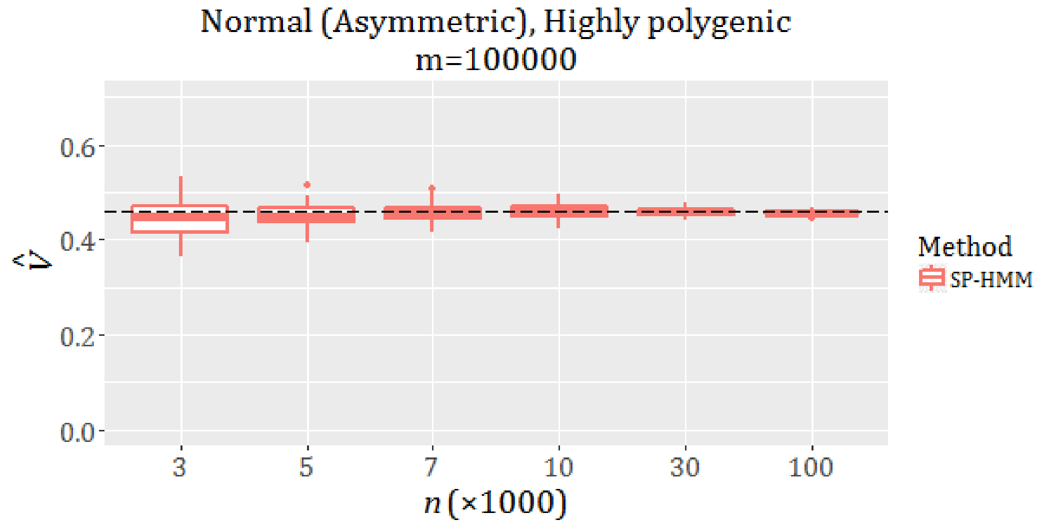


Figure S2. (Continued).

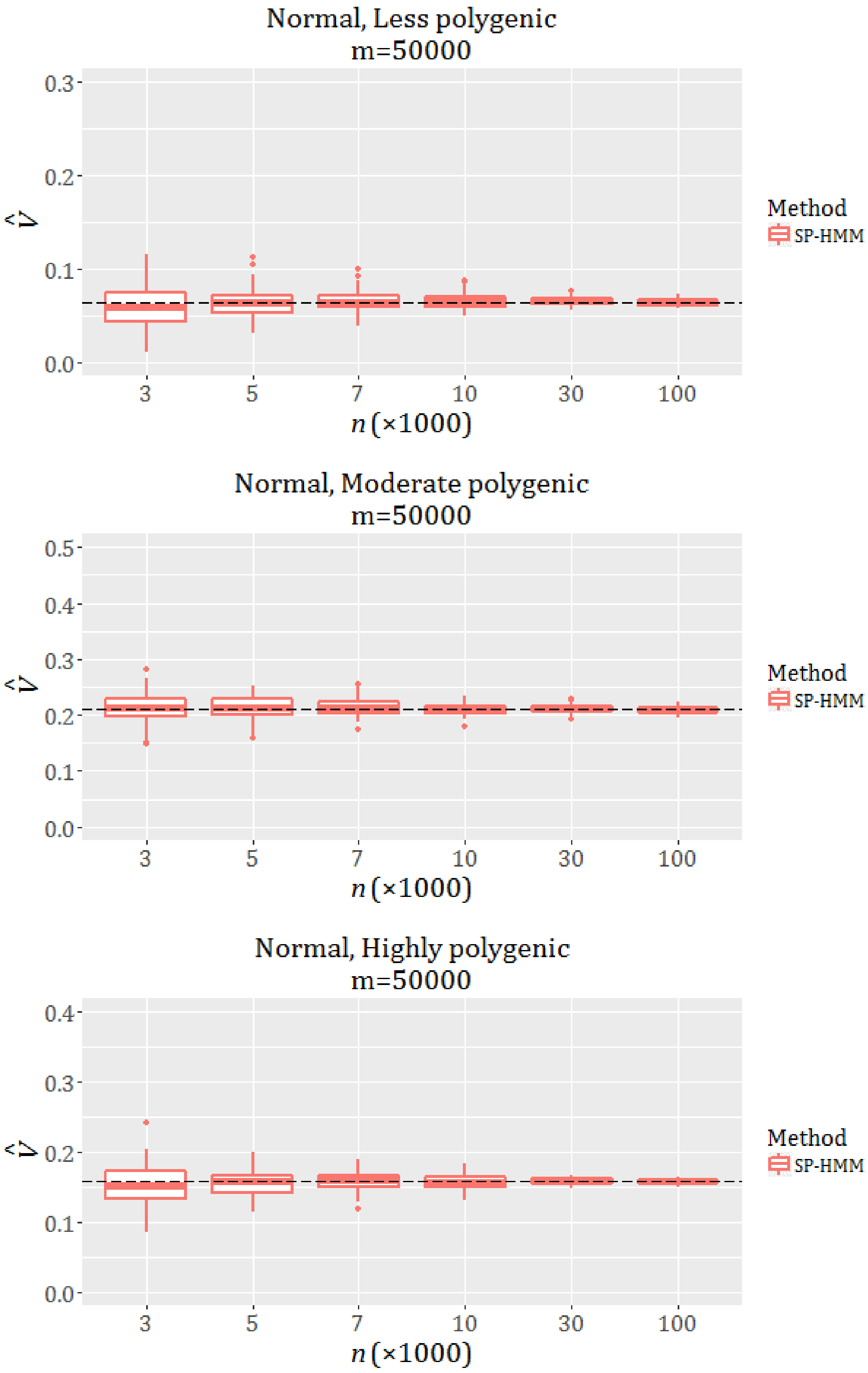


Figure S2. (Continued).

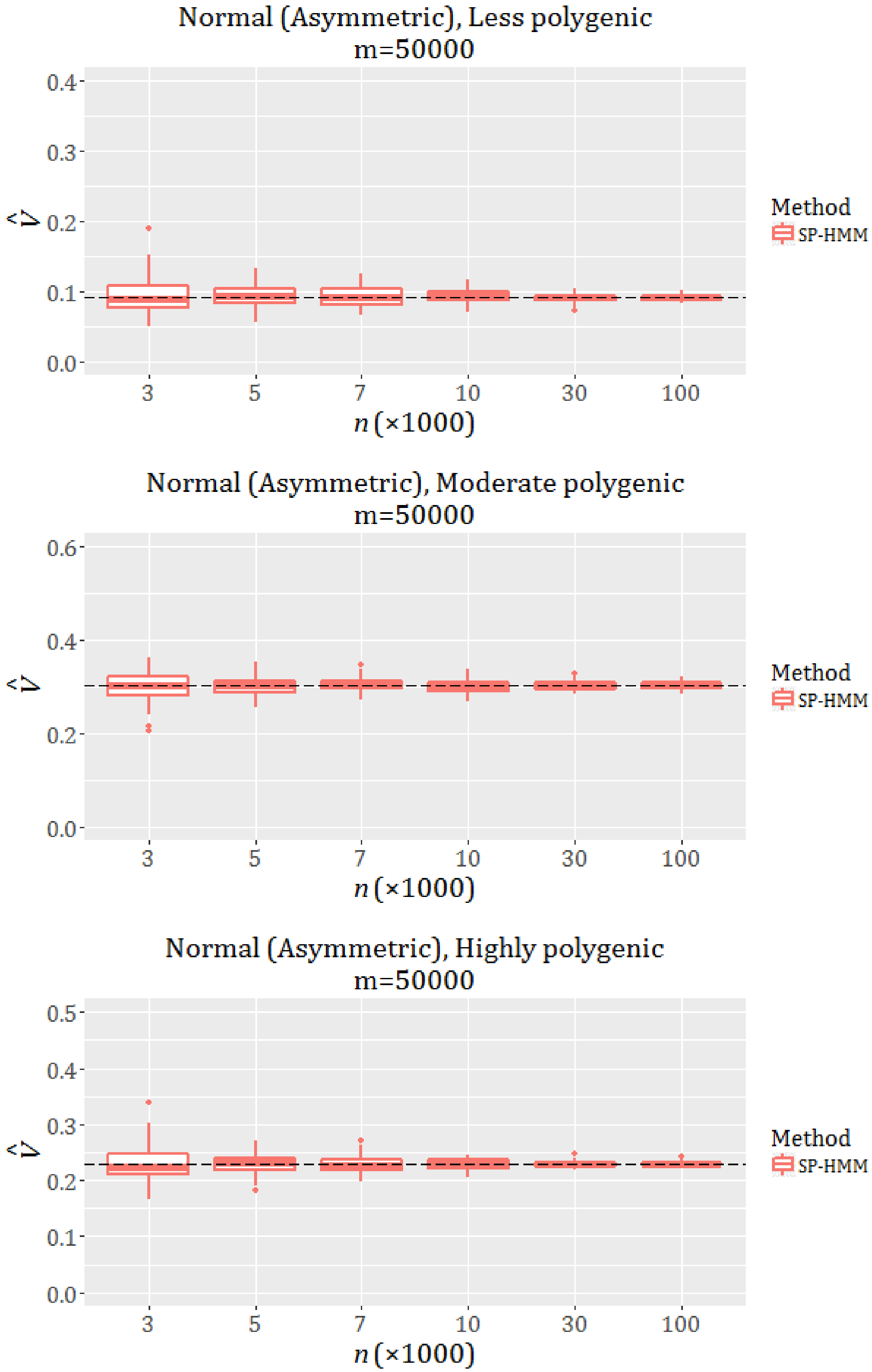


Figure S2. (Continued).

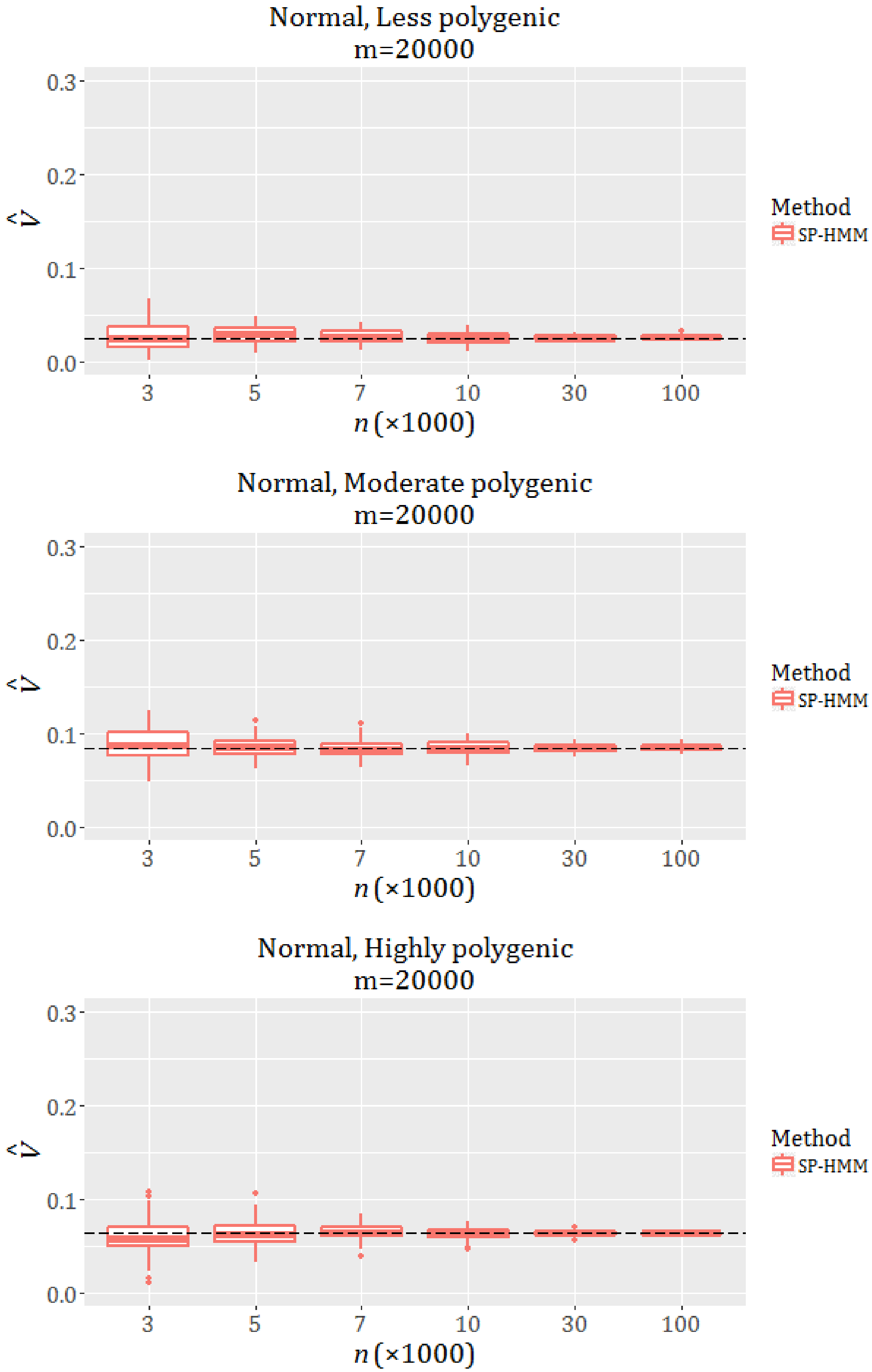


Figure S2. (Continued).

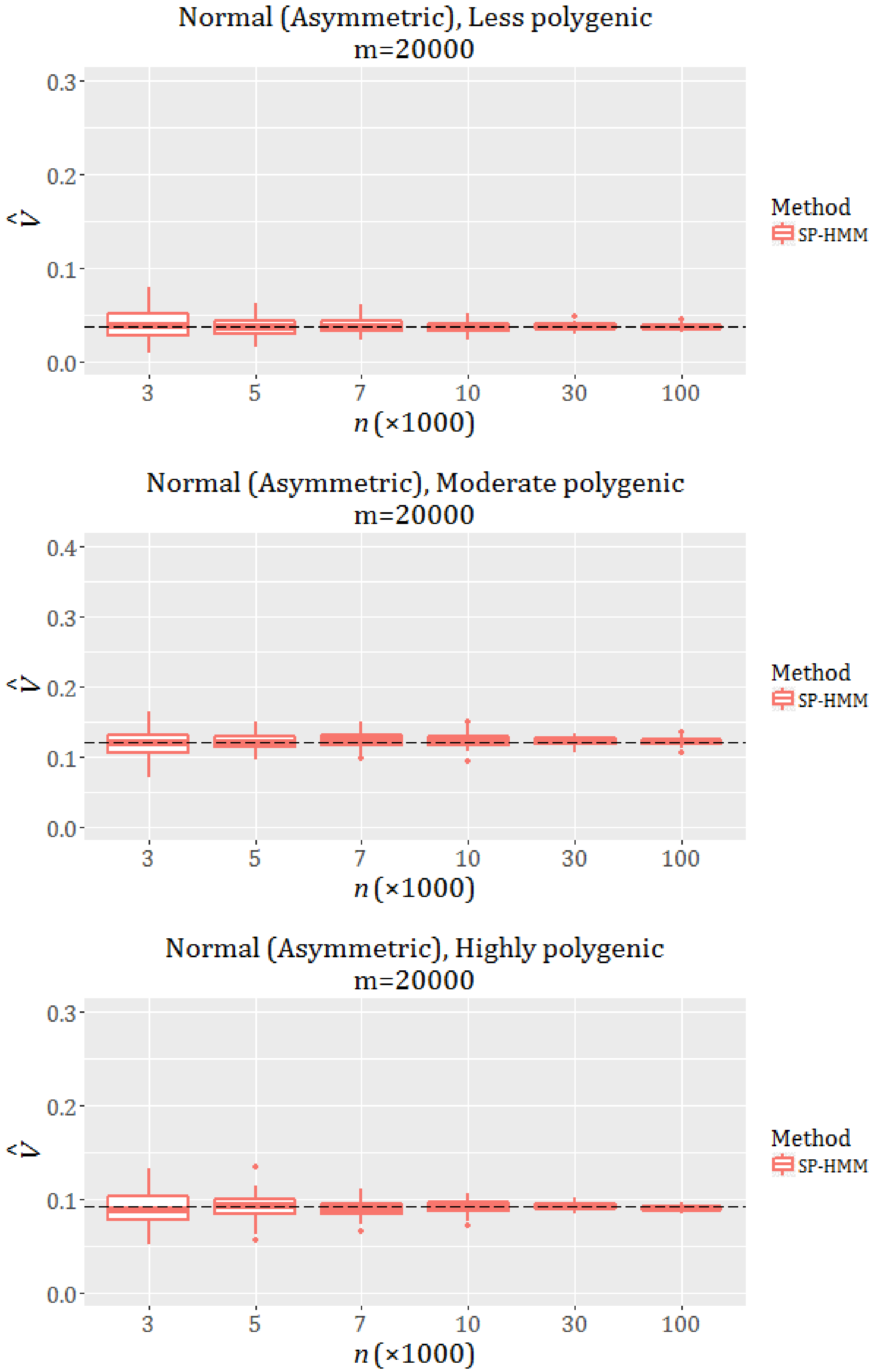


Figure S2. (Continued).

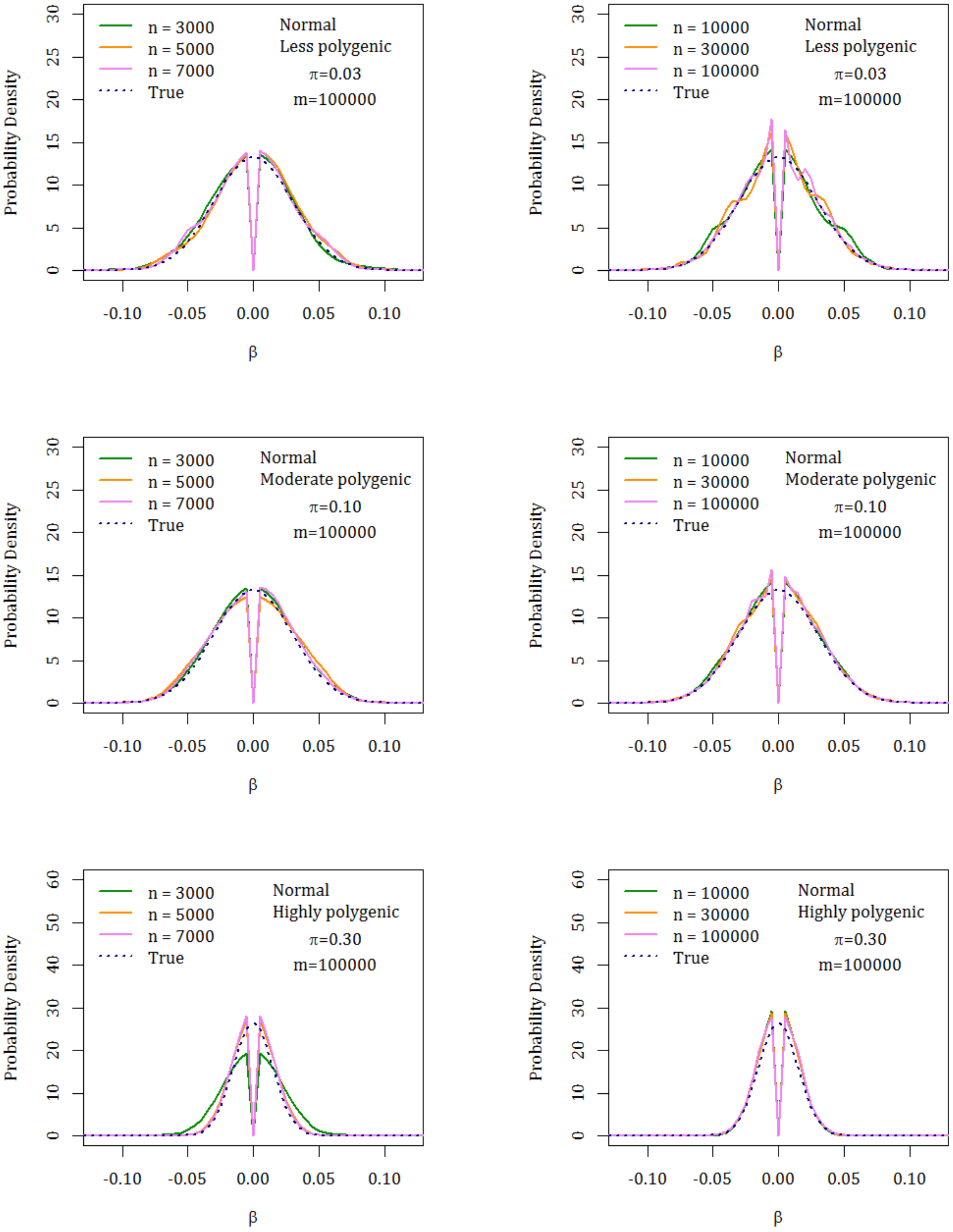


Figure S3. Estimated effect-size distributions, $\hat{g}$, in the settings using normal distributions ($m$=100000, 50000, 20000). Average curves over 100 simulations for each sample size are shown. The specified (true) effect-size distributions are given in dotted lines.

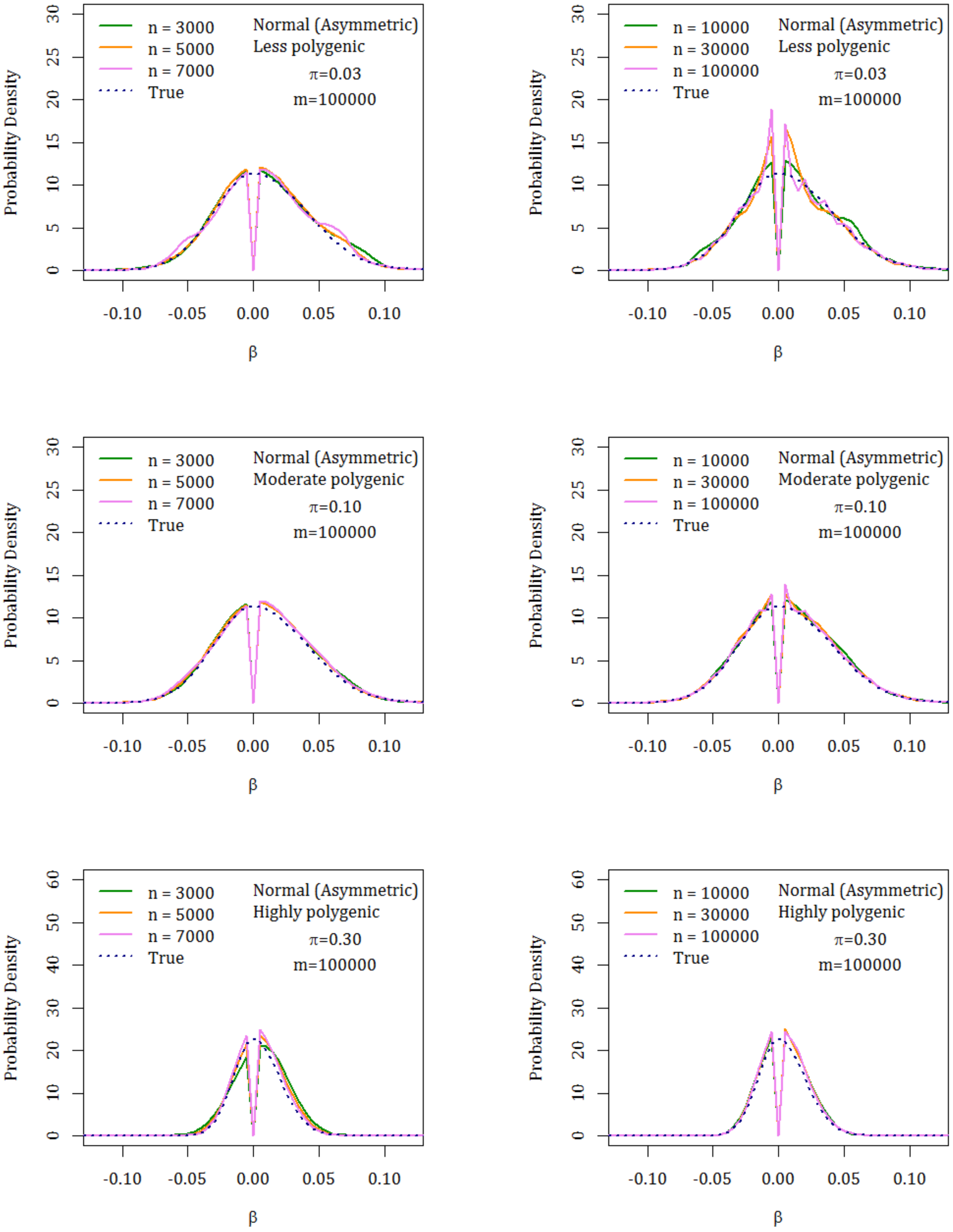


Figure S3. (Continued).

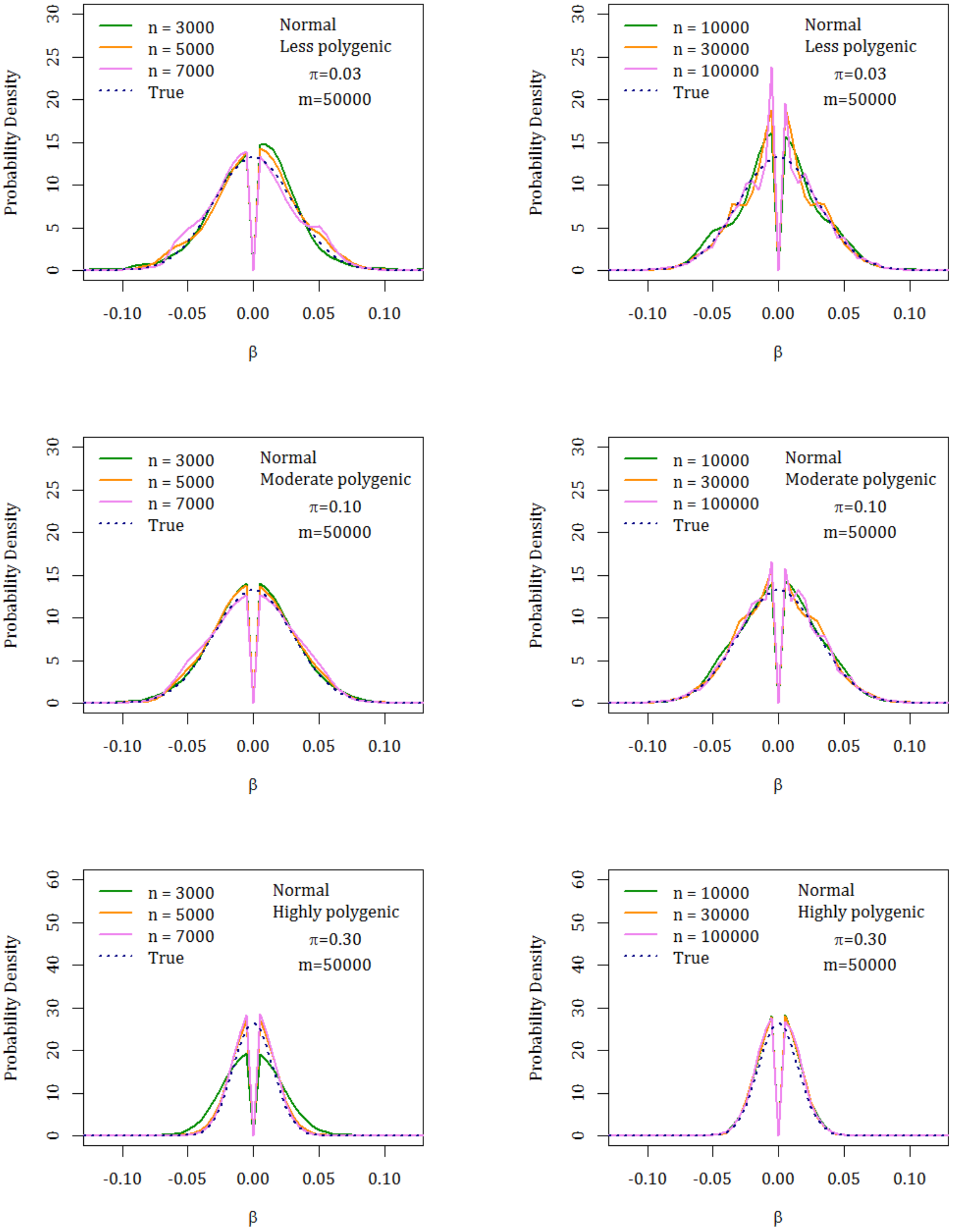


Figure S3. (Continued).

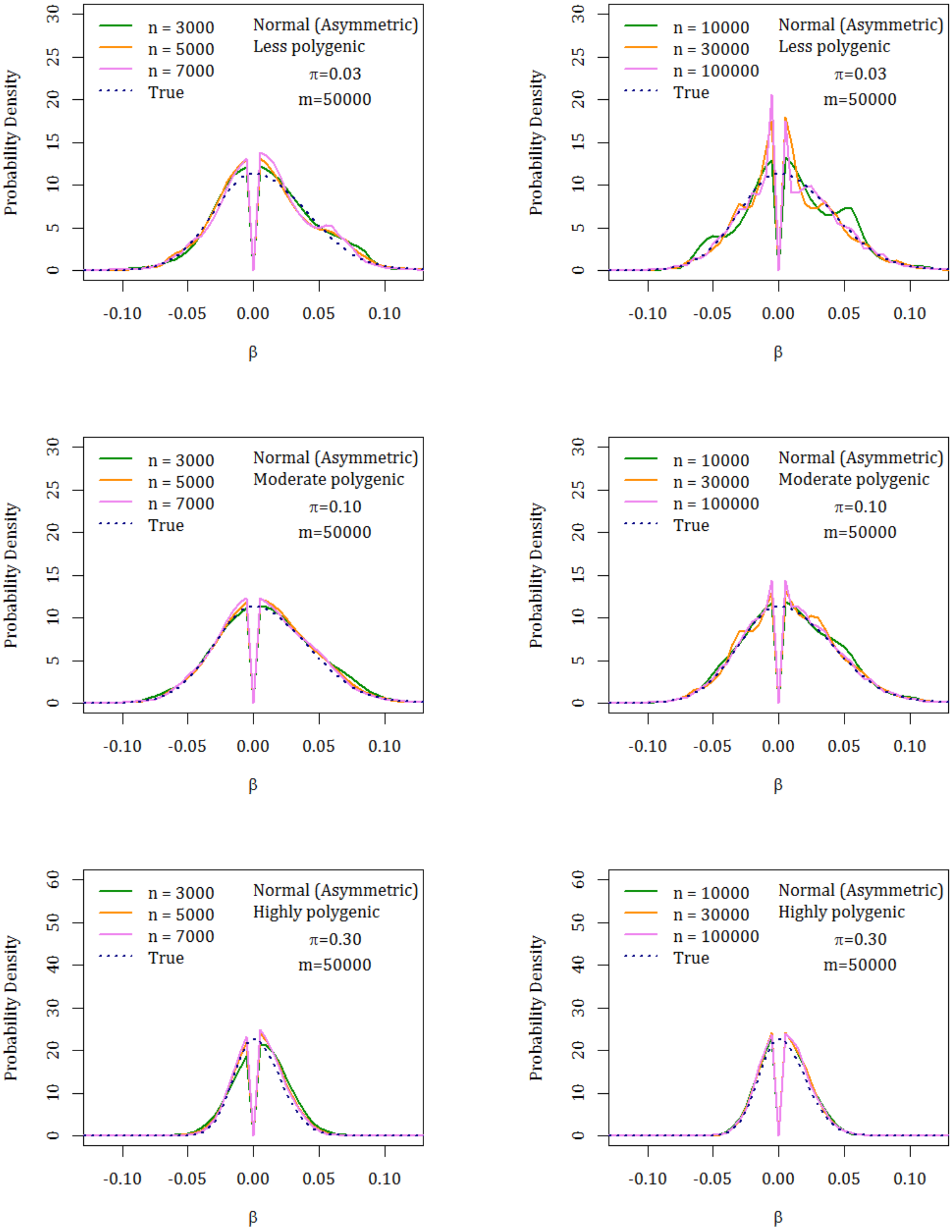


Figure S3. (Continued).

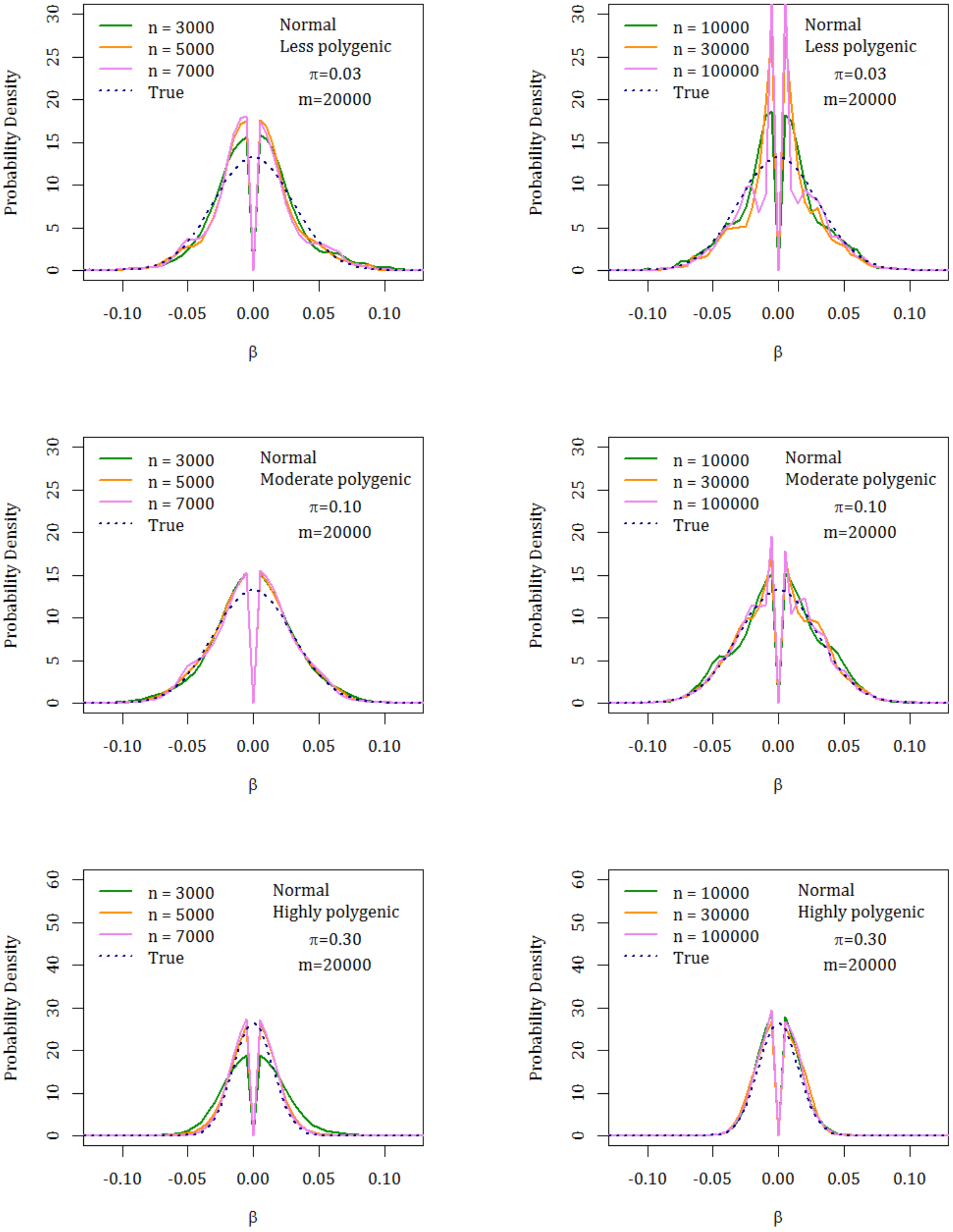


Figure S3. (Continued).

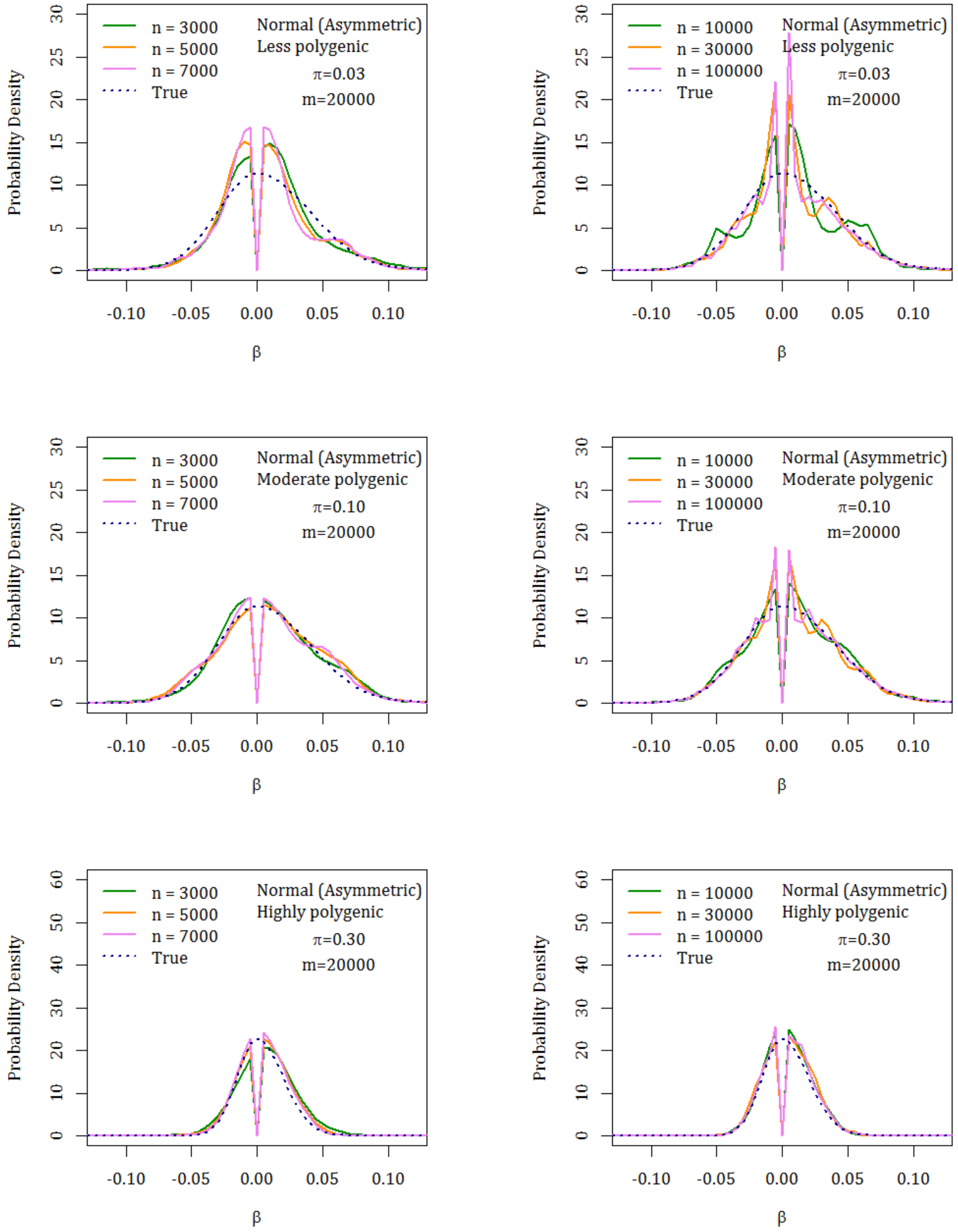


Figure S3. (Continued).

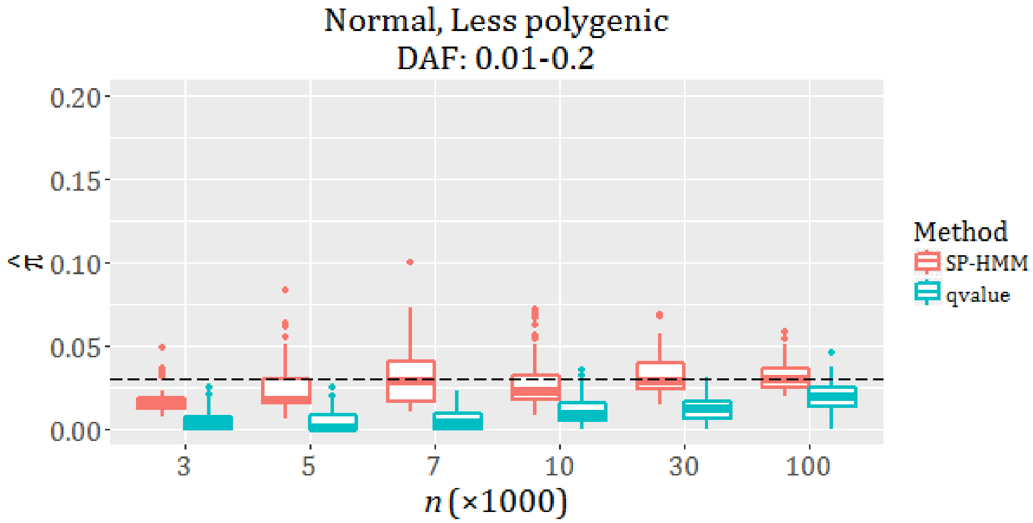


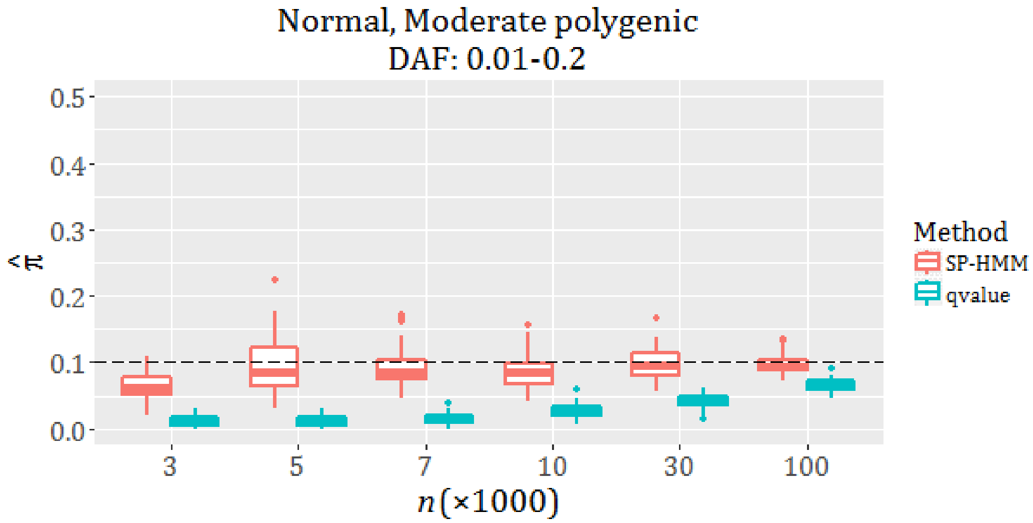


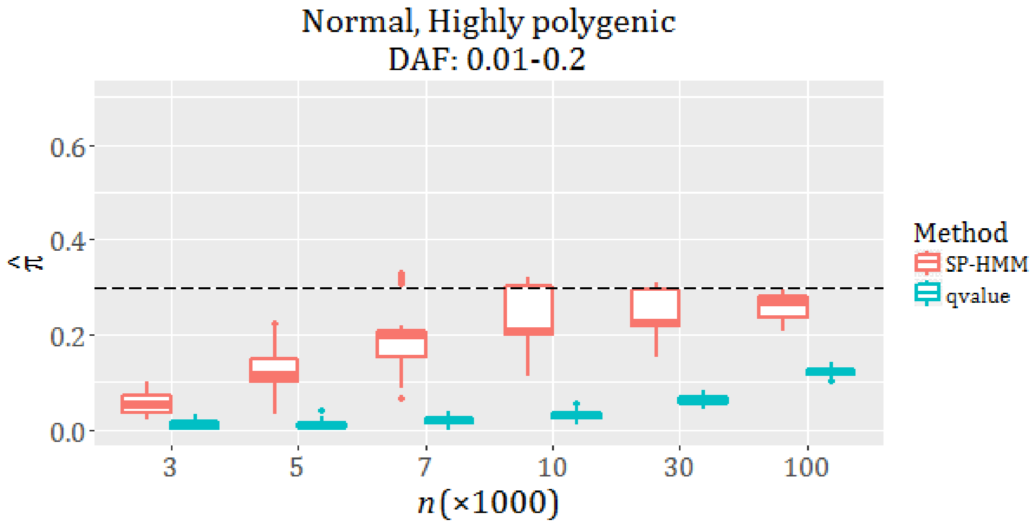


Figure S4. Estimated proportions of disease-associated SNPs, $\hat{\pi}$, in the DAF-stratified analysis. Each box plot shows 100 simulation replicates. The black dashed lines show the true $\pi$.

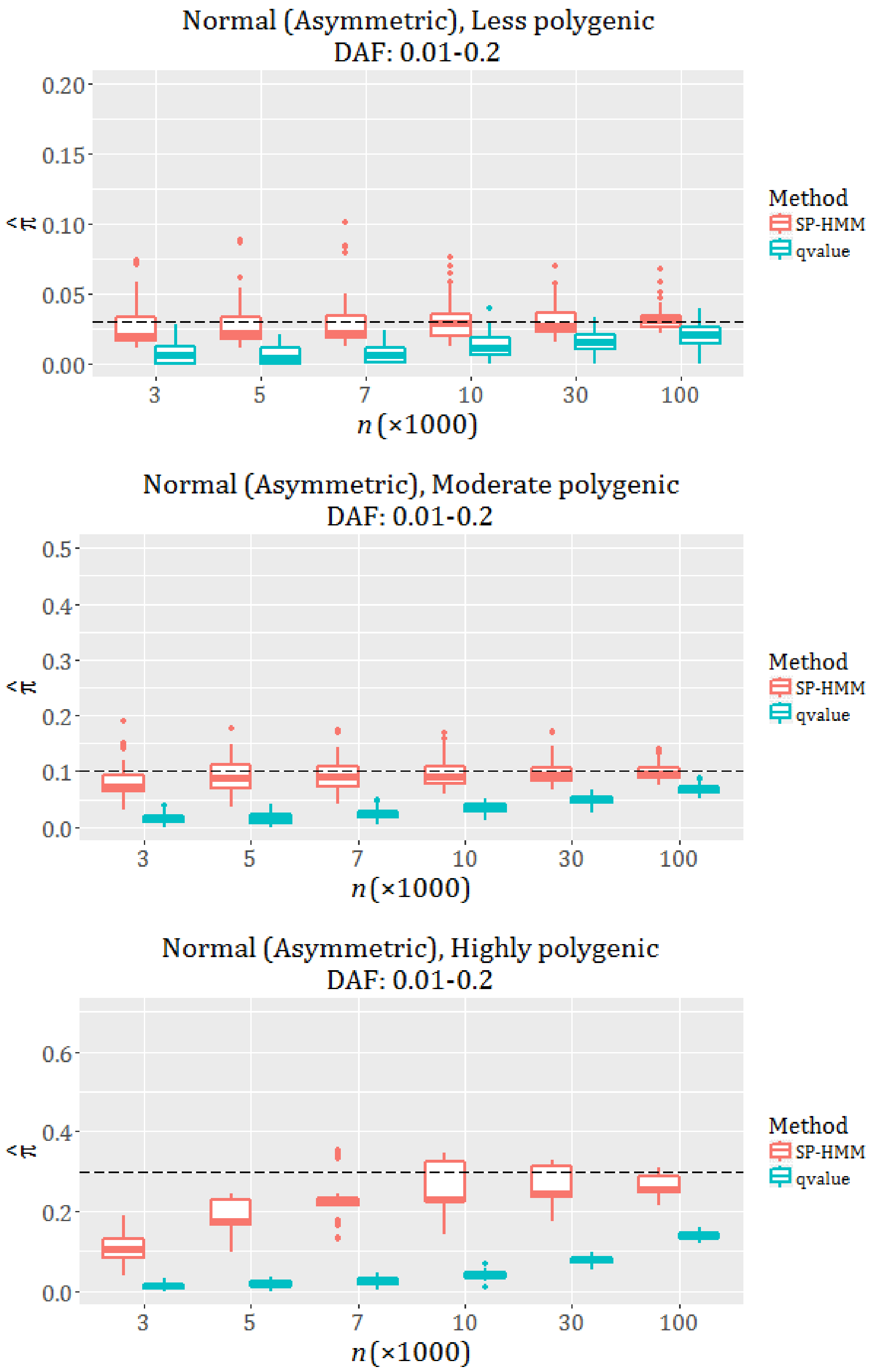


Figure S4. (Continued).

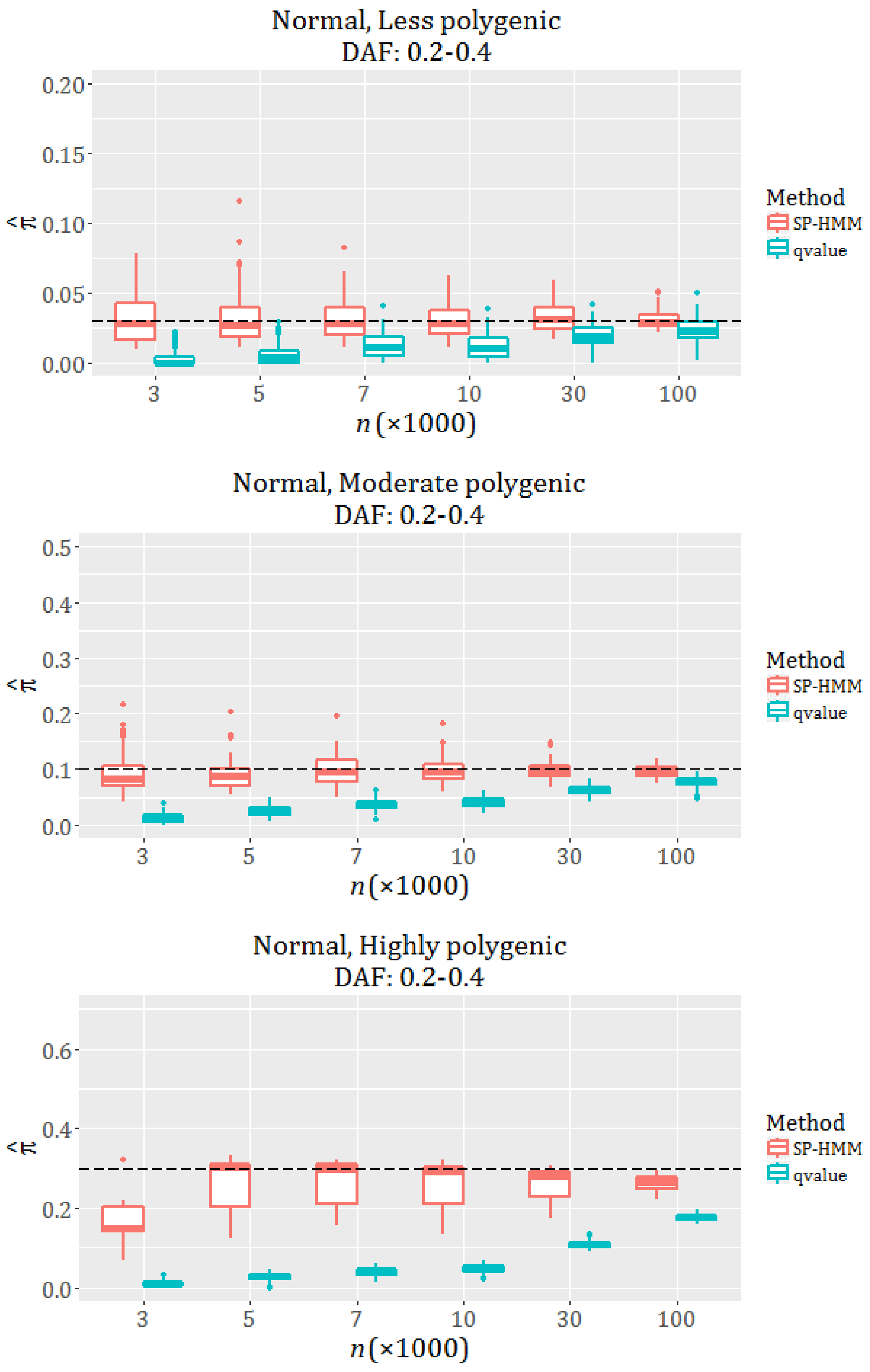


Figure S4. (Continued).

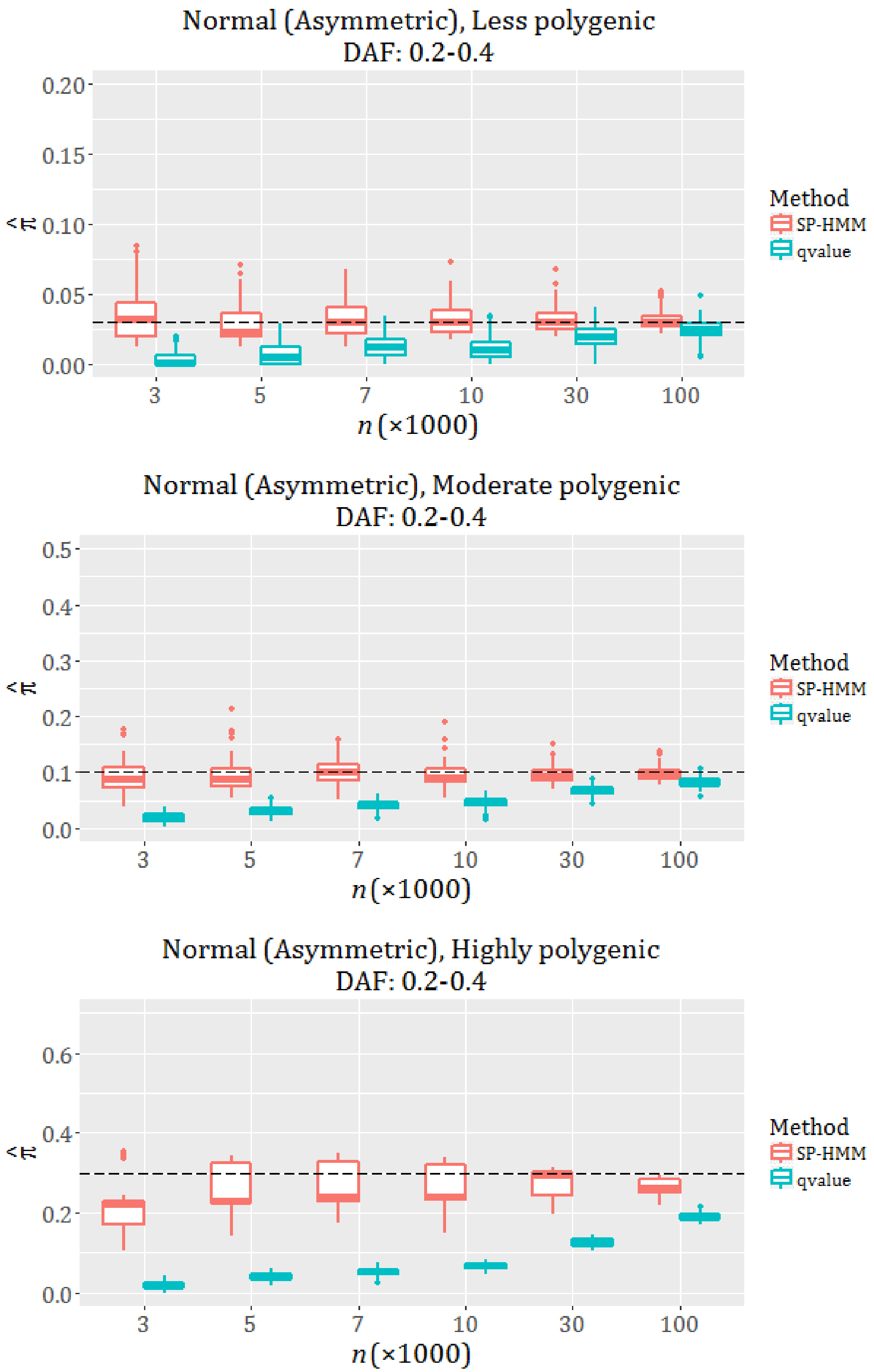


Figure S4. (Continued).

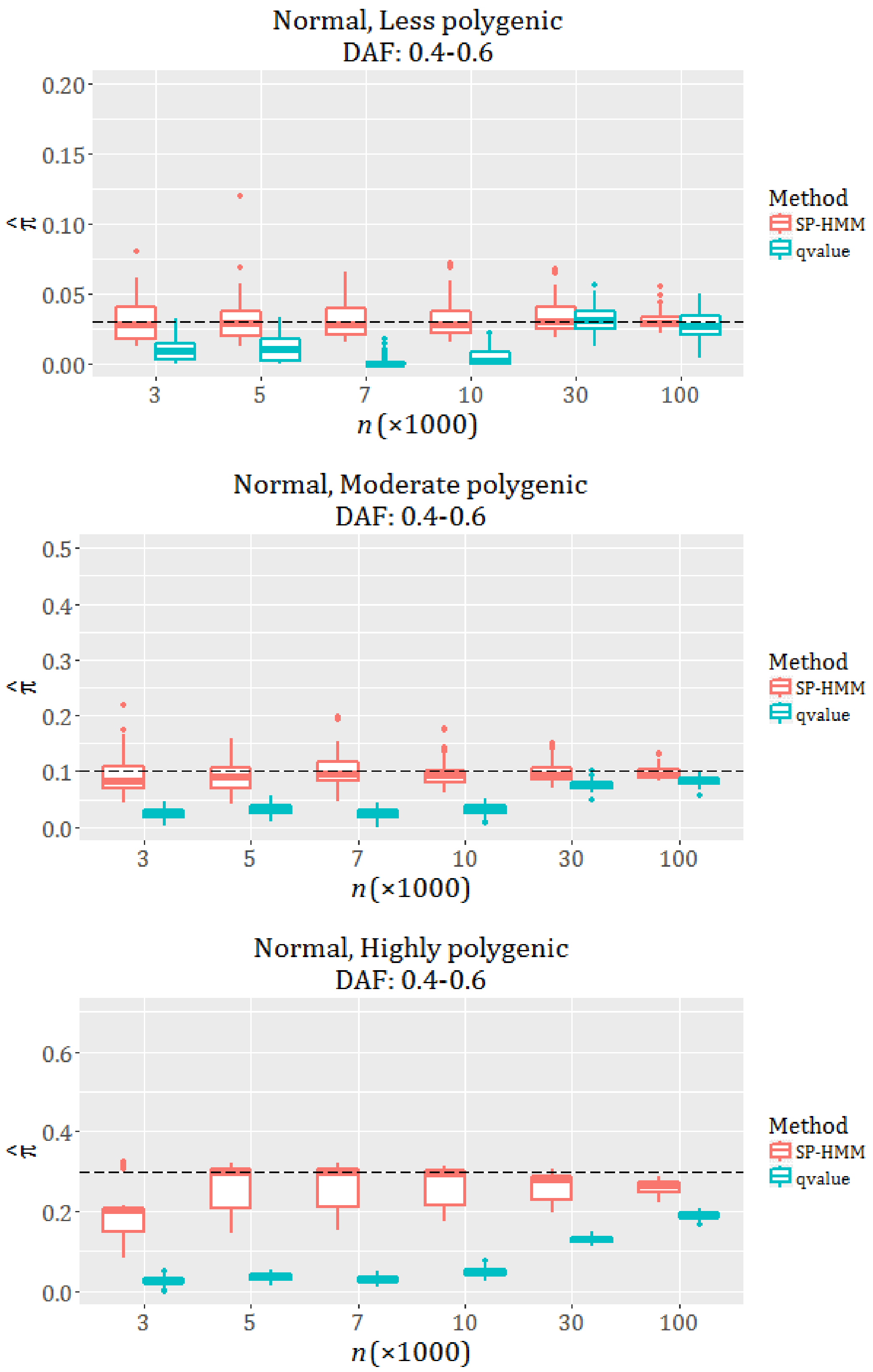


Figure S4. (Continued).

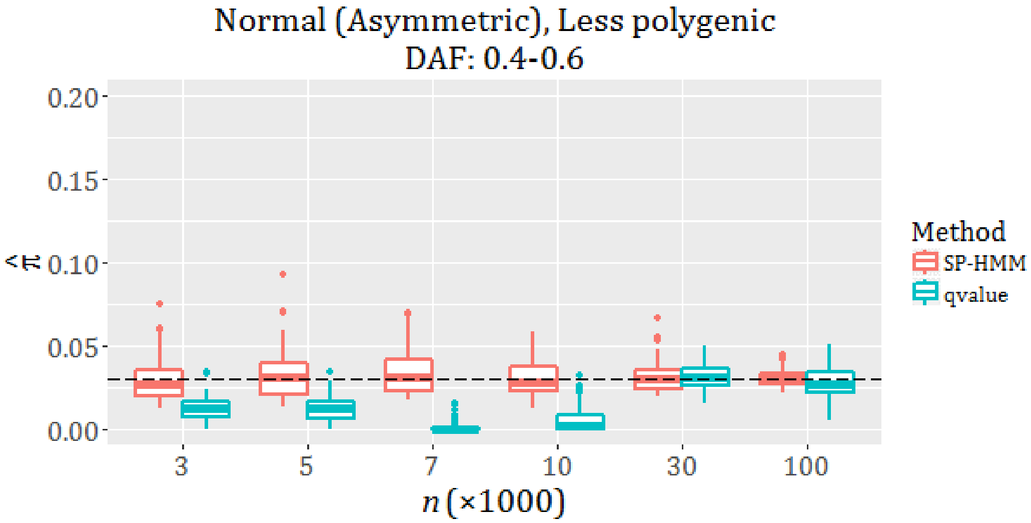


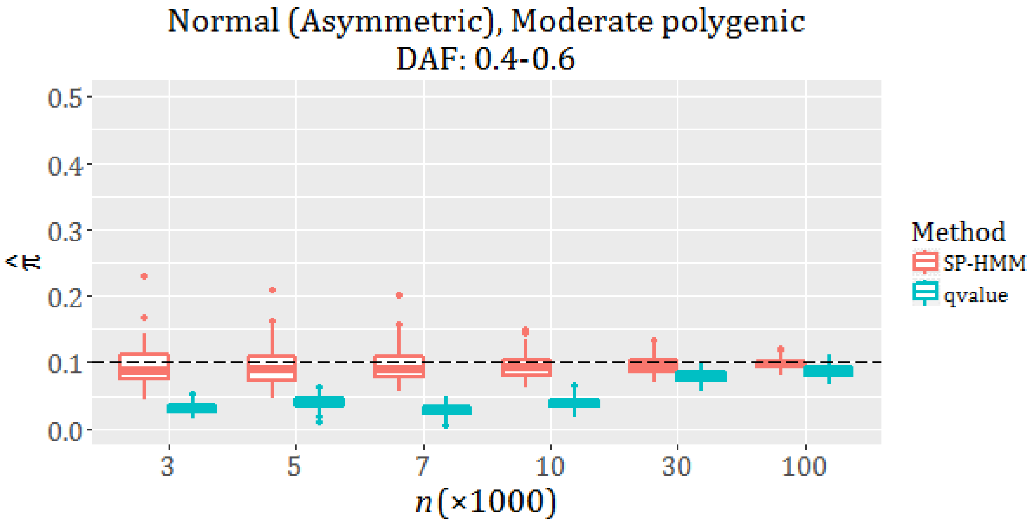


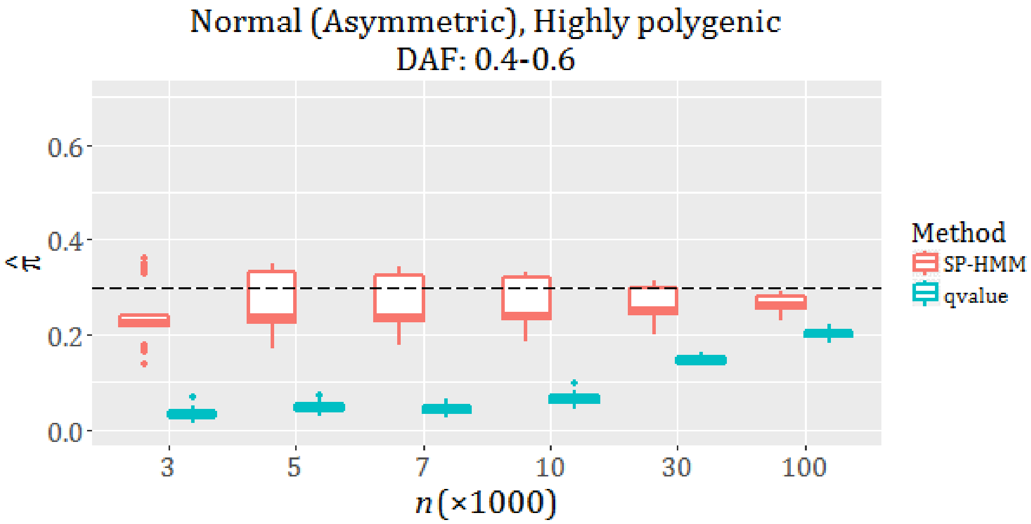


Figure S4. (Continued).

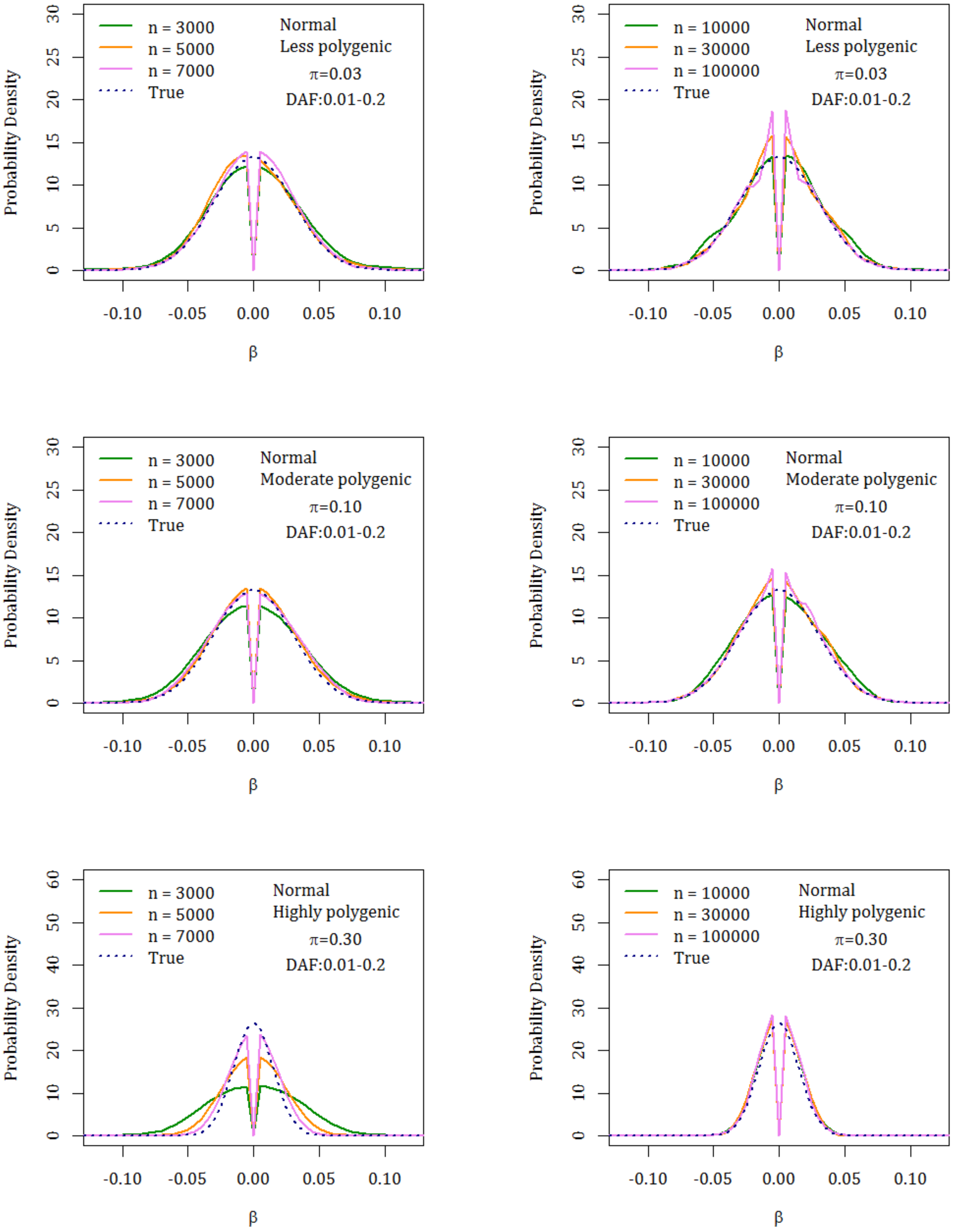


Figure S5. Estimated effect-size distributions, $\hat{g}$, in the DAF-stratified analysis. Average curves over 100 simulations for each sample size are shown. The specified (true) effect-size distributions are given in dotted lines.

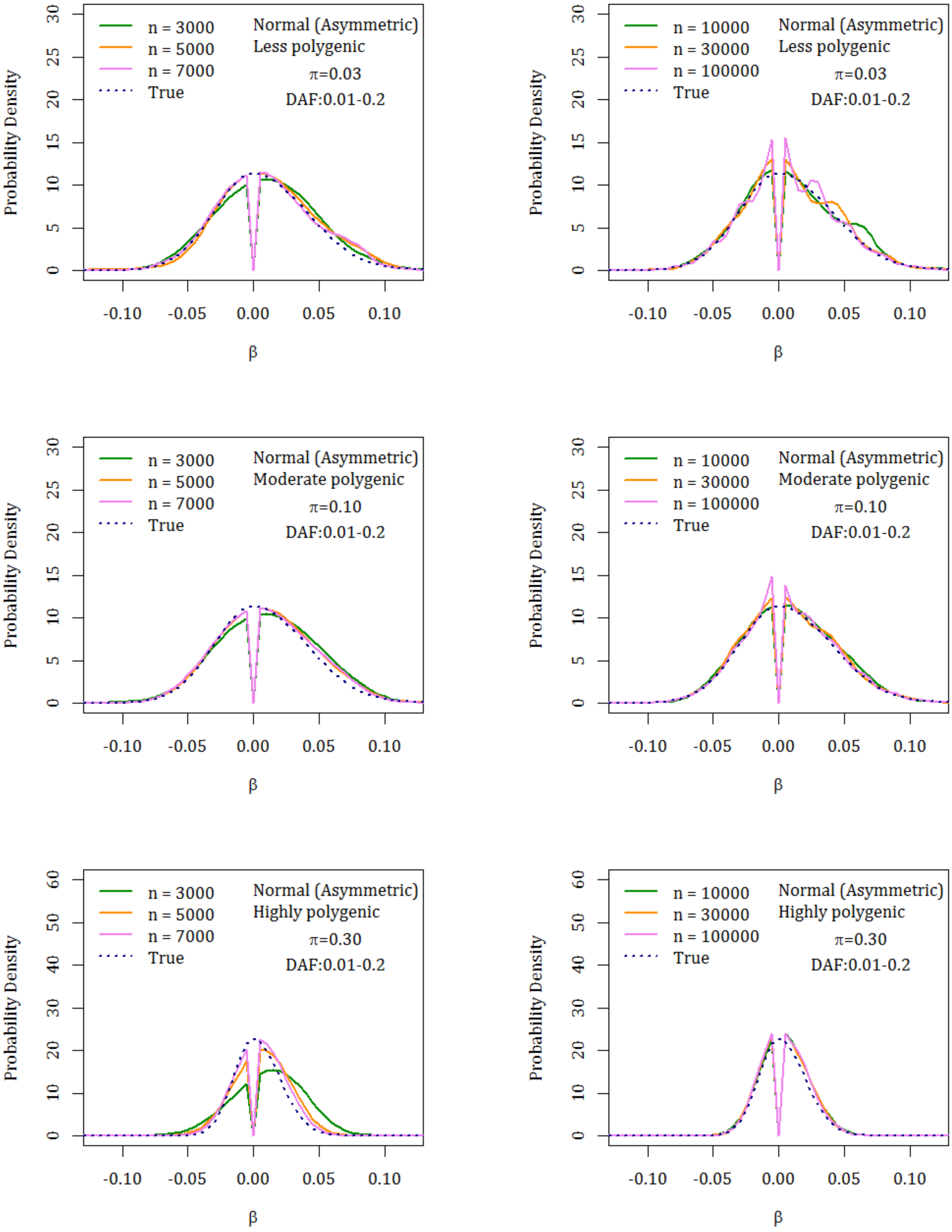


Figure S5. (Continued).

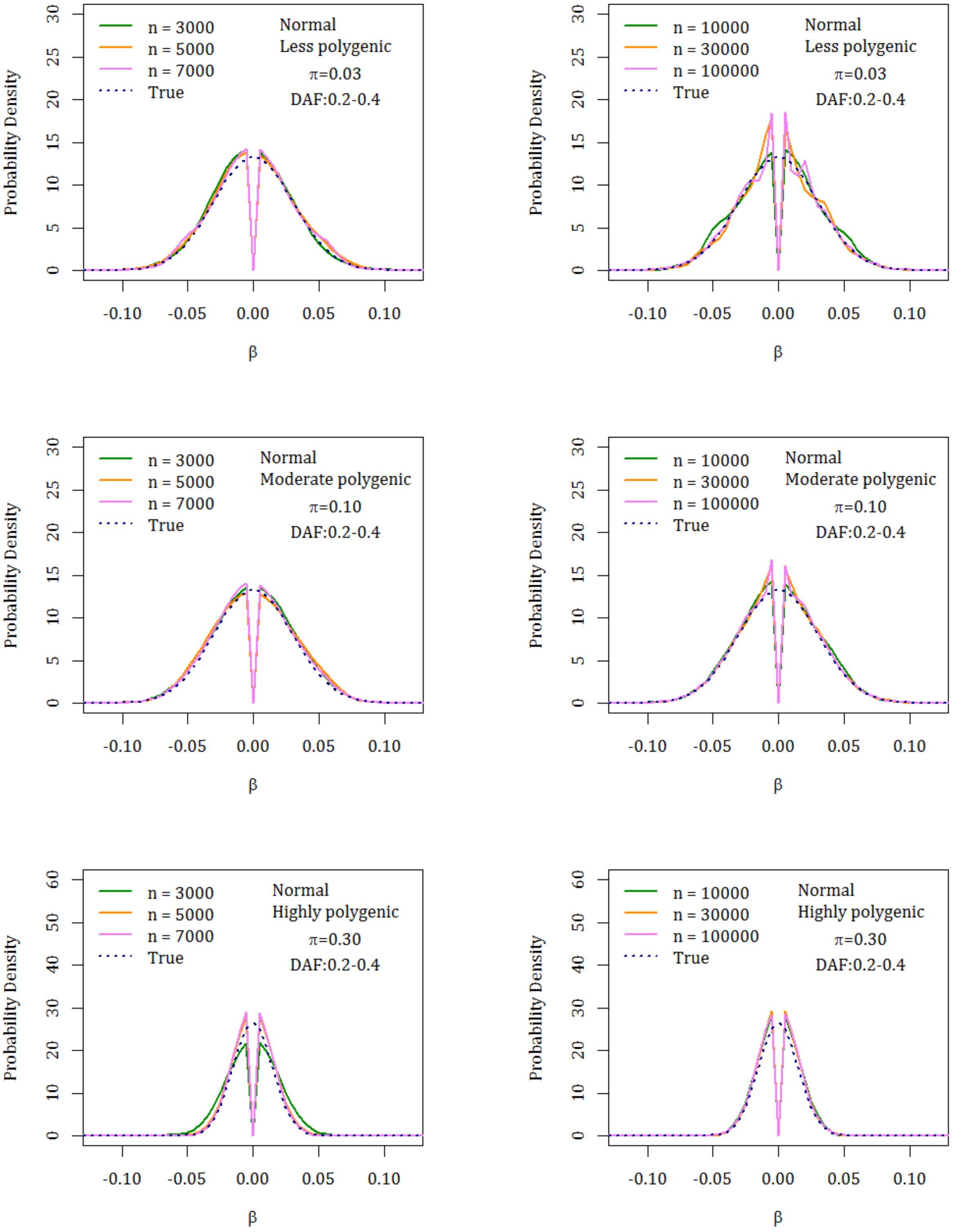


Figure S5. (Continued).

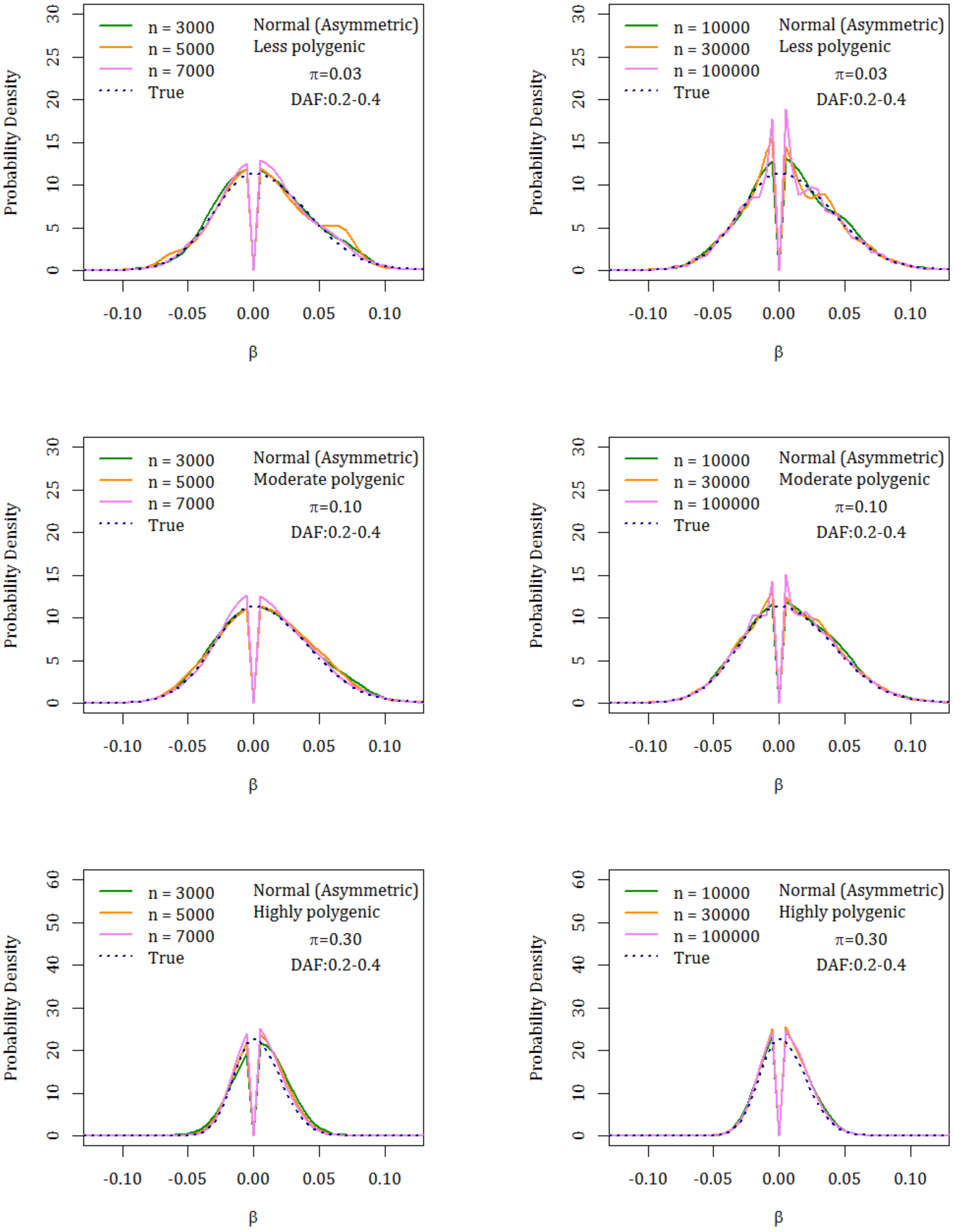


Figure S5. (Continued).

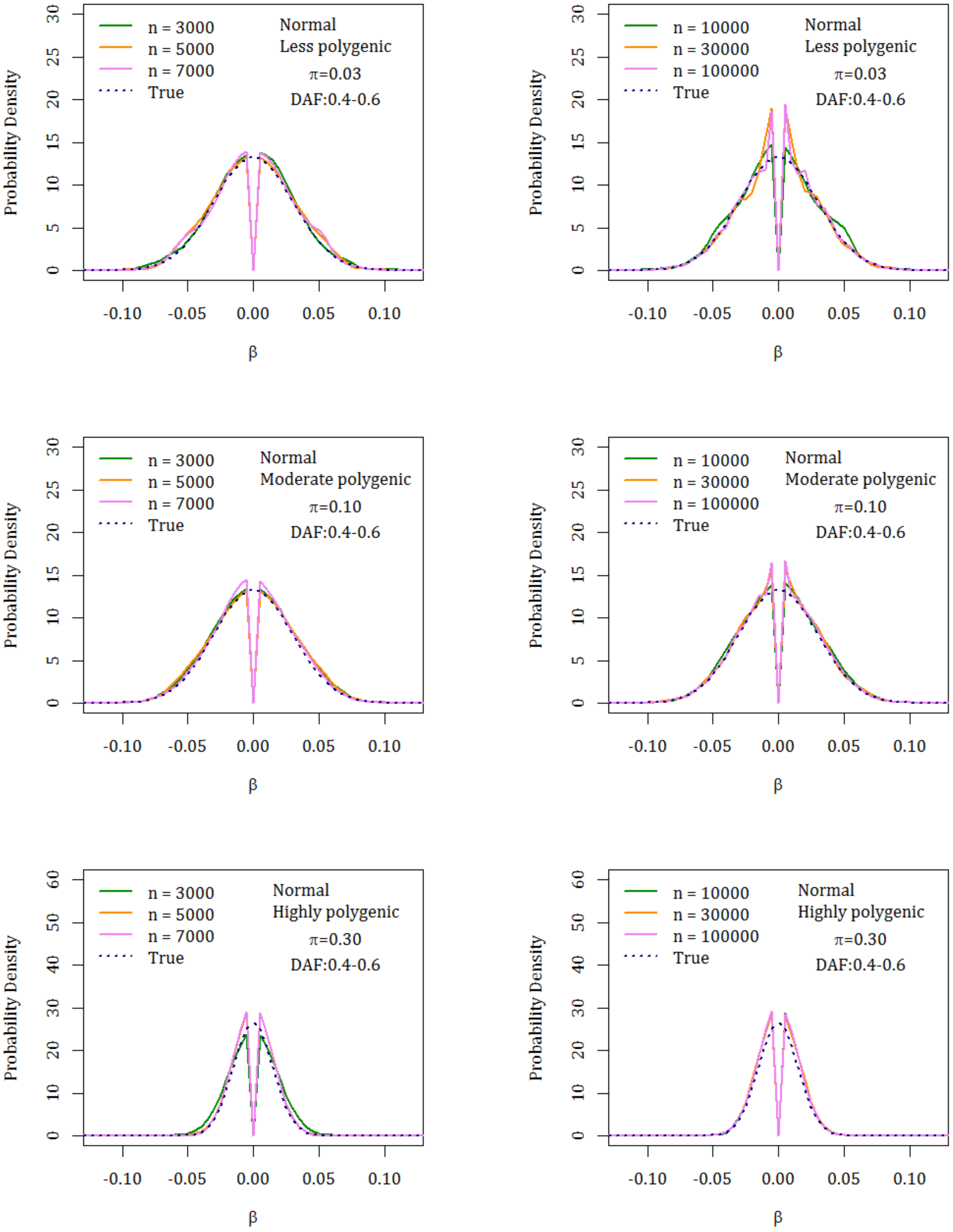


Figure S5. (Continued).

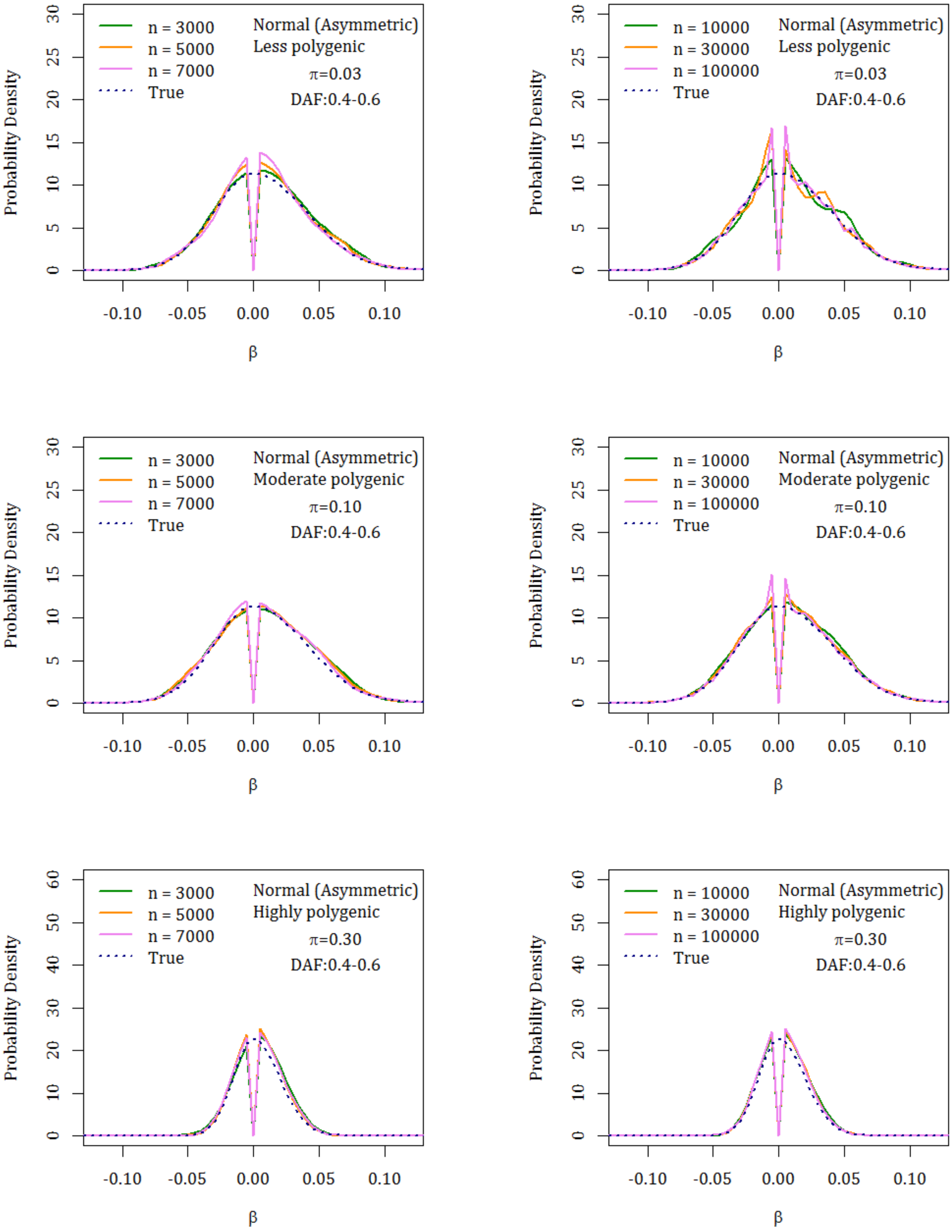


Figure S5. (Continued).

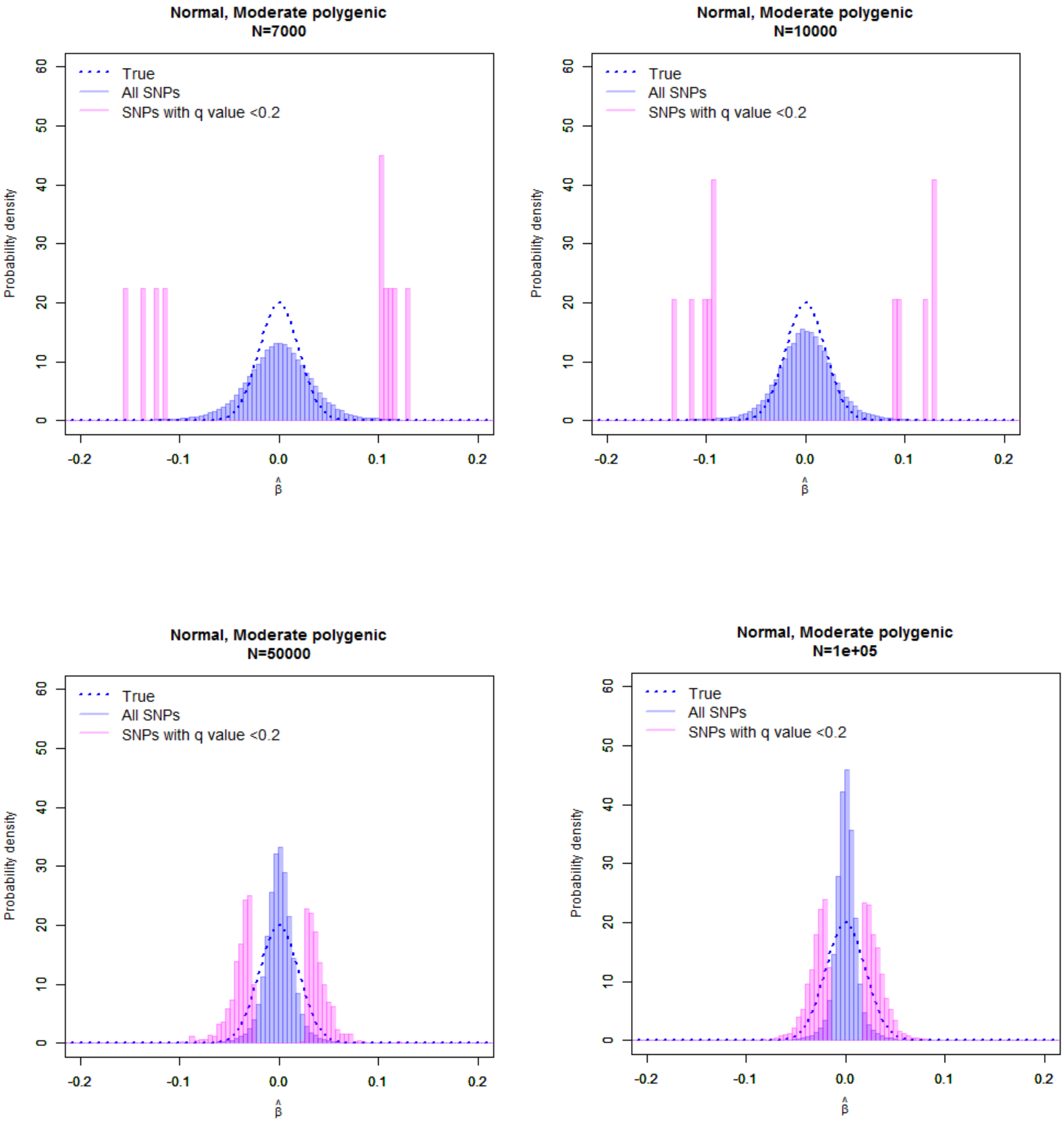


Figure S6. Raw effect size estimates, $\hat{\beta}$, for simulated data in the settings using normal distributions ($m$=100000). DAF's, were generated from a uniform distribution U(0.01, 0.99). Each histogram is a result of one simulation replicate. The specified (true) effect-size distributions are given in dotted lines.

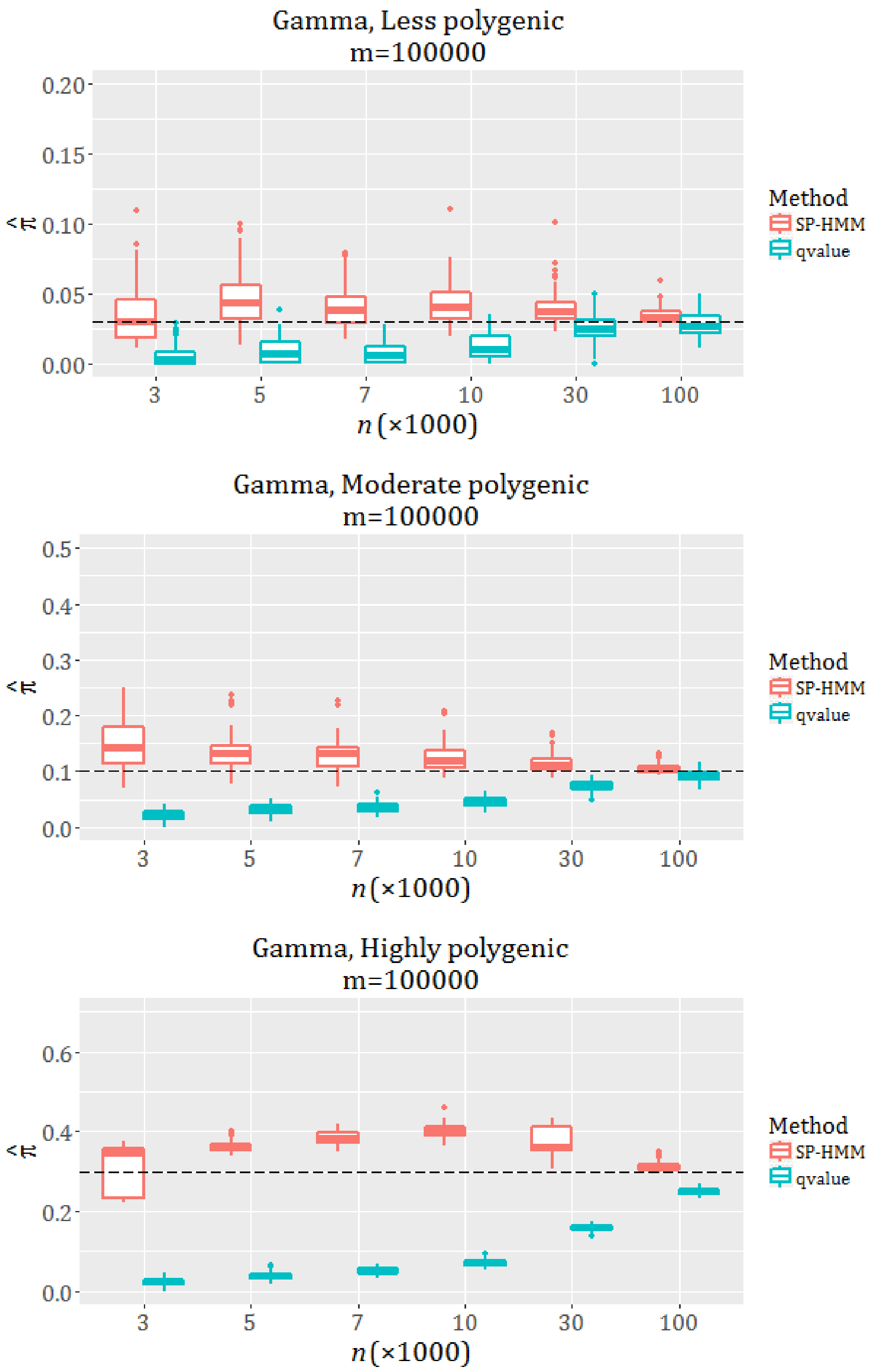


Figure S7. Estimated proportions of disease-associated SNPs, $\hat{\pi}$, in the settings using gamma distributions ($m$=100000). Each box plot shows 100 simulation replicates. The black dashed lines show the specified (true) $\pi$.

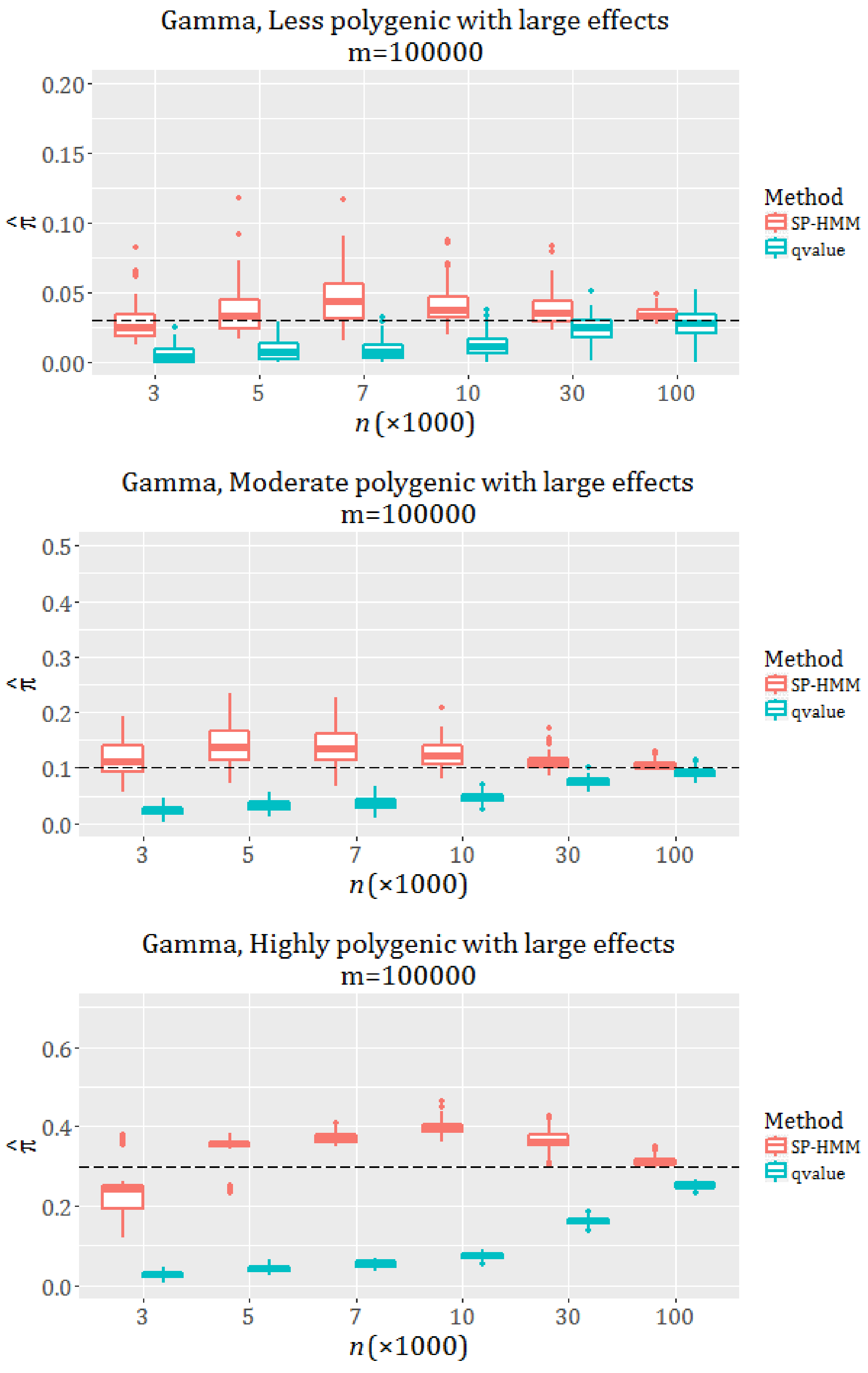


Figure S7. (Continued).

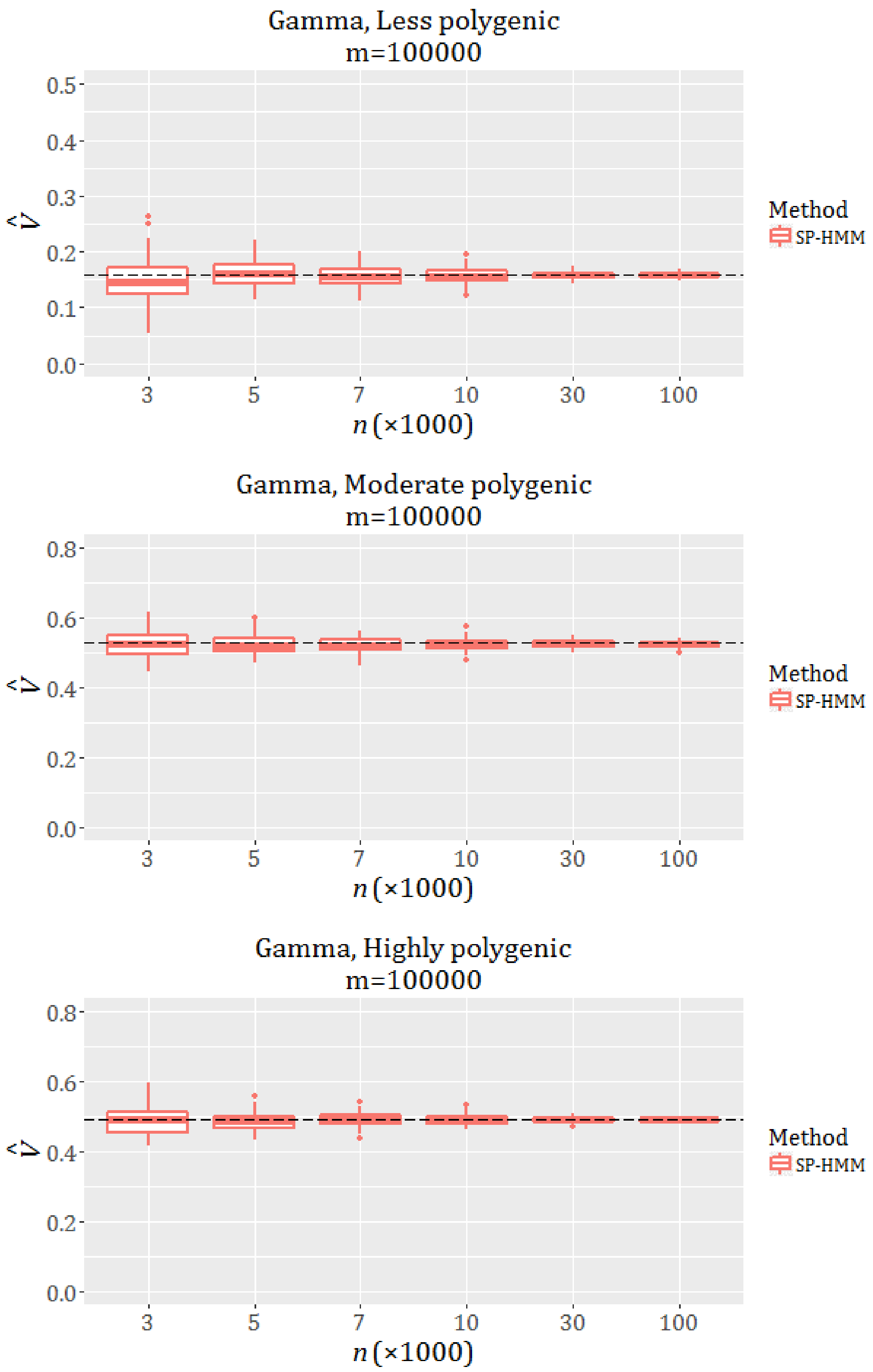


Figure S8. Estimated liability-scale variance, $\hat{V}$, in the settings using gamma distributions ($m$=100000). Each box plot shows 100 simulation replicates. The black dashed lines show the specified (true) $V$.

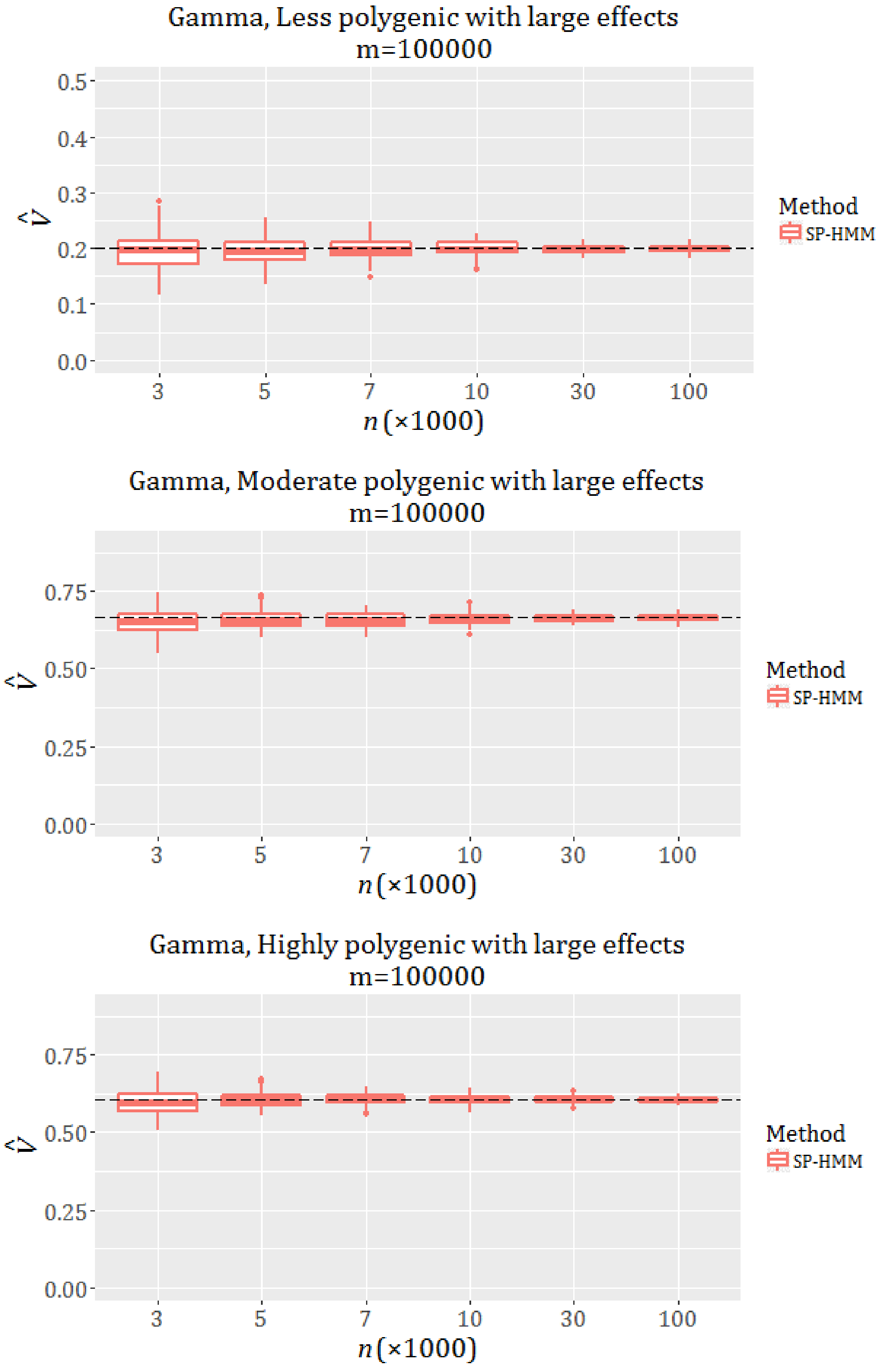


Figure S8. (Continued).

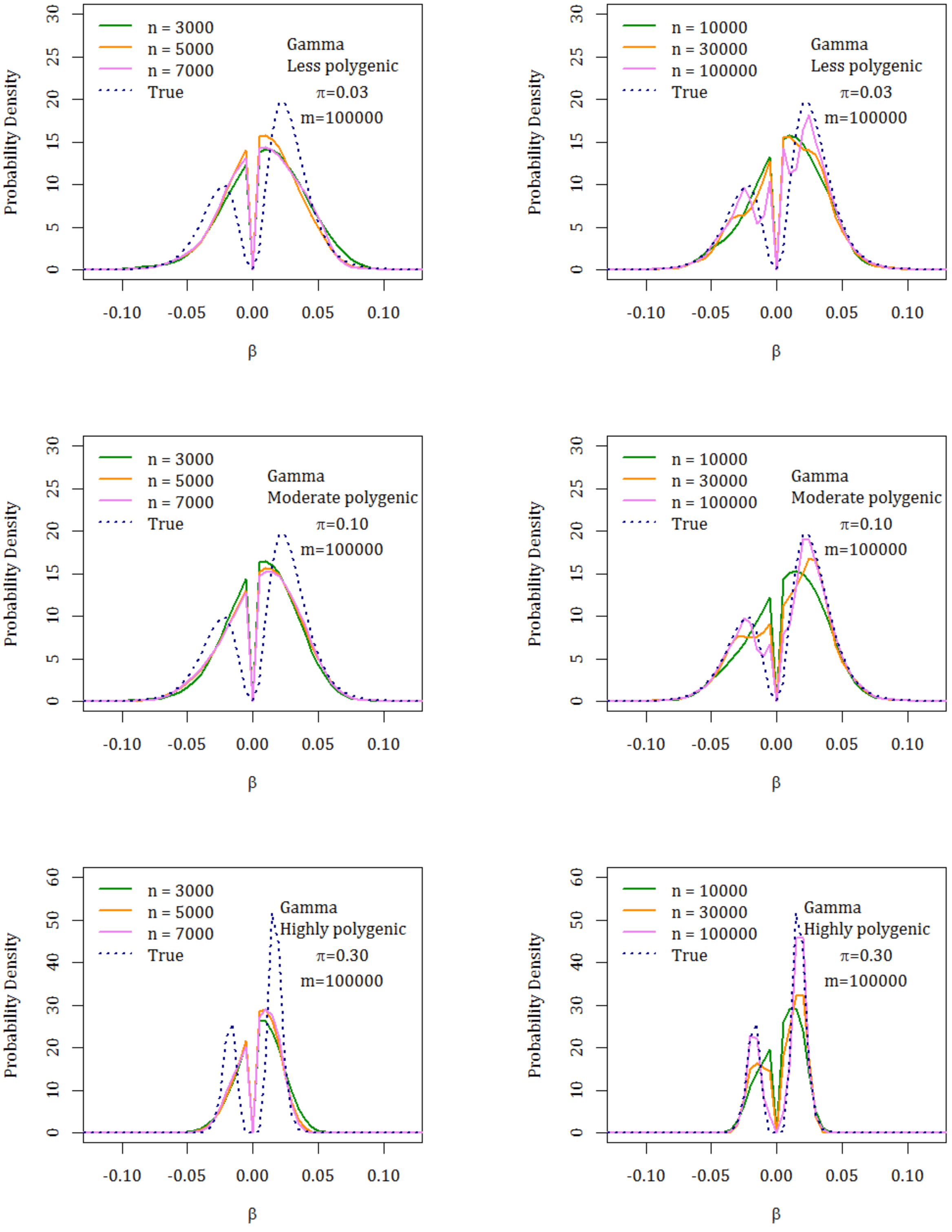


Figure S9. Estimated effect-size distributions, $\hat{g}$, in the settings using gamma distributions ($m$=100000). Average curves over 100 simulations for each sample size are shown. The specified (true) effect-size distributions are given in dotted lines.

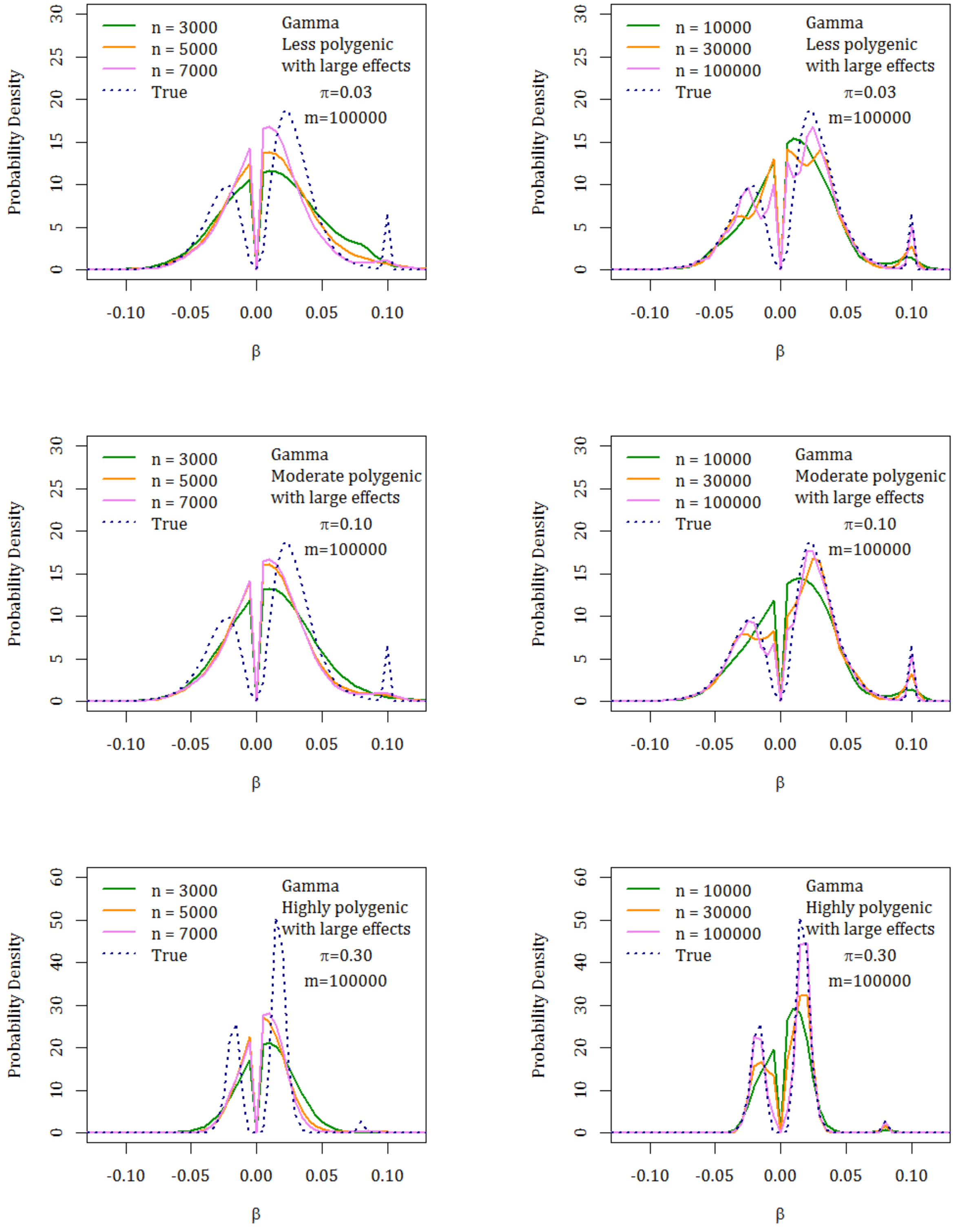


Figure S9. (Continued).

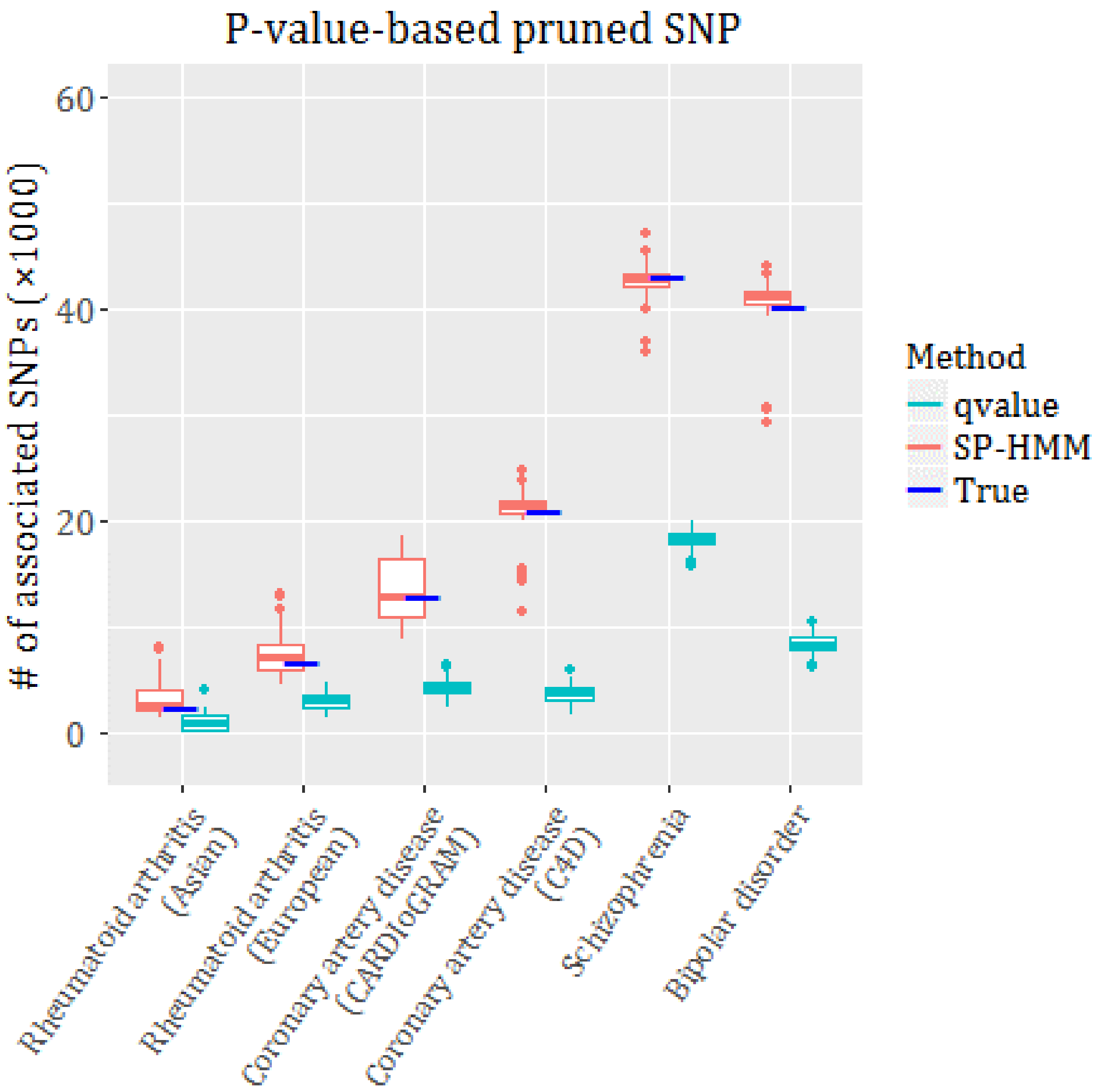


Figure S10. Estimated proportions of disease-associated SNPs, $\hat{\pi}$, under the estimated genetic architectures from the P-value-based pruned SNPs. Each box plot shows 100 simulation replicates. The black segment lines show the specified (true) values determined by the estimated architectures, $\hat{\pi} \times m$, for each GWAS dataset.

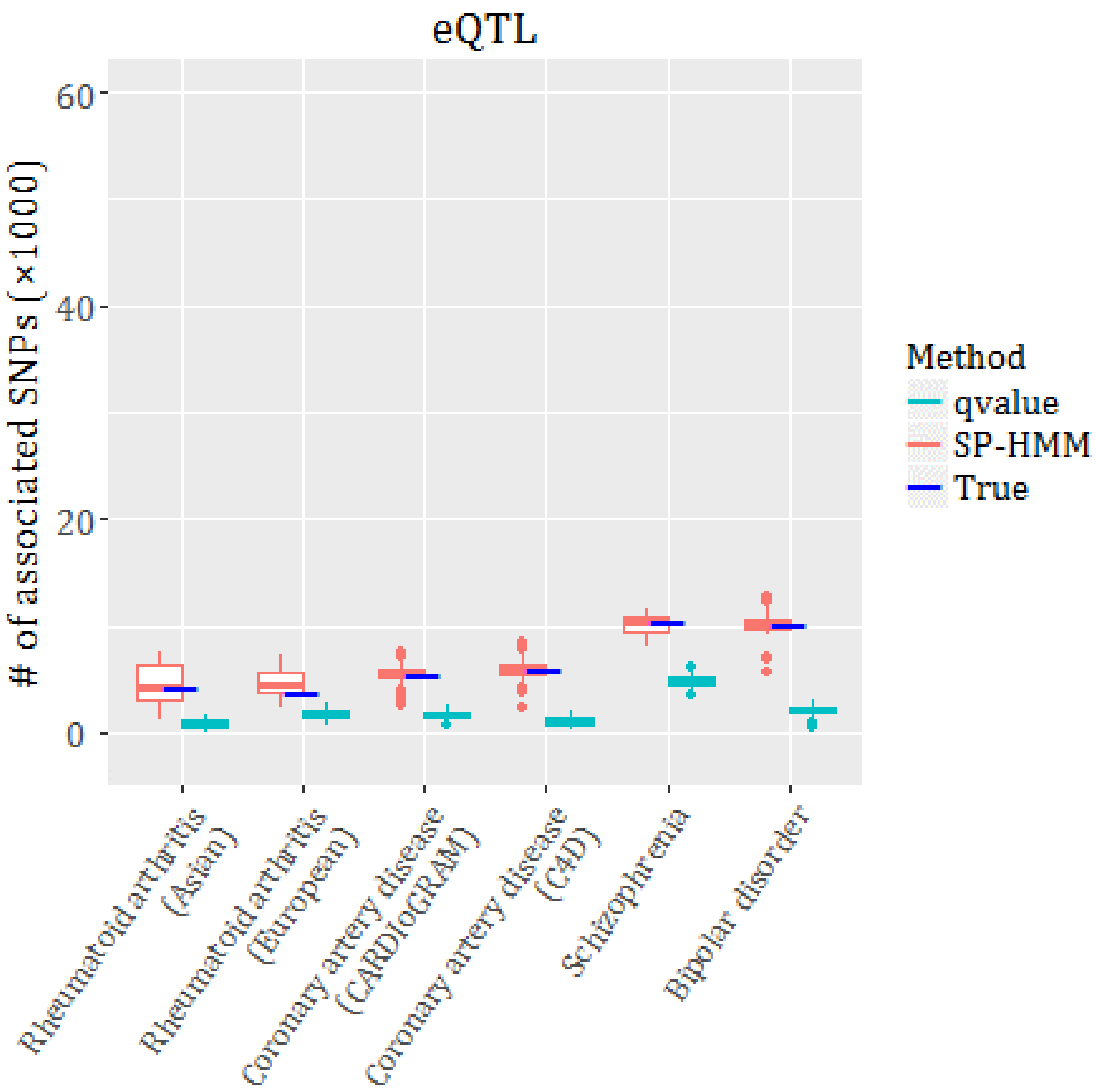


Figure S11. Estimated proportions of disease-associated SNPs, $\hat{\pi}$, under the estimated genetic architectures from the eQTL/not eQTL SNPs. Each box plot shows 100 simulation replicates. The black segment lines show the specified (true) values determined by the estimated architectures, $\hat{\pi} \times m$, for each GWAS data.

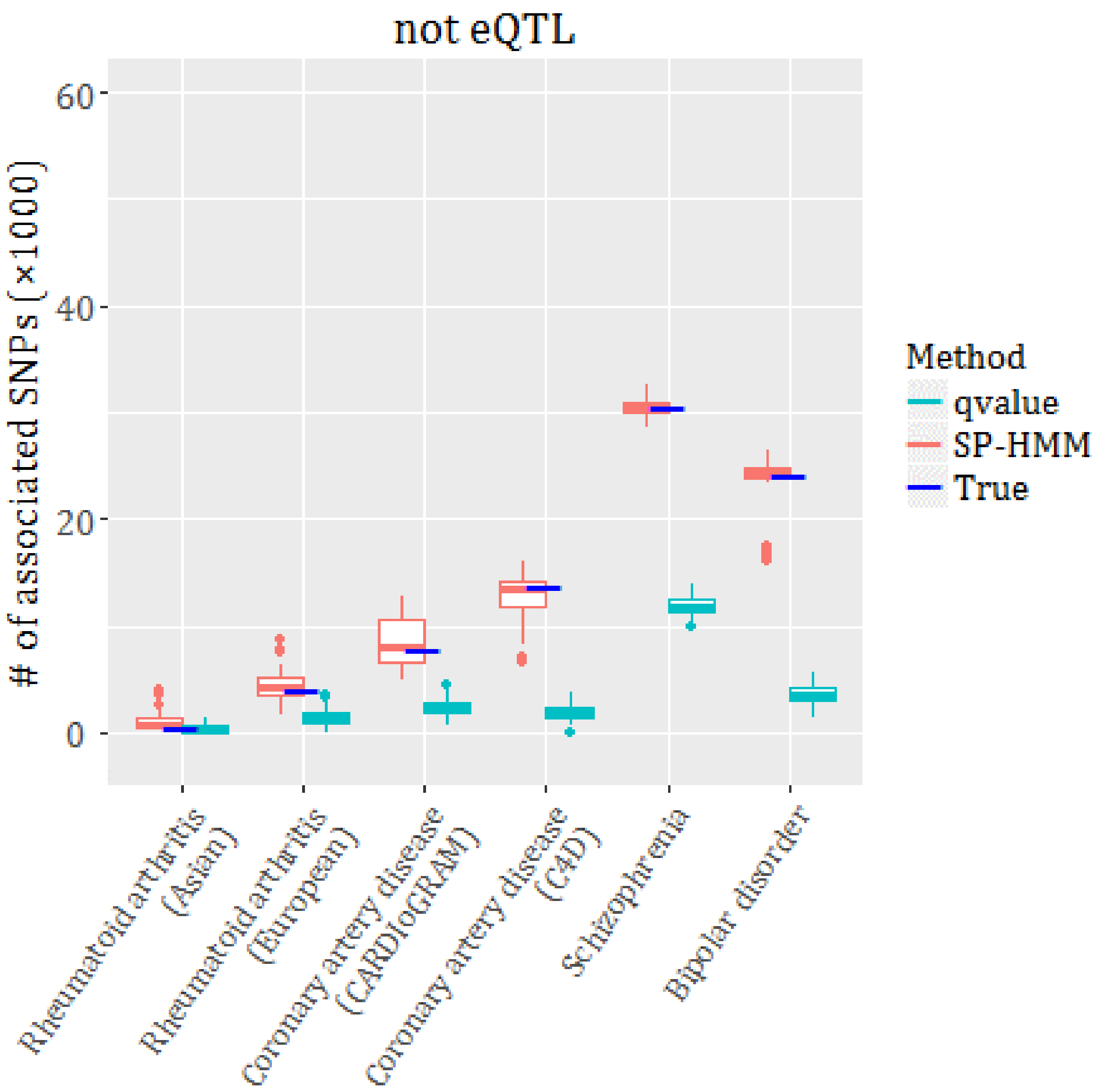


Figure S11. (Continued).

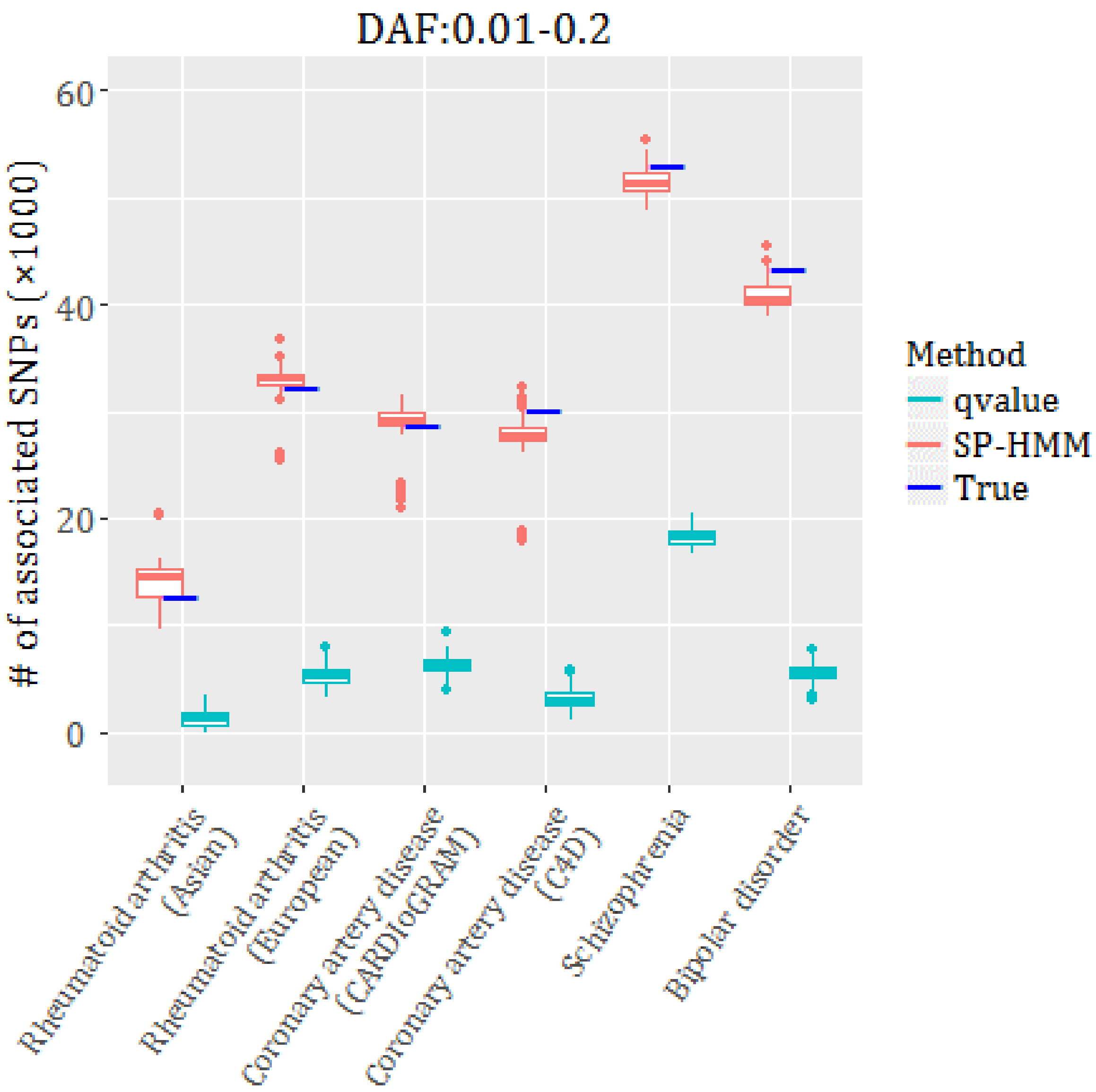


Figure S12. Estimated proportions of disease-associated SNPs, $\hat{\pi}$, under the estimated genetic architectures from the DAF stratified SNPs. Each box plot shows 100 simulation replicates. The black segment lines show the specified (true) values determined by the estimated architectures, $\hat{\pi} \times m$, for each GWAS dataset. For each DAF bin, we used 100,000 SNPs with the exception that in C4D GWAS the numbers of SNPs in $0.4 < \text{DAF} \leq 0.6$, $0.6 < \text{DAF} \leq 0.8$, and $0.8 < \text{DAF}$ were 94506, 70170, and 49116, respectively, since the SNPs of C4D GWAS was limited (Table S2).

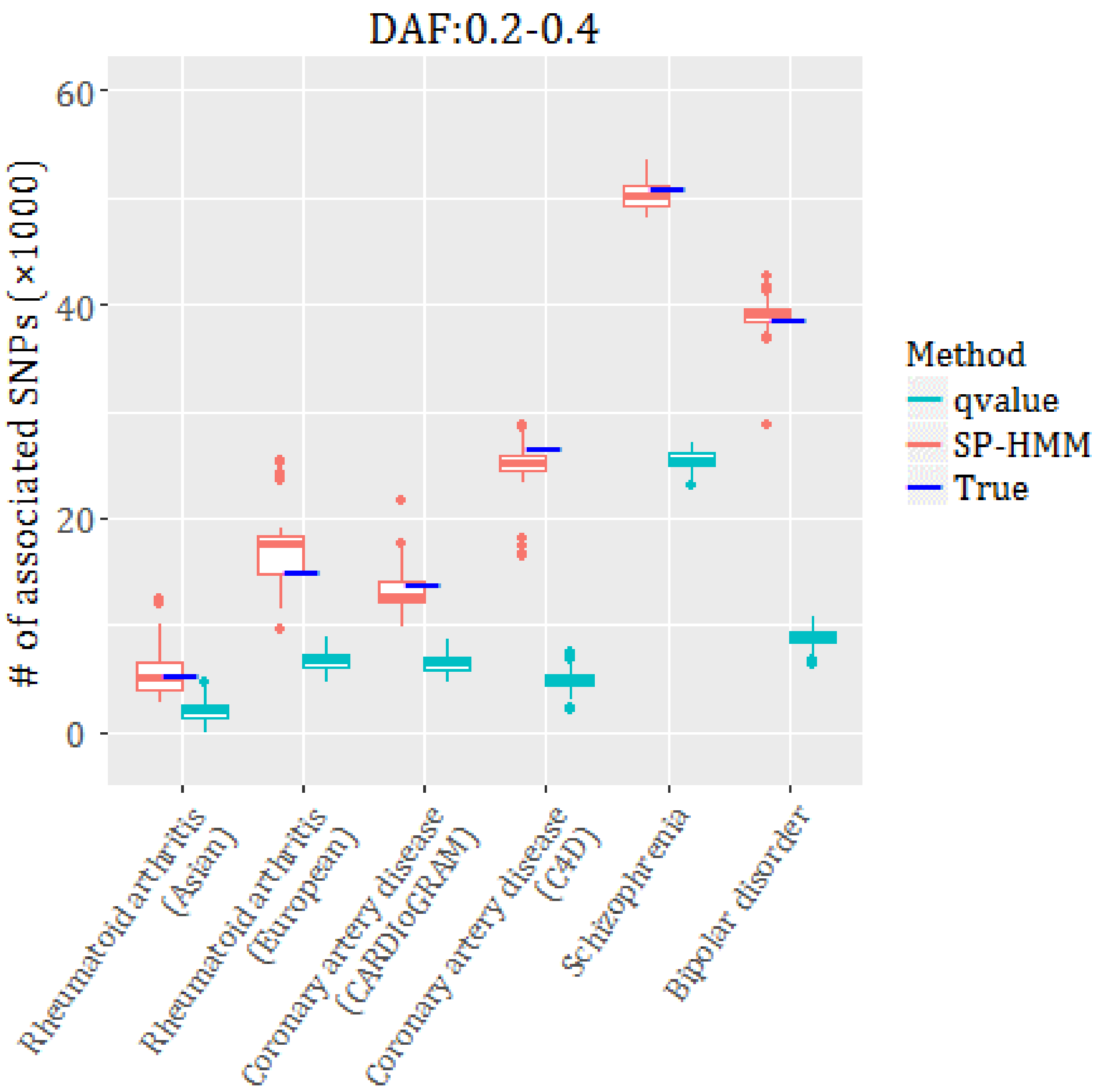


Figure S12. (Continued).

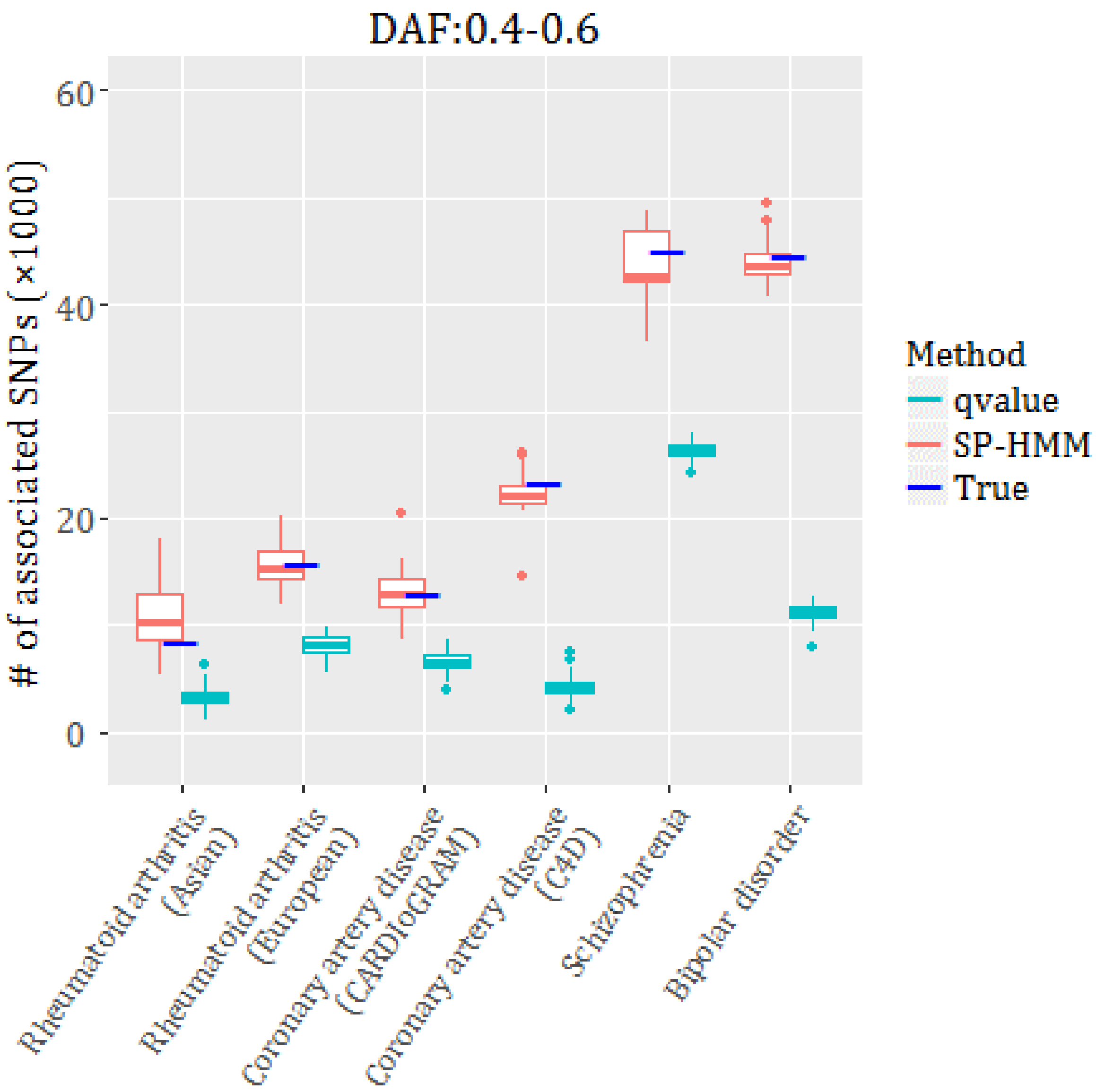


Figure S12. (Continued).

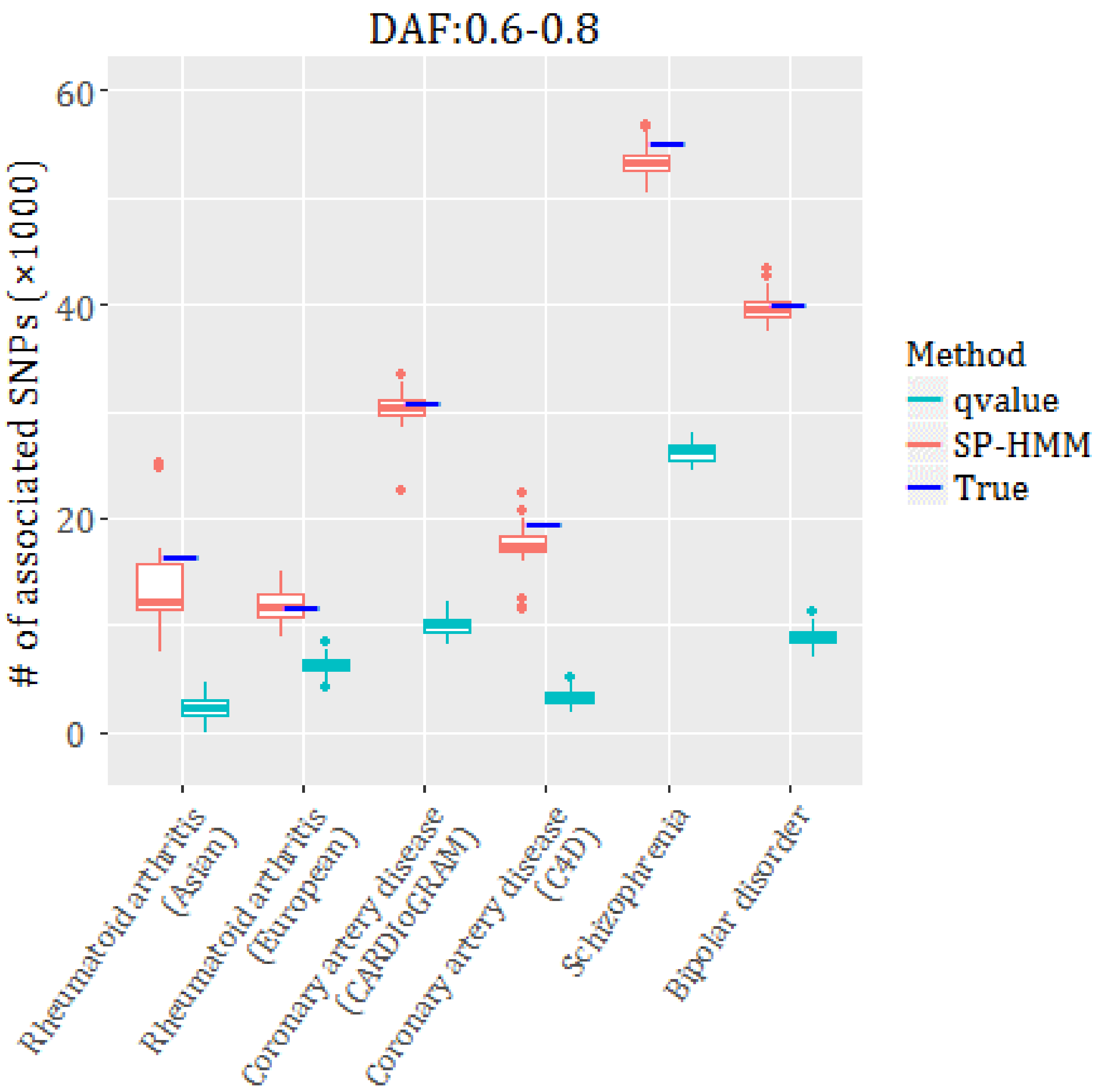


Figure S12. (Continued).

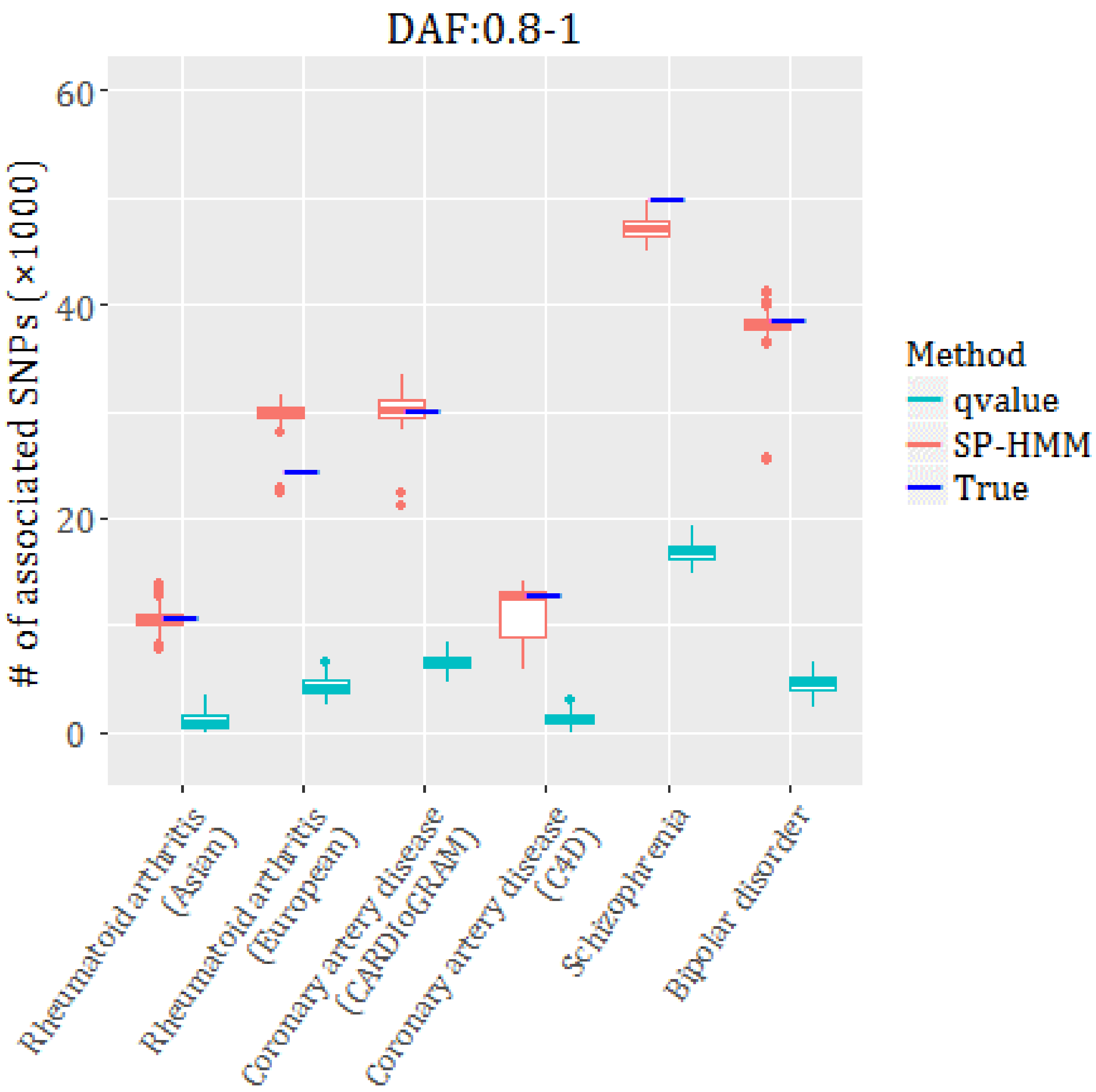


Figure S12. (Continued).

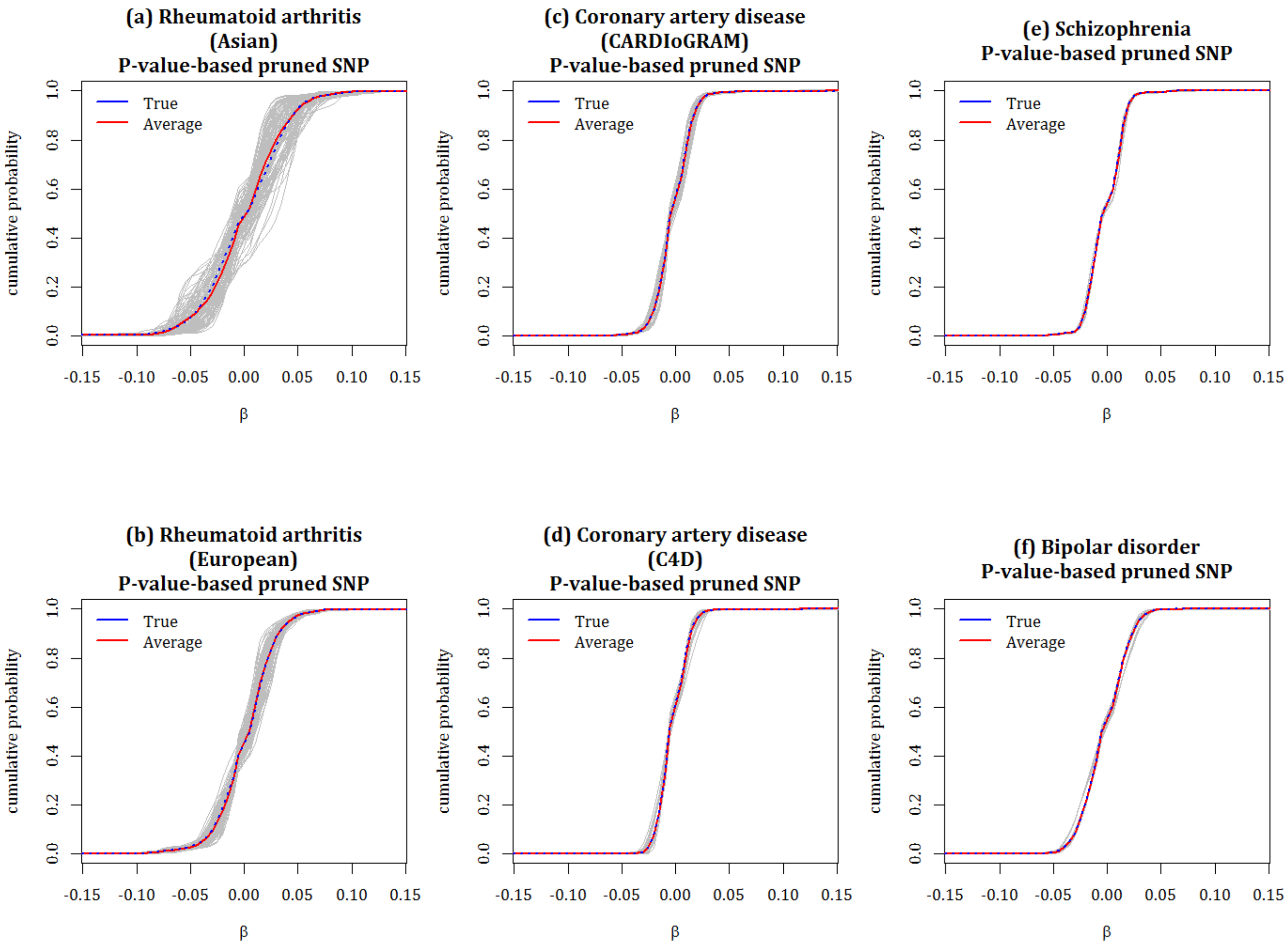


Figure S13. Estimated effect-size distributions, $\hat{g}$, under the estimated genetic architectures from the P-value-based pruned SNPs. The estimated curves for 100 simulations (gray lines) and their average curves (red solid lines) are shown. The specified (true) effect-size distributions are also given (blue solid lines).

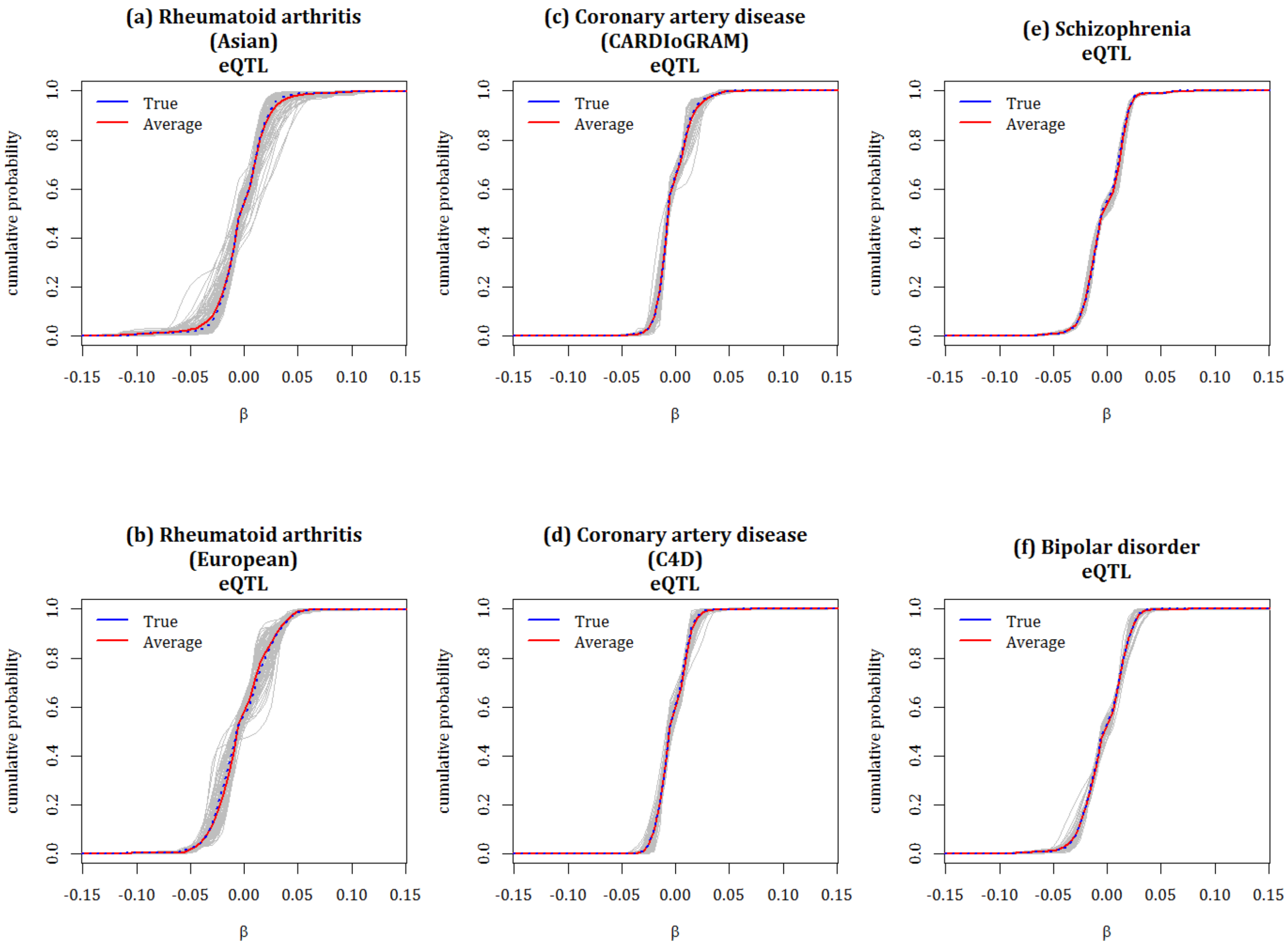


Figure S14. Estimated effect-size distributions, $\hat{g}$, under the estimated genetic architectures from the eQTL/not eQTL SNPs. The estimated curves for 100 simulations (gray lines) and their average curves (red solid lines) are shown. The specified (true) effect-size distributions are also given (blue solid lines).

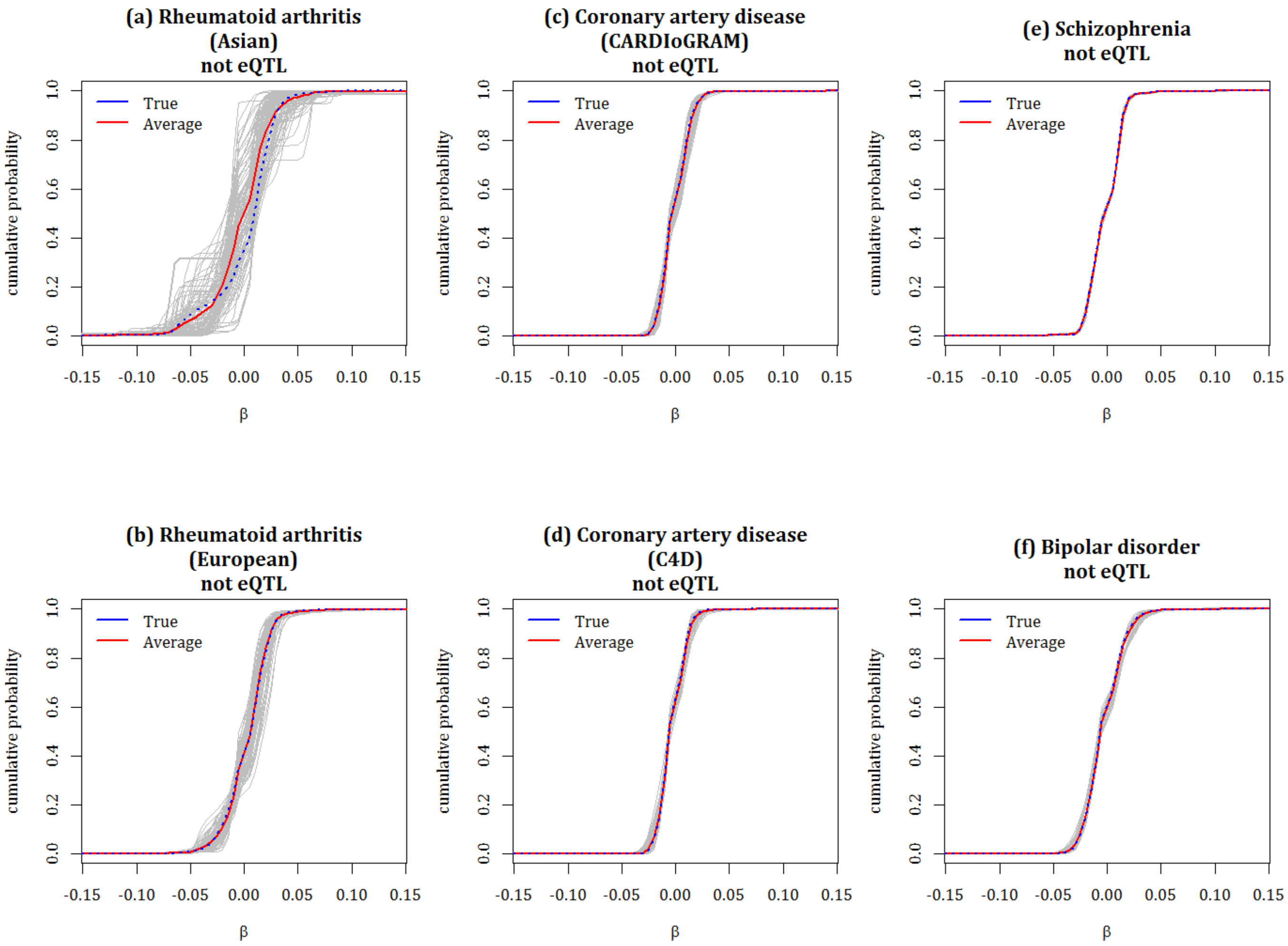


Figure S14. (Continued).

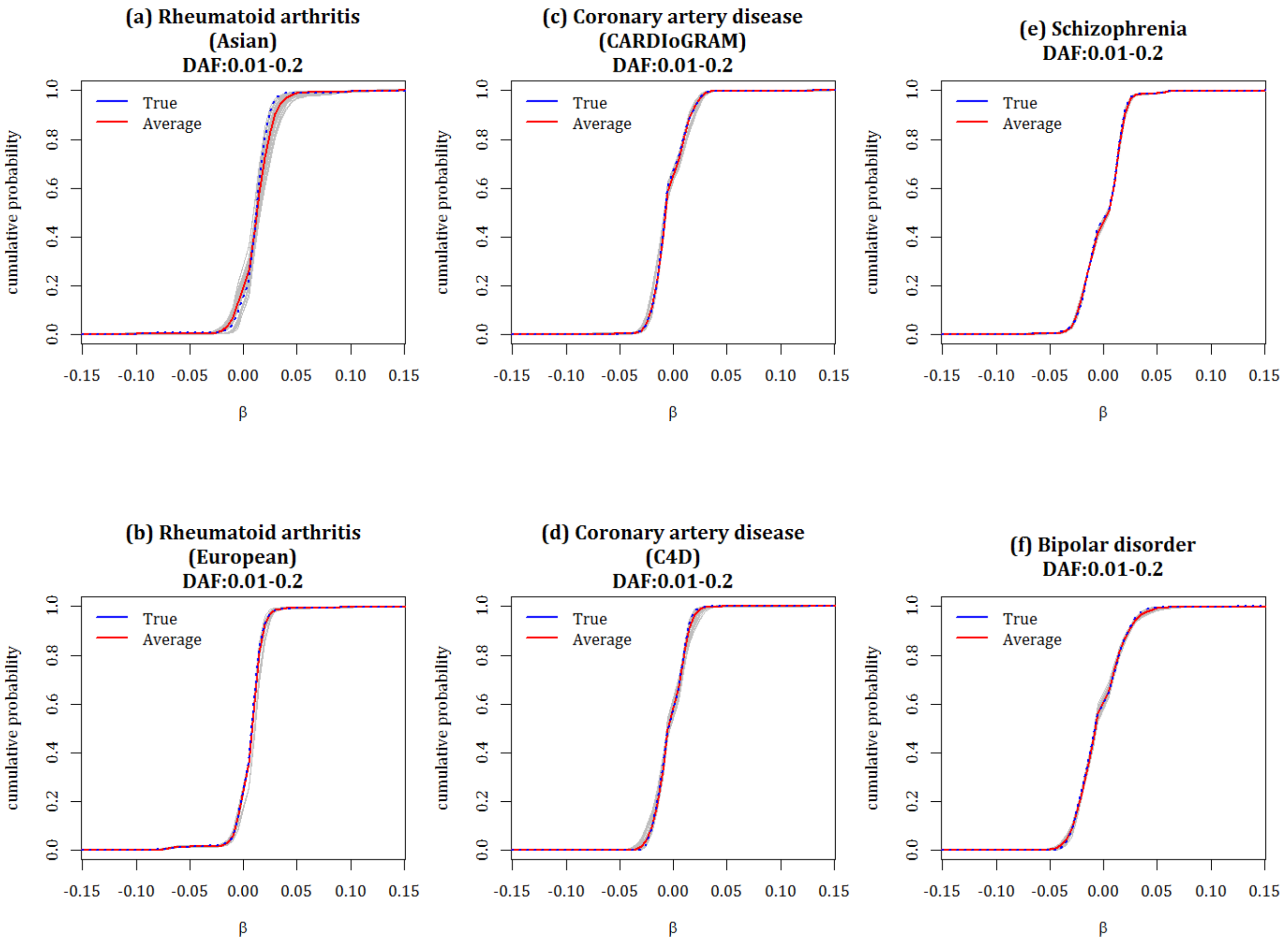


Figure S15. Estimated effect-size distributions, $\hat{g}$, under the estimated genetic architectures from the DAF stratified SNPs. The estimated curves for 100 simulations (gray lines) and their average curves (red solid lines) are shown. The specified (true) effect-size distributions are also given (blue solid lines). For each DAF bin, we used 100,000 SNPs with the exception that in C4D GWAS the numbers of SNPs in $0.4 < \text{DAF} \leq 0.6$, $0.6 < \text{DAF} \leq 0.8$, and $0.8 < \text{DAF}$ were 94506, 70170, and 49116, respectively, since the SNPs of C4D GWAS was limited (Table S2).

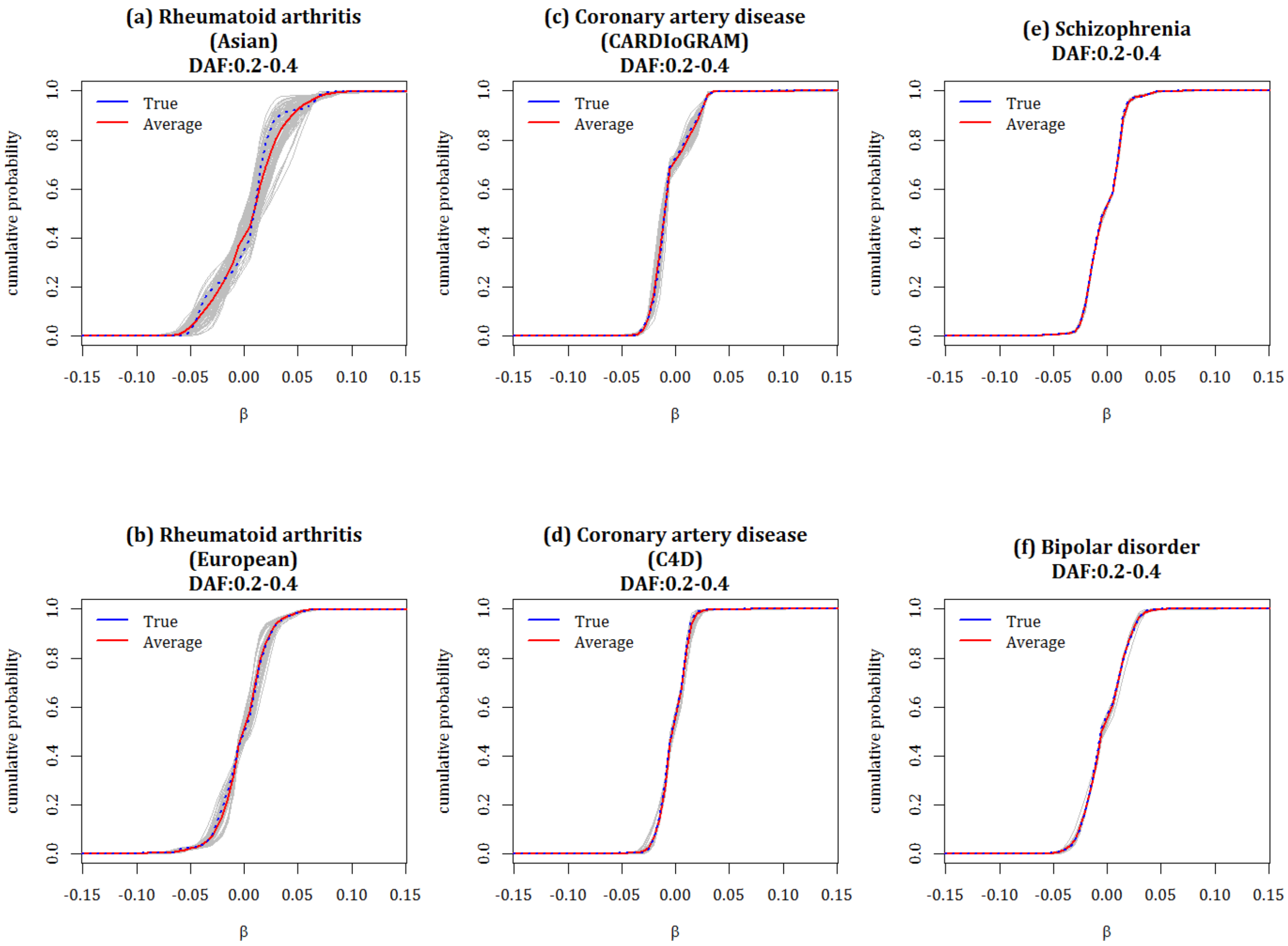


Figure S15. (Continued).

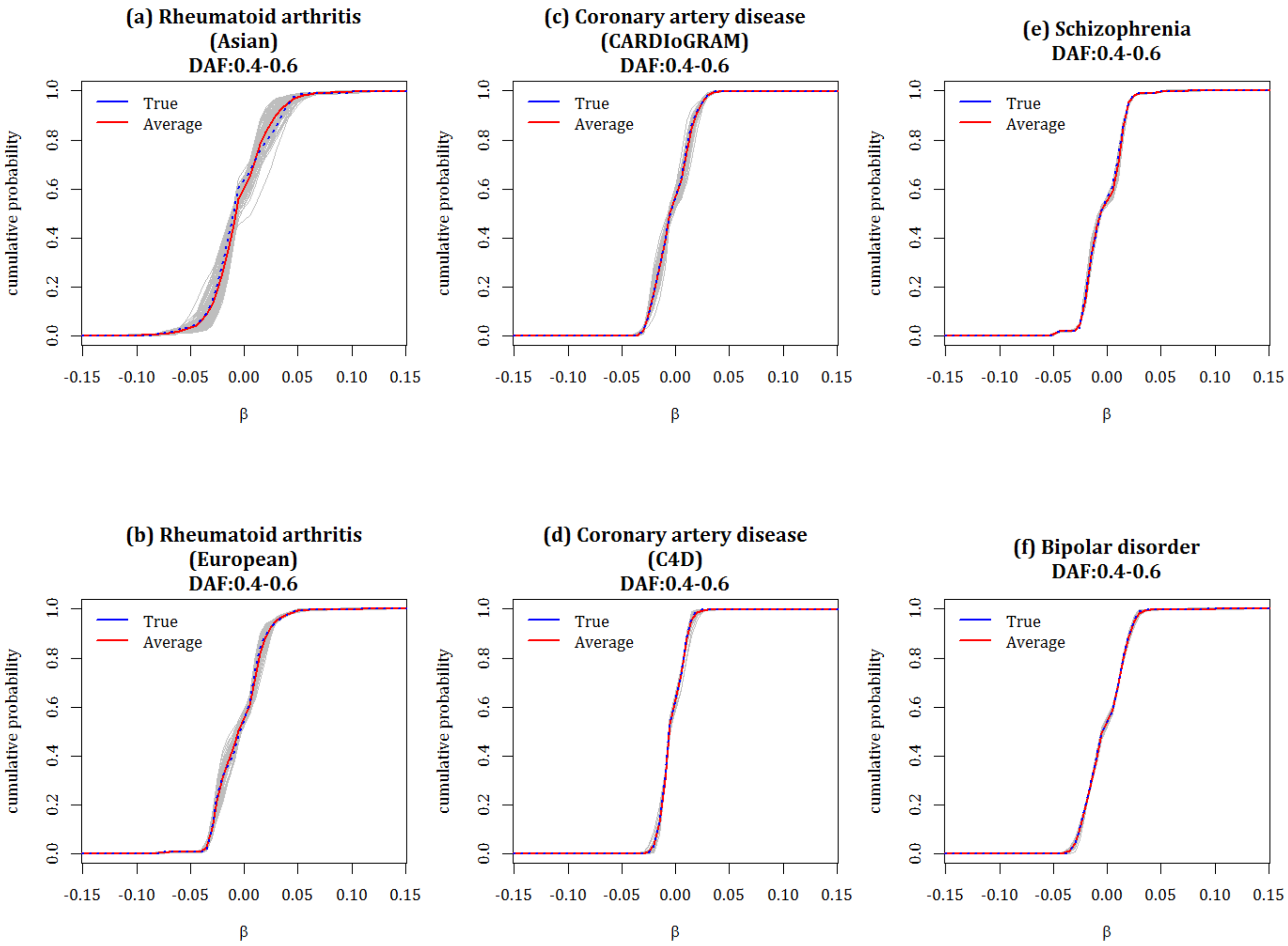


Figure S15. (Continued).

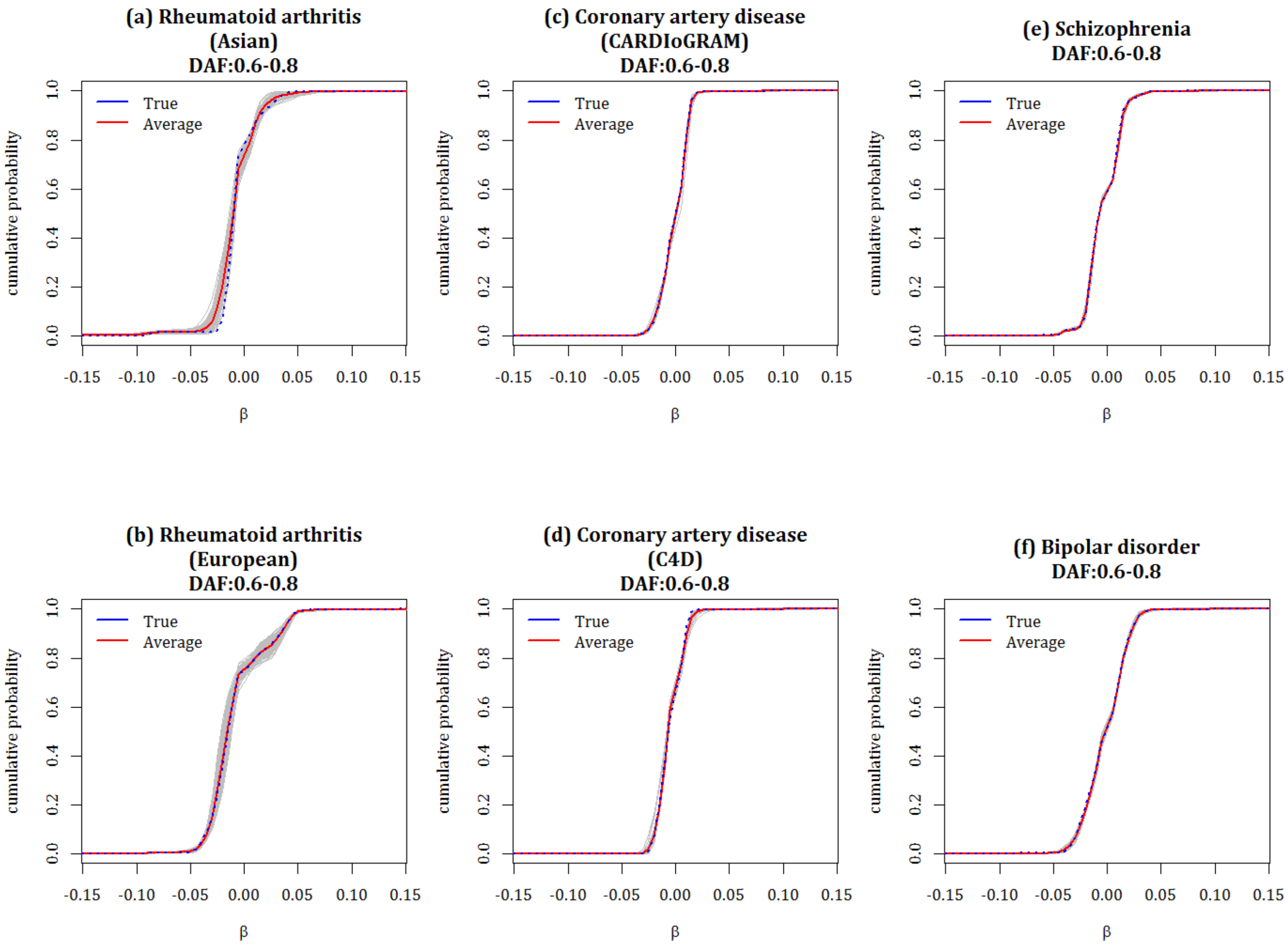


Figure S15. (Continued).

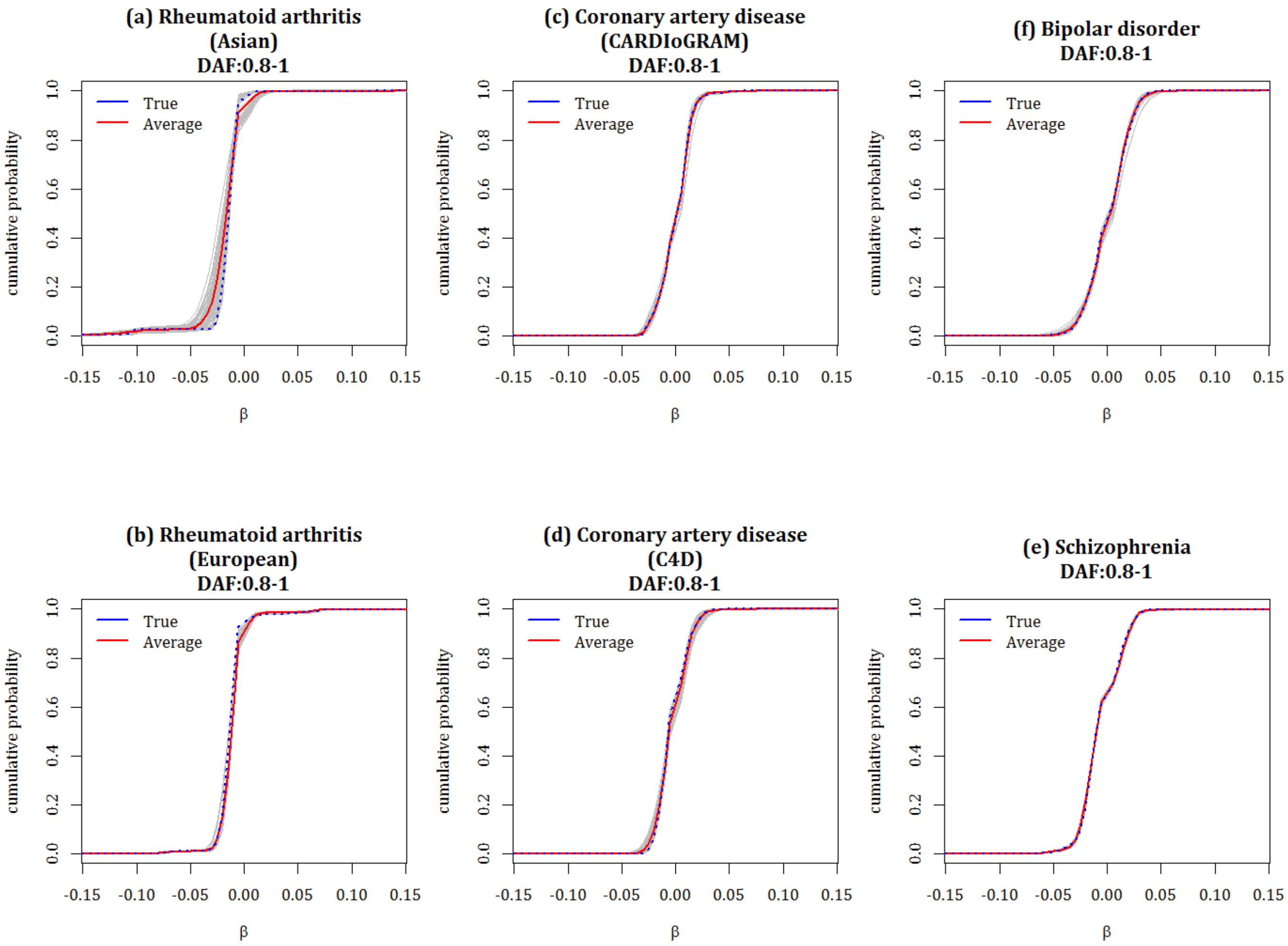


Figure S15. (Continued).

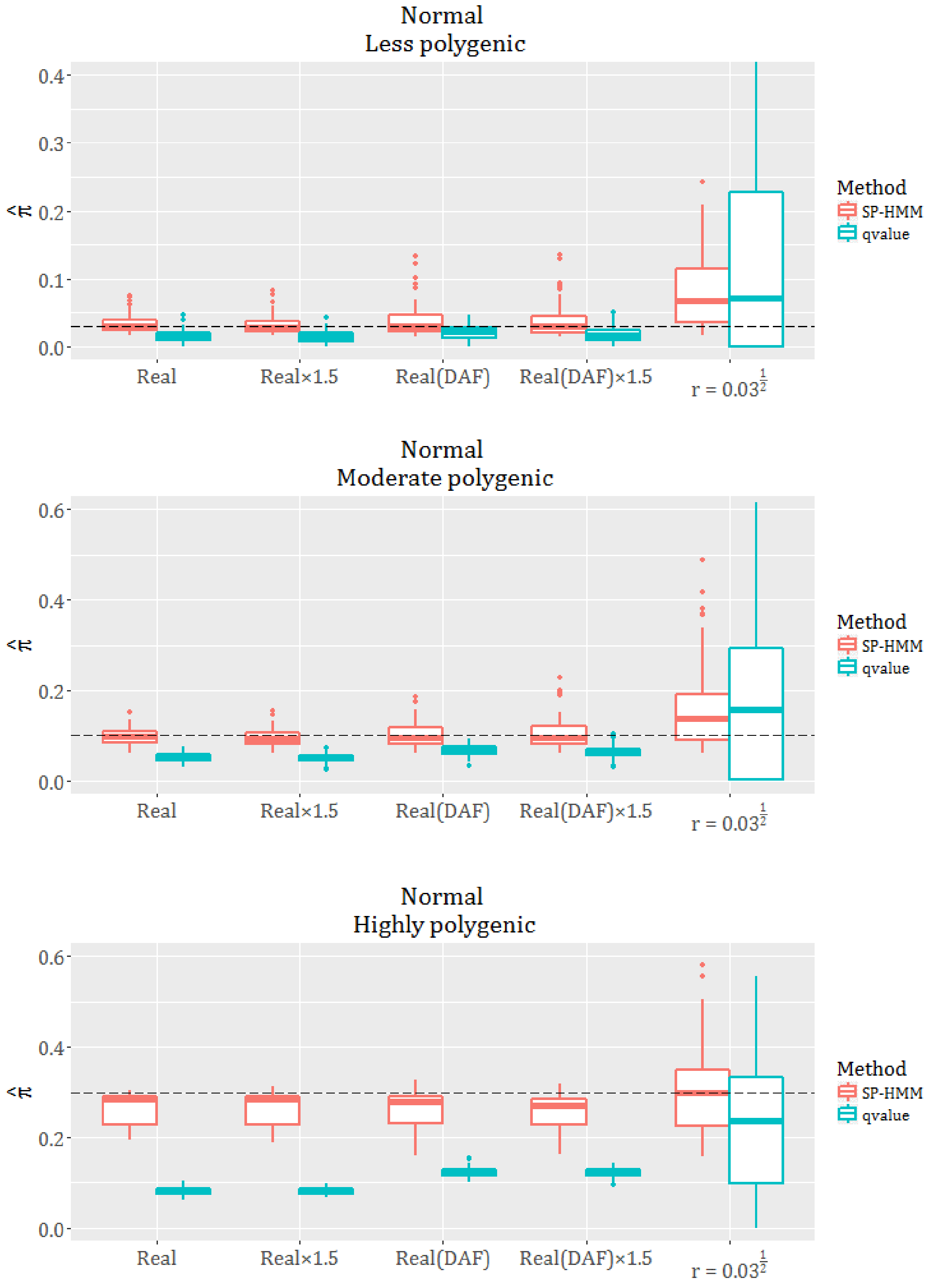


Figure S16. Estimated proportions of disease-associated SNPs, $\hat{\pi}$, in the settings with SNPs in weak LD. Each box plot shows 100 simulation replicates. The black dashed lines show the specified (true) $\pi$. 'Real': Incorporating LD of the P-value based pruned set. 'Real × 1.5': Incorporating 1.5 times stronger LD(r) than that of the P-value based pruned set. 'Real (DAF)': Incorporating LD of the SNP set with $0.4 \leq \text{DAF} < 0.6$ for DAF stratified analysis. 'Real (DAF) × 1.5': Incorporating 1.5 times stronger LD(r) than that of the SNP set with $0.4 \leq \text{DAF} < 0.6$ for DAF stratified analysis. '$r = 0.03^{1/2}$': Incorporating constant LD among all SNP pairs($r = 0.03^{1/2}$).

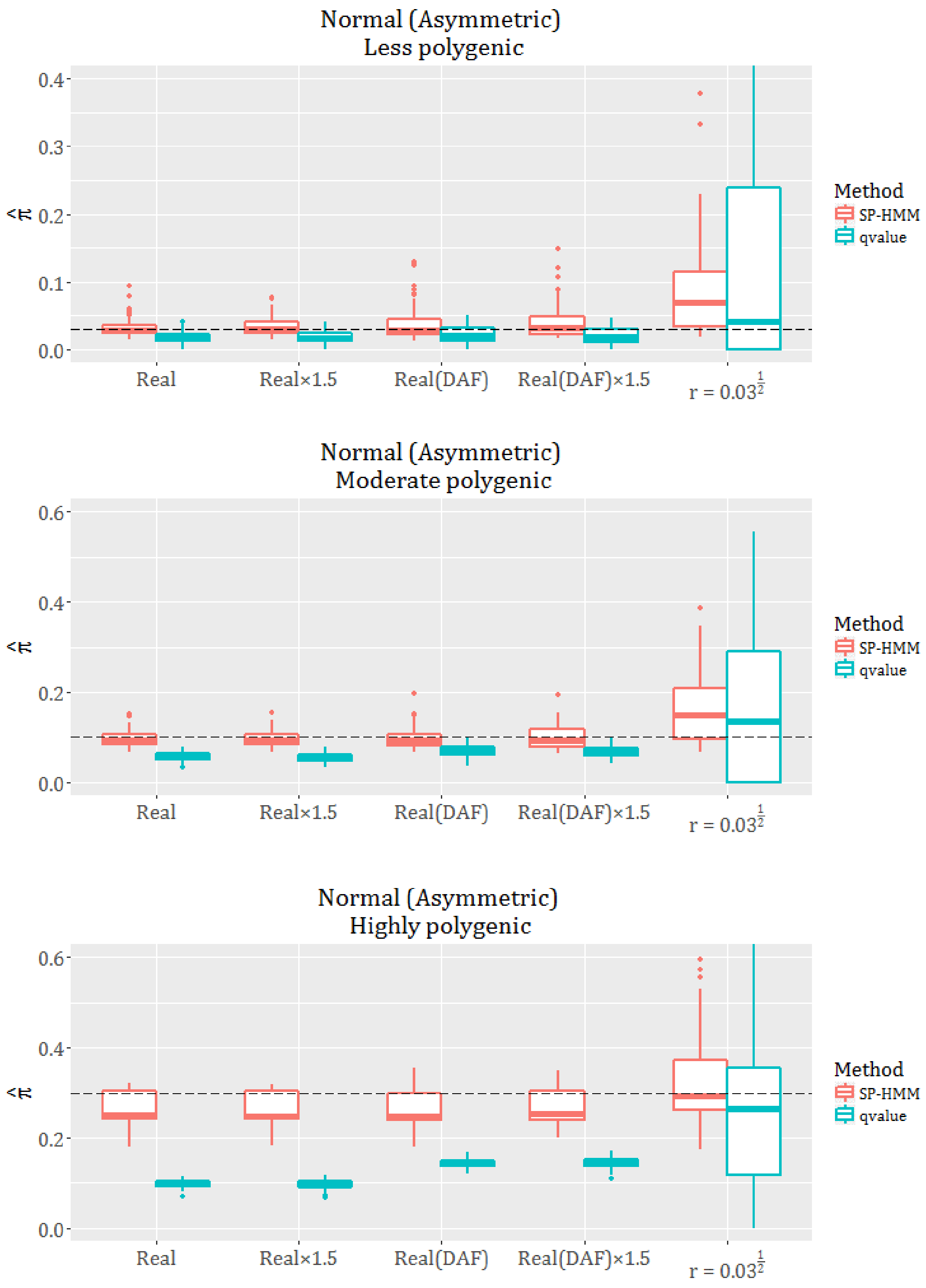


Figure S16. (Continued).

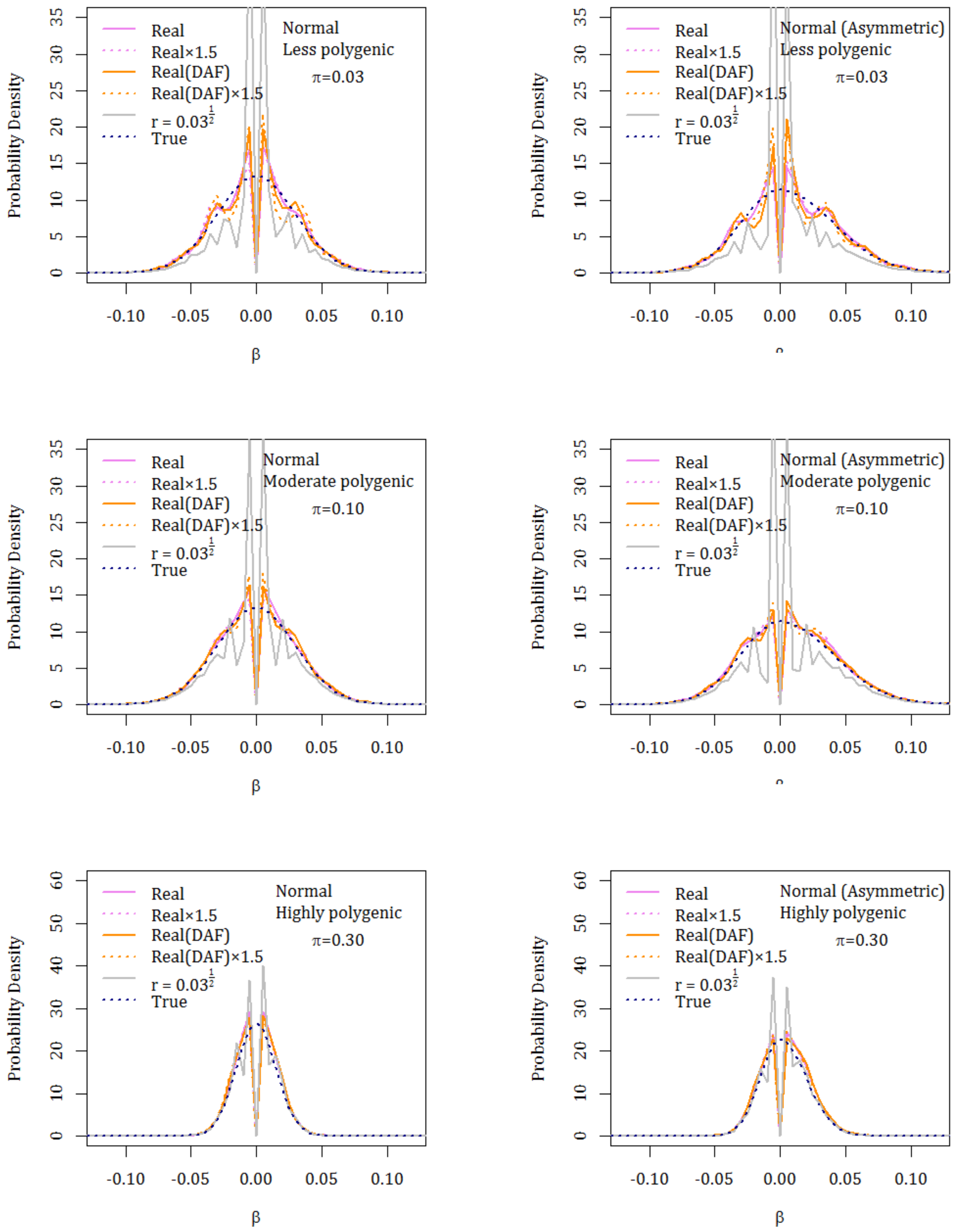


Figure S17. Estimated effect-size distributions, $\hat{g}$, in the settings with SNPs in weak LD. 'Real': Incorporating LD of the P-value based pruned set. 'Real × 1.5': Incorporating 1.5 times stronger LD(r) than that of the P-value based pruned set. 'Real (DAF)': Incorporating LD of the SNP set with $0.4 \leq DAF < 0.6$ for DAF stratified analysis. 'Real (DAF) × 1.5': Incorporating 1.5 times stronger LD(r) than that of the SNP set with $0.4 \leq DAF < 0.6$ for DAF stratified analysis. '$r = 0.03^{1/2}$': Incorporating constant LD among all SNP pairs($r = 0.03^{1/2}$).

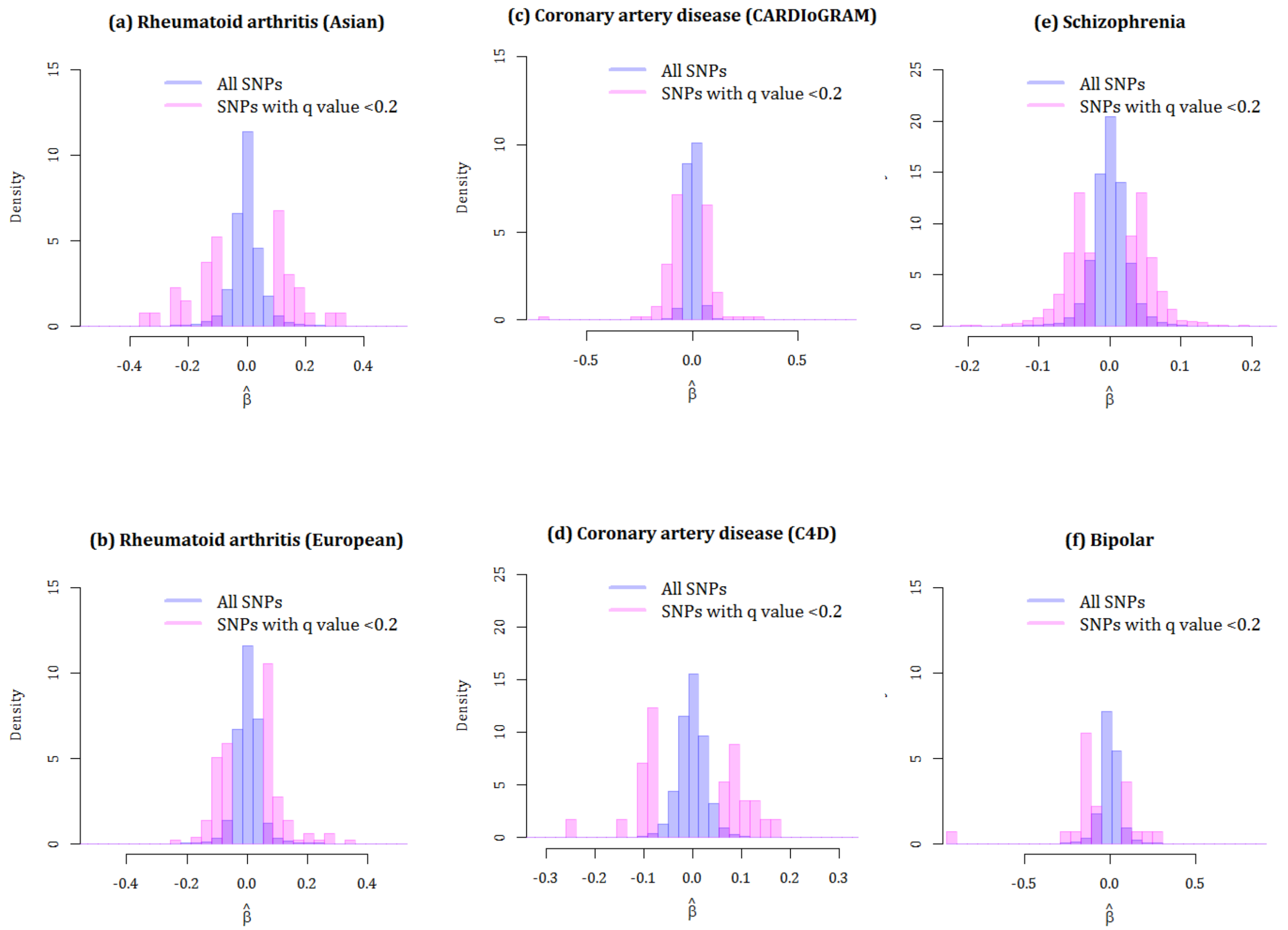


Figure S18. Raw effect size estimates, $\hat{\beta}$, for the P-value-based pruned SNPs in the GWAS summary data . All SNPs : All SNPs in the P-value-based pruned SNPs in each GWAS data. SNPs with q value < 0.2 : SNPs with qvalue < 0.2 in the P-value-based pruned SNPs in each GWAS dataset.

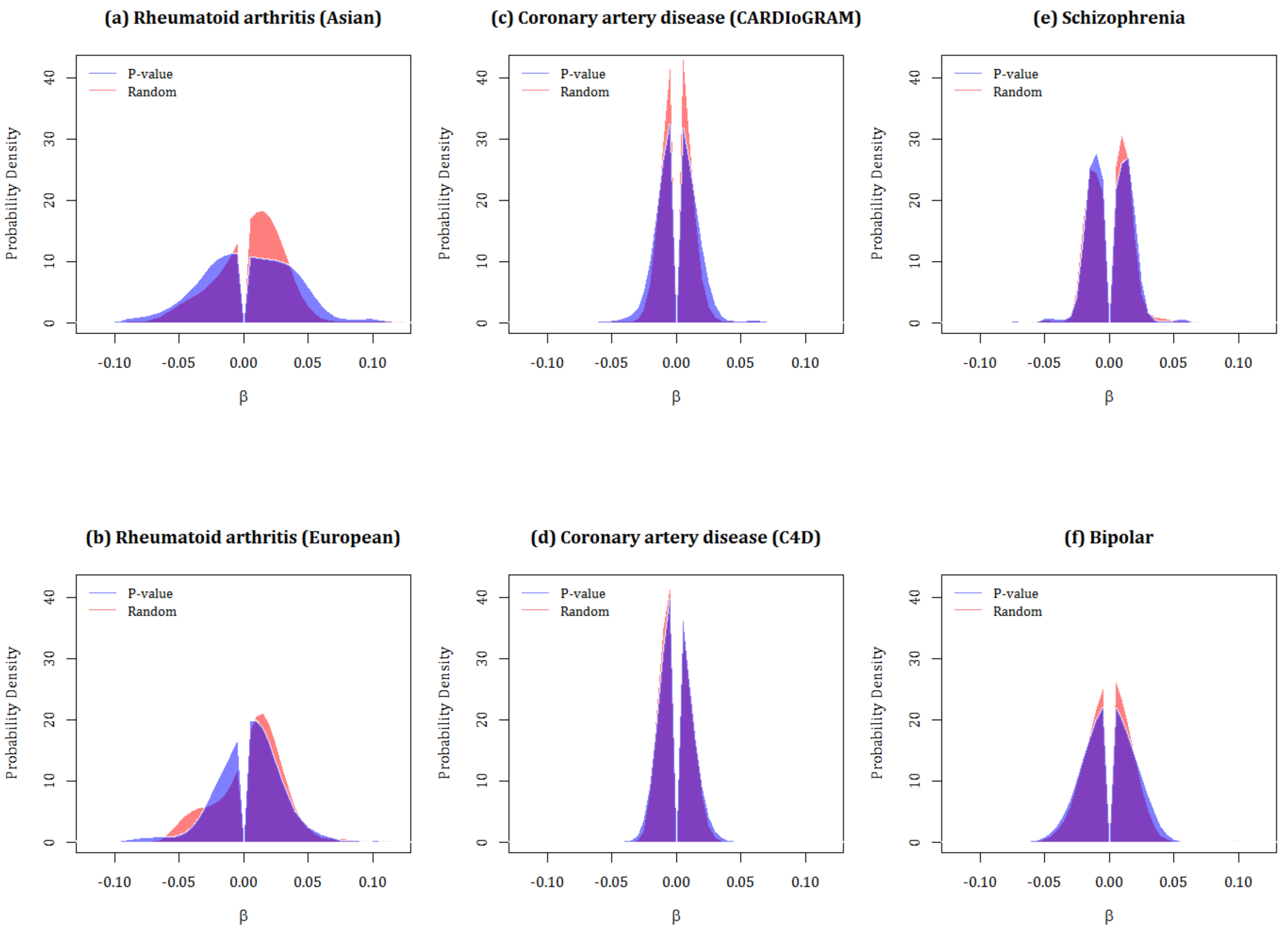


Figure S19. Estimated effect-size distributions for disease-associated SNPs, $\hat{g}$, using the random-pruned SNP sets compared with those using the P-value-based SNP sets. Red and blue lines show the results for the P-value-based and the random-pruned SNP sets, respectively.

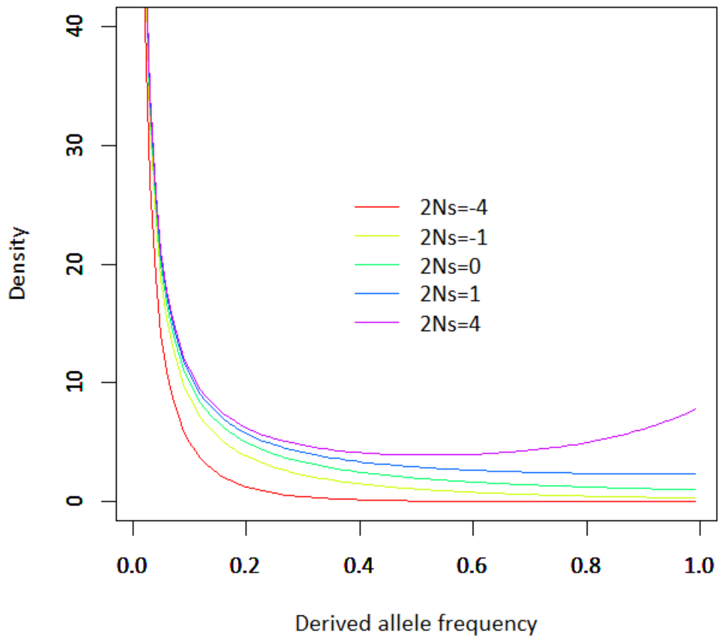


Figure S20. The stationary distributions of derived allele frequency for various values of the selection parameter. Under the Wright-Fisher model with *N* diploids, the fitness of the genotypes *AA*, *Aa*, *aa* are assumed 1, 1+s, 1+2s, where *A* and *a* are ancestral and derived alleles, respectively.

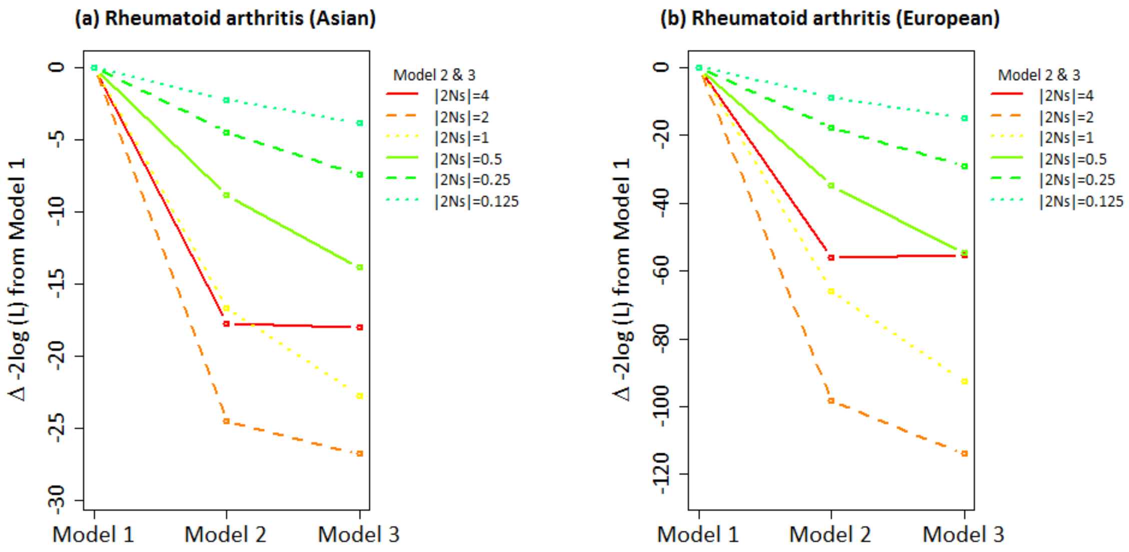


Figure S21. Result of fitting the three selection models. Model 1, neutral mutation model. Model 2, deleterious-risk mutation model. Model 3, deleterious-risk mutation and advantageous-protective mutation model. The difference of twice the negative log-likelihood value from the neutral mutation model (Model 1) is shown on the *y* axis. For bipolar disorder, the two models are also fitted: Model 2', advantageous-risk mutation model; Model 3', advantageous-risk mutation and deleterious-protective mutation model.

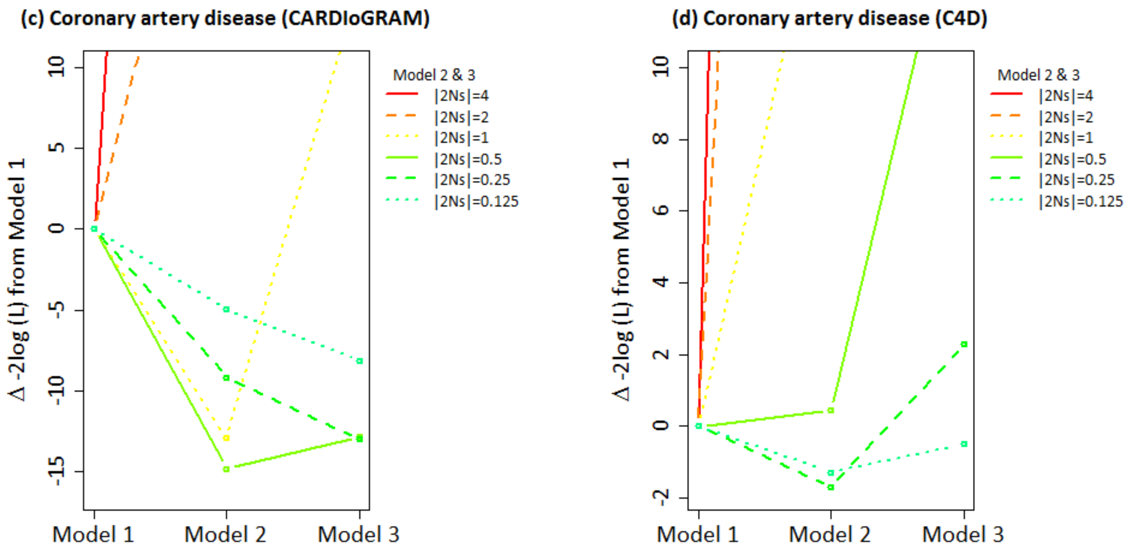


Figure S21. Continued.

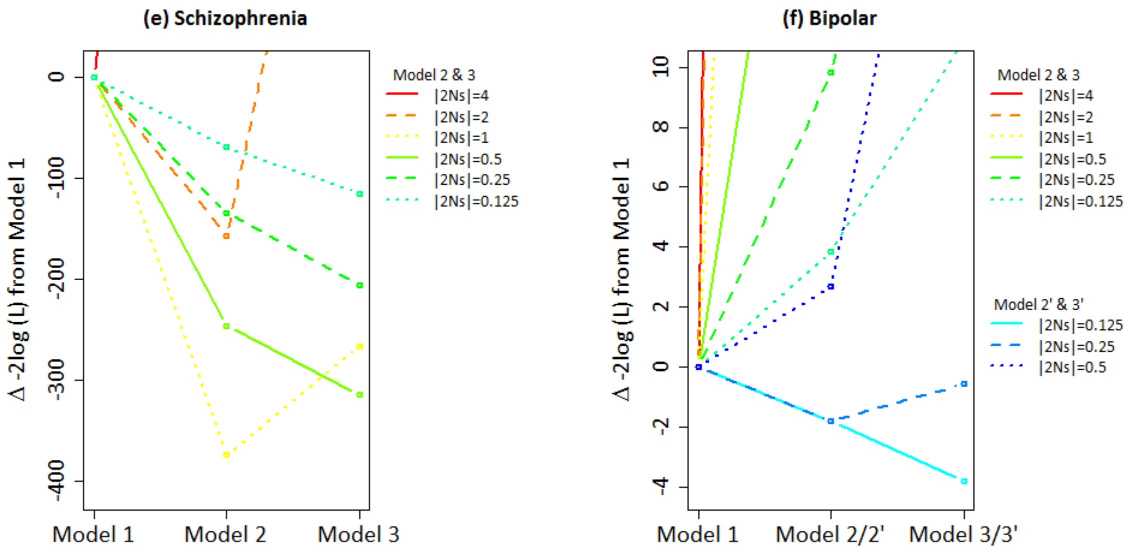


Figure S21. Continued.
